# Exploring the mechanism of action of Sanzi formula in intervening colorectal adenoma by targeting intestinal flora and intestinal metabolism

**DOI:** 10.3389/fmicb.2022.1001372

**Published:** 2022-09-08

**Authors:** Jingyu Shang, Hong Guo, Jie Li, Zhongyi Li, Zhanpeng Yan, Lanfu Wei, Yongzhi Hua, Lin Lin, Yaozhou Tian

**Affiliations:** ^1^Nanjing University of Chinese Medicine, Nanjing, Jiangsu, China; ^2^Affiliated Hospital of Integrated Traditional Chinese and Western Medicine, Nanjing University of Chinese Medicine, Nanjing, Jiangsu, China; ^3^Jiangsu Province Academy of Traditional Chinese Medicine, Nanjing, Jiangsu, China

**Keywords:** colorectal adenoma, Sanzi formula, gut microbiota, colorectal metabolomics, traditional Chinese medicine

## Abstract

**Background:**

*Sanzi formula* (SZF) is a kind of Chinese herbal compound that has a certain effect on the prevention and treatment of colorectal adenoma (CRA), which can prevent and control the process of CRA-cancer transformation. In this study, we explored the mechanism of action of SZF in anti-CRA using 16S rRNA sequencing and metabolomics technology.

**Methods:**

Mice were randomly divided into three groups: Control group, Apc^min/+^ model group, and SZF treatment group. Except for the Control group, which used C57BL/6 J mice, the remaining two groups used Apc^min/+^ mice. The Control group and Apc^min/+^ model group were treated with ultrapure water by gavage, while the SZF treatment group was treated with SZF for 12 weeks. During this period, the physical changes of mice in each group were observed. The gut microbiota was determined by high-throughput sequencing of the 16S rRNA gene, and LC-ESI-MS/MS was used for colorectal metabolomics analysis.

**Results:**

Sequencing of the 16S rRNA gut flora yielded 10,256 operational taxonomic units and metabolomic analysis obtained a total of 366 differential metabolites. The intestinal flora analysis showed that SZF could improve intestinal flora disorders in Apc^min/+^ mice. For instance, beneficial bacteria such as *Gastranaerophilales* significantly increased and harmful bacteria such as *Angelakisella*, *Dubosiella*, *Muribaculum*, and *Erysipelotrichaceae UCG-003* substantially decreased after the SZF intervention. In addition, metabolomic data analysis demonstrated that SZF also improved the colorectal metabolic profile of Apc^min/+^ mice. In Apc^min/+^ mice, metabolites such as *Anserine* and *Ectoine* were typically increased after SZF intervention; in contrast, metabolites such as *Taurocholic acid*, *Taurochenodesoxycholic acid*, *Hyocholic acid*, *Cholic acid*, and *Tauro-alpha-muricholic acid* showed noteworthy reductions. Metabolic flora association analysis indicated that 13 differential flora and 11 differential metabolites were associated.

**Conclusion:**

SZF affects the abundance of specific intestinal flora and regulates intestinal flora disorders, improves colorectal-specific metabolites, and ameliorates intestinal metabolic disorders to prevent and treat CRA. Furthermore, the application of intestinal flora and colorectal metabolomics association analysis offers new strategies to reveal the mechanism of action of herbal medicines for the treatment of intestinal diseases.

## Introduction

As one of the major human cancers possessing the second-highest cancer mortality worldwide, colorectal cancer has a steadily grown morbidity, which is in line with more than 1.8 million new cases every year, and more than 90% of colorectal cancer is transformed from the adenoma-cancer sequence (Keum and Giovannucci, 2019). A colorectal adenoma (CRA) is the most common precancerous lesion of colorectal cancer which will deteriorate into cancer upon a long-term process. Therefore, effective preventive treatment of CRA is crucial for the reduction of colorectal cancer incidence rate (Strum, 2016). In addition, the transformation from adenoma to cancer is not only correlated with adenoma cells but also associated with the homeostasis and metabolism of the intestinal microenvironment, suggesting a possibly potential vital factor – the imbalance of intestinal microbiota (Tilg et al., 2018).

Gut microbiome is an extremely diverse ecosystem composed of 10 ~ 100 trillion microorganisms (about 10 times the number of human cells). It is also an important metabolic system of the human body. The main functions of intestinal flora include metabolic activities, nutritional effects, immunity, and the role of protecting the settled host from foreign microorganisms (Schmidt et al., 2018). It has been previously shown that changes in intestinal microbiota are closely related to CRA (Feng et al., 2015). Similarly, preclinical studies have shown that there is a close correlation between intestinal microbiota and CRA (Kværner et al., 2021). For example, intestinal bacteria such as *Fusobacterium*, *Bacteroides*, *Parvimonas*, and *Prevotella* may have a role in the occurrence of transformation from CRAs to adenoma cancer (Liu W. et al., 2021). Research on colorectal cancer have shown that *Fusobacterium* promotes the transformation of CRA into cancer through enterotoxicity, which is closely bound up with colorectal cancer (Brennan and Garrett, 2019), while *Bacteroides* are also considered to have similar pathogenic effects (Zafar and Saier, 2021). *Parvimonas* with high specificity in colorectal cancer screening is more commonly seen in colorectal cancer and other digestive tumor studies, and it is currently believed that *Parvimonas* may have a potentiating effect with other pathogenic *bacteria* (Coker et al., 2018; Löwenmark et al., 2020). Closely associated with the inflammatory response, *Prevotella* facilitates cancer by stimulating the continuous inflammatory response of the intestine (Ley, 2016). Several studies have suggested that intestinal flora metabolites may directly interfere with the development of CRAs. For example, *C. butyricum* in the intestinal flora can inhibit CRA progression by secreting butyrate and inhibiting the Wnt signaling pathway (Chen et al., 2020). *Colibactin* and *Bacteroides fragilis toxin* secreted by *Escherichia coli* and *enterotoxigenic B. fragilis* may be associated with the promotion of CRA development and transformation to cancer (Dejea et al., 2018). Under pathological conditions, the intestinal microbiota participates in the synthesis of *bile acids*, including *deoxycholate*, which further contributes to the transformation of adenoma into cancer (Yachida et al., 2019).

Specific intestinal flora alterations have recently been reported to obviously contribute to CRA progression (Li L. et al., 2019). Accumulating evidence has indicated that modulation of intestinal microecology may be crucial in the prevention and treatment of CRAs (Yachida et al., 2019).

As a herbal compound formula created by Professor Tian Yaozhou, SZF is based on TCM theory and long-term clinical experience. The formula consists of nine herbs, including Shi Liu Zi (*Semen punicae granati*), Ke Teng Zi (*Entada phaseoloides*), Yu Gan Zi (*Phyllanthi Fructus*), Huang Qi (*Hedysarum multijugum Maxim*), Wu Mei (*Mume Fructus*), Hua Jiao (*Zanthoxyli Pericarpium*), Gou Teng (*Uncariae Ramulus Cumuncis*), Yu Jin (*Curcumae Radix*), and Gan Cao (*licorice*). In the formula, *Mume Fructus* has the effect of regulating intestinal flora and inhibiting inflammation to protect intestinal mucosa. *Hedysarum multijugum Maxim* and *licorice* have effects related to alleviating intestinal flora dysbiosis and improving intestinal barrier dysfunction (Wu et al., 2022). *Entada phaseoloides* have the effect of inducing apoptosis pathway to inhibit cancer cell growth (Zhang et al., 2014). *Phyllanthi Fructus* has hypoglycemic, hypolipidemic, anti-inflammatory, and antioxidant effects. This formula has good efficacy in improving clinical symptoms and reducing the recurrence rate of CRA patients when applied in clinical practice.

In this study, we investigated the mechanism of SZF on CRA from the perspective of intestinal flora and colorectal metabolism through 16S rRNA gene sequencing and metabolomics technology. It will provide new ideas and methods for the rational clinical application of SZF for the treatment of CRA.

## Materials and methods

### Preparation of drugs

SZF was formed from nine kinds of TCM granules, including *Semen Punicae Granati* (shi liu zi), *Entada Phaseoloides* (ke teng zi), *Phyllanthi Fructus* (yu gan zi), *Hedysarum Multijugum Maxim* (Huang qi), *Mume Fructus* (wu mei), *Zanthoxyli Pericarpium* (Hua jiao), *Uncariae Ramulus Cumuncis* (gou teng), *Curcumae Radix* (yu jin), and *Licorice* (gan cao), which were provided by Jiangyin Tianjiang Pharmaceutical Co., Ltd. (Jiangsu, China). The formulated granules were ground into powders and dissolved in warm distilled water before being administered to mice.

### Animals

All animal experiments were performed in compliance with animal welfare guidelines and approved by the Animal Ethical Committee of Jiangsu Hospital of integrated traditional Chinese and Western Medicine (AEWC-202003112-98). Six-week-old male Apc^min/+^ mice and 6-week-old male C57BL/6 J mice were purchased from GemPharmatech Co. Ltd. (Nanjing, China) and under SPF condition with 23 ± 2°C and a 12-h light/dark cycle. The mice have freely accessed a standard diet and drinking water, and the body weight of the mice was recorded every 7 days.

### Gut microbiota detection experiment

To explore the difference of GM under different conditions, after 1 week of acclimatization, the C57BL/6 J mice were divided into one group: the Control group (normal mice administered with ddH_2_O, *n* = 10), and the Apc^min/+^ mice were randomly divided into two groups: the Model group (CRA mice administered with ddH_2_O, *n* = 10) and the SZF group (CRA mice administered with 3.3 mg/g SZF, *n* = 10). The daily dosage for the SZF group was obtained based on the daily dosage for patients (20 g/70 kg) clinically, according to the human-mouse transfer formula (Mouse dose = Human dose × 9.1).

About 400 μl ddH2O was administered to the Control group and the Model group, while 400 μl SZF suspension was administered to the SZF group *via* gastric gavage, and the drug administration lasted for 12 weeks.

The mice’s stools were collected 83 days after administration. 2–3 fecal pellets were collected from each mouse using a 1.5 ml Ep tube and stored at −80°C until used.

### Colorectal metabolomics experiment

To explore SZF metabolites under CRA-induced gut dysbiosis condition, three mice were randomly taken from each group, nine mice in total. At the end of the treatment, colorectal tissues were taken. The changes in colorectal length and the occurrence of CRA were observed. The colorectal tissues were immersed in PBS for cleaning, then half part of the tissues was collected using a 1.5 ml Ep tube and stored at −80°C until used, and the other part was used for HE histopathological staining.

### Gut microbiota analysis

DNeasy PowerSoil Kit (100) (QIAGEN, Dusseldorf, Germany) was used to extract the DNA from fecal samples. The quantity and quality of extracted DNAs were measured using a NanoDrop ND-2000 spectrophotometer (Thermo Fisher Scientific, Waltham, MA, United States) and agarose gel electrophoresis, respectively.

Sequencing of the 16S rRNA genes was performed using the Illumina MiSeq platform. Universal 16S primers (343F/798R) were used to amplify the hypervariable V3-V4 region of bacterial 16S rRNA genes. PCR was performed using thermal cycler Model C1000 (Bio-Rad, Richmond, CA, United States). The 16 s RNA sequencing and data processing were conducted by OE Biotech Co., Ltd. (Shanghai, China).

### Colorectal metabolomics analysis

The colorectal tissues were collected using a 1.5 ml EP tube and stored at −80°C for the Widely Targeted Metabolome (WTM). Liquid chromatography-electrospray ionization-Tandem mass spectrometry (LC-ESI-MS/MS) analysis of colorectal was entrusted to Metware Biotechnology Co., Ltd. (Wuhan, China).

Take out the sample from the −80°C refrigerator and thaw it on ice. Multi-point sample and weigh 20 mg of sample, homogenize (30 HZ) for 20 s with a steel ball, and the centrifuge (3,000 rpm, 4°C) for 30 s. Then, add 400 μl of 70% methanol–water internal standard extractant, shake (1,500 rpm) for 5 min, and place on ice for 15 min. Centrifuge (12,000 rpm, 4°C) for 10 min, transfer 300 μl of the supernatant and stand still it at −20°C for 30 min. Finally centrifuge (12,000 rpm, 4°C) for 3 min and take the supernatant for analysis.

A ExionLC AD UPLC system (AD Sciex, Framingham, MA, United States) coupled with Q-Exactive quadrupole-Orbitrap mass spectrometry (AD Sciex, Framingham, MA, United States) was used to analyze the metabolic profiling in both ESI positive and ESI negative ion modes. Two microliter prepared sample was injected into ACQUITY UPLC HSS T3 column (1.8 μm, 2.1 × 100 mm). All samples were eluted using a linear gradient from 100% mobile phase A (0.1% formic acid in water) to 100% mobile phase B (0.1% formic acid in acetonitrile) under the condition that the flow rate was 400 μl/min and the column temperature was 40°C. Linear gradients 0 min, 5% B; 11 min, 90% B; 12 min, 90% B; 12.1 min, 5% B; 14 min, 5% B. LIT and triple quadrupole (QQQ) scans were acquired on a triple quadrupole-linear ion trap mass spectrometer (QTRAP), QTRAP^®^ LC–MS/MS System, equipped with an ESI Turbo Ion-Spray interface, operating in positive and negative ion mode and controlled by Analyst 1.6.3 software (AD Sciex, Framingham, MA, United States). The ESI source operation parameters were as follows: source temperature 500°C; ion spray voltage (IS) 5,500 V (positive) and −4,500 V (negative); and ion source gas I (GSI), gas II (GSII), and curtain gas (CUR) were set at 55, 60, and 25.0 psi, respectively; the collision gas (CAD) was high. Instrument tuning and mass calibration were performed with 10 and 100 μmol/l polypropylene glycol solutions in QQQ and LIT modes, respectively. A specific set of MRM transitions were monitored for each period according to the metabolites eluted within this period.

The raw data were processed by the Analyst 1.6.3 software (AD Sciex, Framingham, MA, United States) for baseline filtering, peak recognition, peak alignment, and retention time (RT) correction. The self-built database MWDB (Metware database, Metware Biotechnology Co., Ltd., Wuhan, China) was used to identify the compounds.

### Statistical analysis

Statistical analysis was performed using GraphPad Prism 9.3.1(471) for Windows and differences were considered significant at a *p* < 0.05. Distributions of variables were examined by Shapiro–Wilk Test according to our sample size and appropriate tests were applied for further analysis. The changes of colorectal length, Count of CRAs, and the changes of body weight of mice were analyzed with analysis of variance (ANOVA). Means from the data, together with estimates of the standard error of the mean and pairwise comparisons (Tukey’s or Games-Howell test), were obtained. Clustering of gut microbial communities among different groups was analyzed with Adonis. The alpha diversity analysis was conducted by ANOVA, while the beta diversity analysis was analyzed with Adonis. In the analysis of colorectal mass spectrometry data, the partial least squares discrimination analysis (PLS-DA) was used to observe the distribution of samples and the stability of the analysis process, and then the supervised orthogonal PLS-DA (OPLS-DA) was used to describe the difference between groups. The contribution of each variable to the population was ranked according to the variable importance of projection value (VIP value) obtained from the OPLS-DA model, and the significance of differential metabolites between groups was verified by fold change analysis. The Pearson’s correlation coefficient was used to measure the linear correlation between the two groups. It was considered that the difference was statistically significant when VIP > 1.0 and fold change >2 or fold change <0.5.

## Results

### SZF inhibits the progression of colorectal adenomas

**Figure 1:**
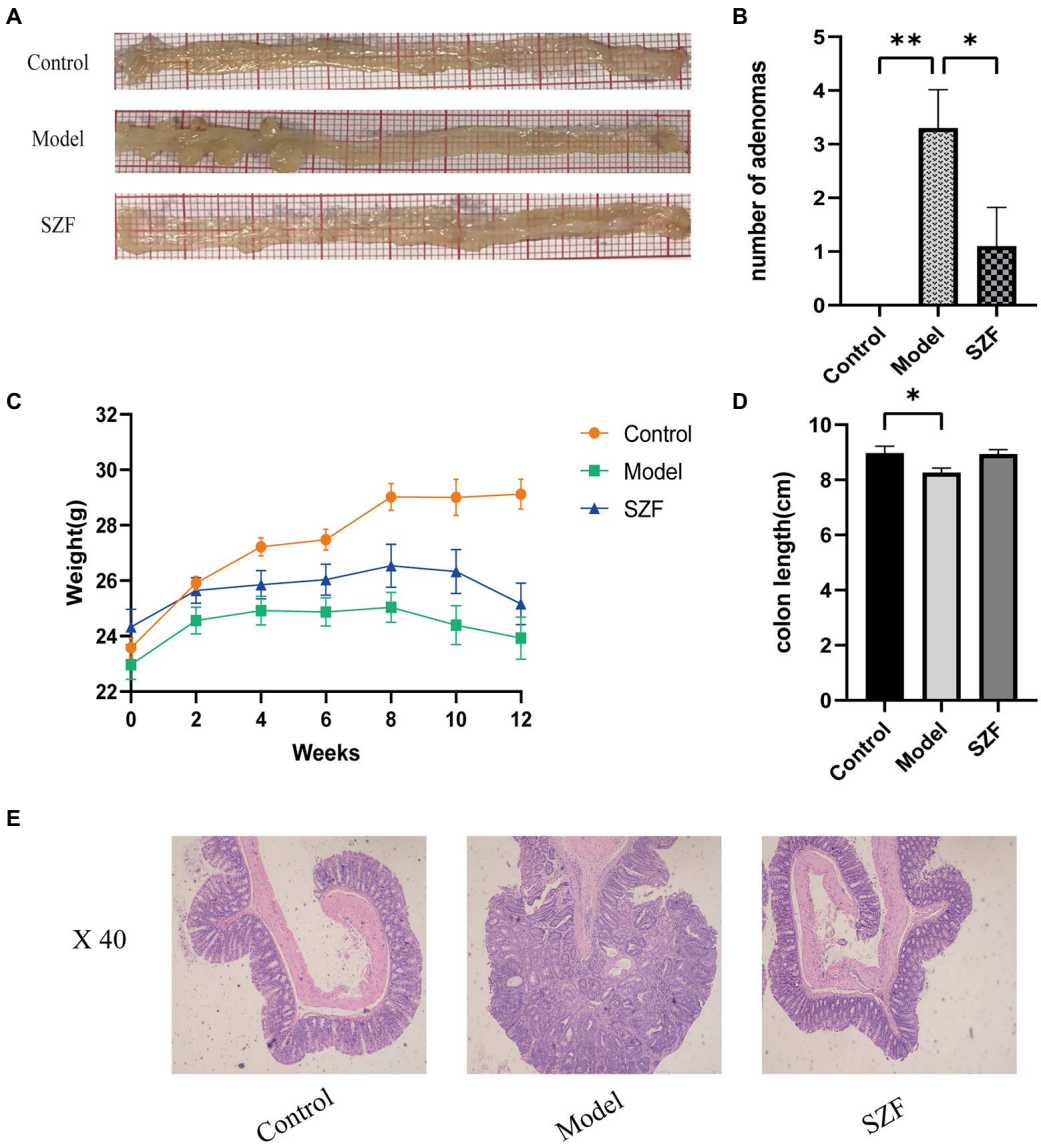
Effect of SZF on morphological changes of Colorectal Adenoma in mice. **(A)** Representative images of colorectal tissue in each group. **(B)** Statistical analysis of the number of adenomas, ^*^p < 0.05; ^**^p < 0.01. **(C)** Change of body weight of mice in each group. **(D)** Statistical analysis of colonic length in each group of mice, ^*^p < 0.05. **(E)** Results of HE staining in colorectal tissue of each group.

In this study, an autologous tumor model of Apc^min/+^ mice was selected. After oral administration of SZF, the number of adenomas in the treated mice was significantly reduced (*p* < 0.05) (Figures 1A,B) and their body weight was fairly improved compared to the model group, converging to the weight of the control group (Figure 1C). The average colorectal length of the control group was 8.97 cm and that of the model group was 8.26 cm. After treatment with SZF, the length of the colorectum was meliorated (*p* > 0.05), demonstrating that SZF reduced the inflammatory response of the intestine (Figure 1D). Additionally, the histological results of colorectum showed that the colorectal glands in the control group were normal in structure, with clear mucosal layer, no mucosal ulcer, and no heterogeneity of intestinal epithelium. In the model group, the colorectal glands were densely arranged with a sieve-like structure, and the epithelial cells of the glands were obviously dysplastic, with distinctly enlarged cell nucleus, and pathological mitosis could be seen. The histological examination of the colon from the mice in the SZF treatment group revealed normal glandular structure and no obvious heterogeneity in glandular epithelial cells (Figure 1E).

### Effect of SZF on the composition of intestinal flora in colorectal adenoma mice

**Figure 2:**
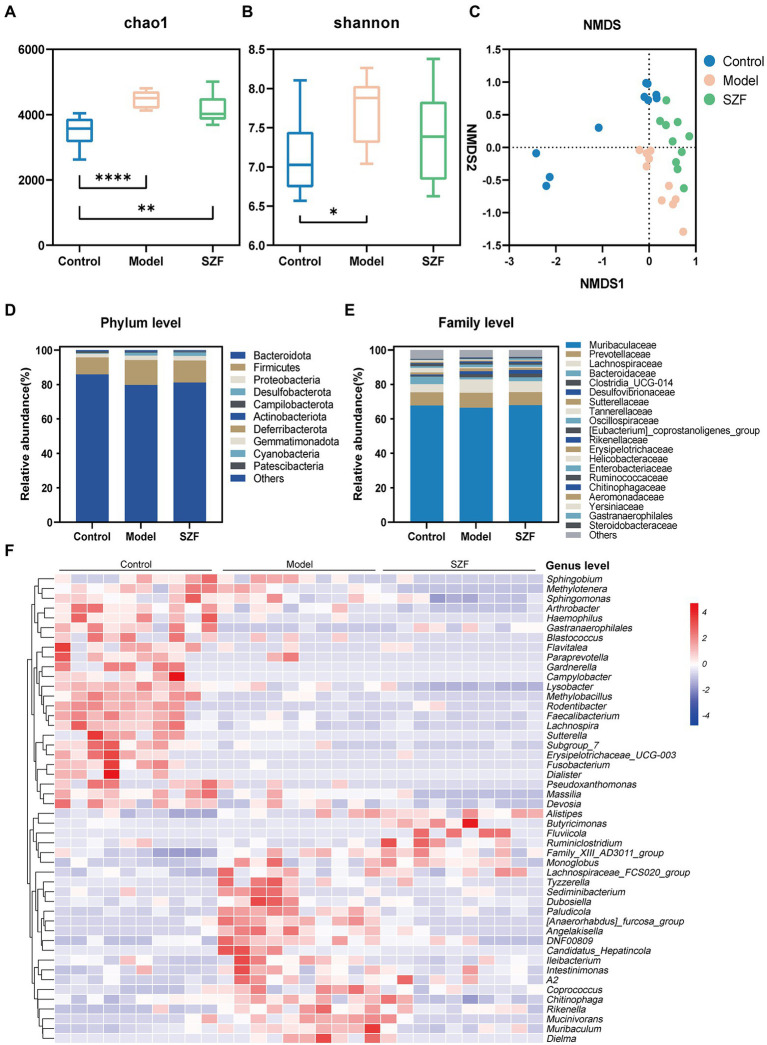
Analysis of intestinal microbial diversity and species composition. The alpha diversity of intestinal microorganisms was evaluated through Chao1 **(A)** and Shannon **(B)** index. The beta diversity of intestinal microorganisms was evaluated through NMDS **(C)**. Relative abundances of the main phyla **(D)**, families **(E)** and genera **(F)** of intestinal microbiota in different groups. The results of data statistics were expressed by means ± SEM. ^∗^*p* < 0.05, ^∗∗^*p* < 0.01, ^∗∗∗∗^*p* < 0.0001.

To further investigate the effect of SZF on intestinal flora, the fecal microbiota of three groups of 30 samples were analyzed by 16S rRNA sequencing. Based on the 97% similarity level, 10,256 operational taxonomic units were finally obtained. According to the trends of individual sparsity curves, Shannon-Wiener curves, rank-abundance distribution curves, and species accumulation curves, the sequencing data were sufficient to reflect the microbial information in all samples. Microbial α-diversity was assessed using Shannon and Chao1 index fecal samples. Both Chao1 and Shannon indices were remarkably increased in the model group mice compared to the blank control group (Figures 2A,B). There was a tendency for the Chao1 and Shannon indices to decrease after treatment with the SZF. In terms of β-diversity, non-metric multidimensional scaling analysis was used to assess the differences in gut microbiota between the three groups. The results showed that the blank control group was separated from the model group, and there was a noteworthy trend of separation between the SZF treatment group and the model group (Figure 2C). These results suggest that the intestinal flora of CRA model mice is dysregulated, and that SZF can interfere with the abundance and diversity of the flora.

Next, the intestinal flora was assessed at the taxonomic level of phylum, family, and genus. At the phylum level, there was no significant difference between the groups (Figure 2D). At the family level, the abundance of *Steroidobacteraceae*, *Yersiniaceae*, *Chitinophagaceae*, and *Aeromonadaceae* was significantly higher in the model group than in the blank control group and was typically lower after the SZF intervention. In addition, the abundance of *Gastranaerophilales* in the model group was substantially reduced, and the trend could be reversed by the SZF (Figure 2E). We used heat map analysis to observe the changes in bacterial populations between the three groups at the genus level. At the genus level, 48 bacterial species differed between the model group and the blank control group, 14 of which were affected by the SZF. Compared to the blank control group, the model group had *(Anaerorhabdus)_furcosa_group*, *Paludicola*, *Angelakisella*, *DNF00809*, *Muribaculum*, *Erysipelotrichaceae UCG-003*, *Gardnerella Mucinivorans*, *Sutterella*, *Tyzzerella*, *Pseudoxanthomonas*, *Sediminibacterium*, *Fluviicola*, and *Dubosiella* significantly increased in abundance (Figure 2F). In contrast, SZF obviously reversed the abundance of these bacteria.

**Figure 3:**
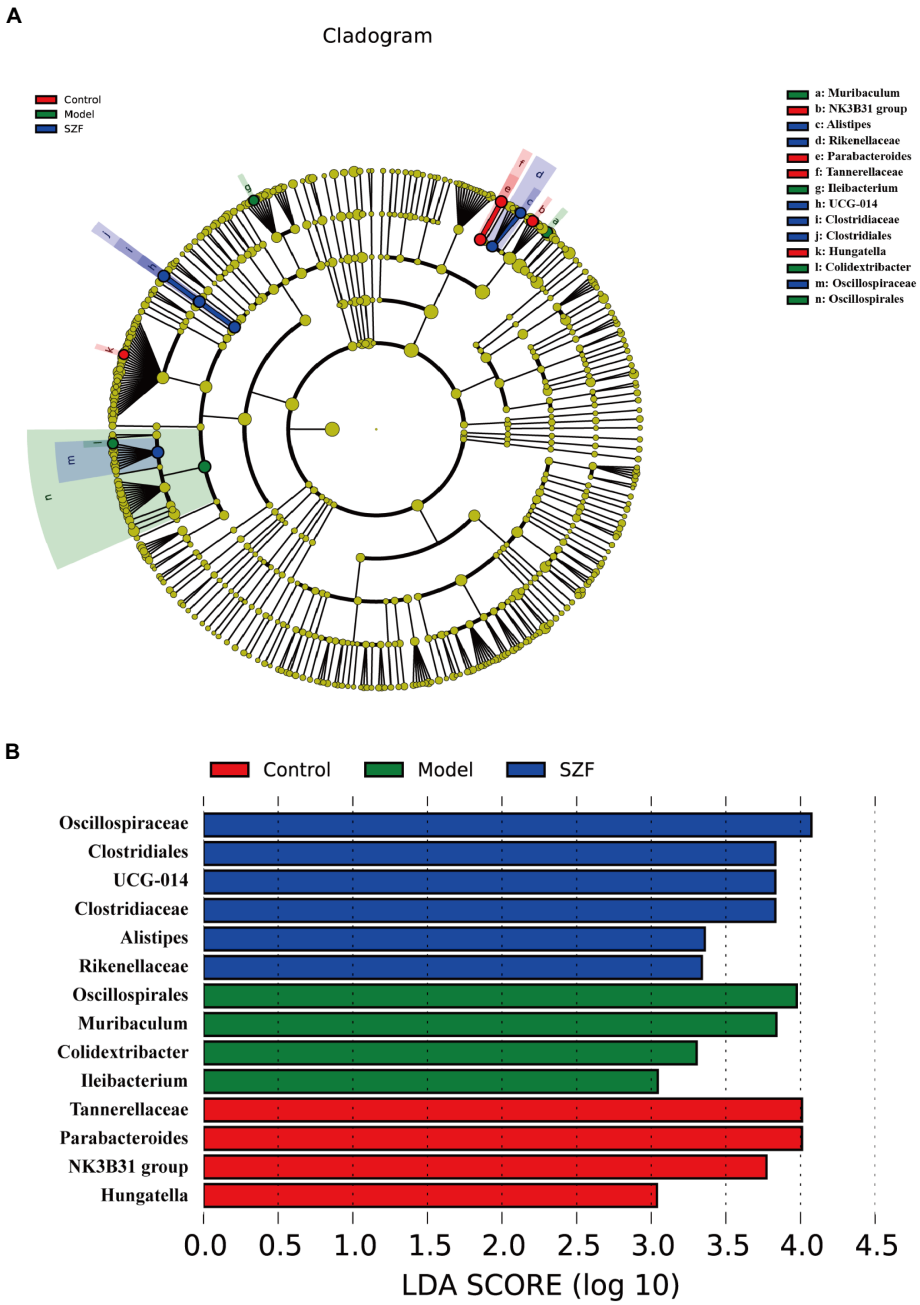
Linear discriminant analysis (LDA) effect size. A cladogram showed specific bacteria among three groups **(A)**. LDA showed scores of these specific bacteria **(B)**.

Using the linear discriminant analysis effect size method to identify specific bacterial taxa, a total of 14 specific bacteria (LDA score > 3) were divided into three groups. 4 strains of specific bacteria in the blank control group, 4 strains in the model group, and 6 strains in the SZF group (Figure 3). These phylotypes played a key role in differentiating the composition of the three groups of gut microbiota.

### Modulation of intestinal metabolism in CRA mice by SZF

**Figure 4:**
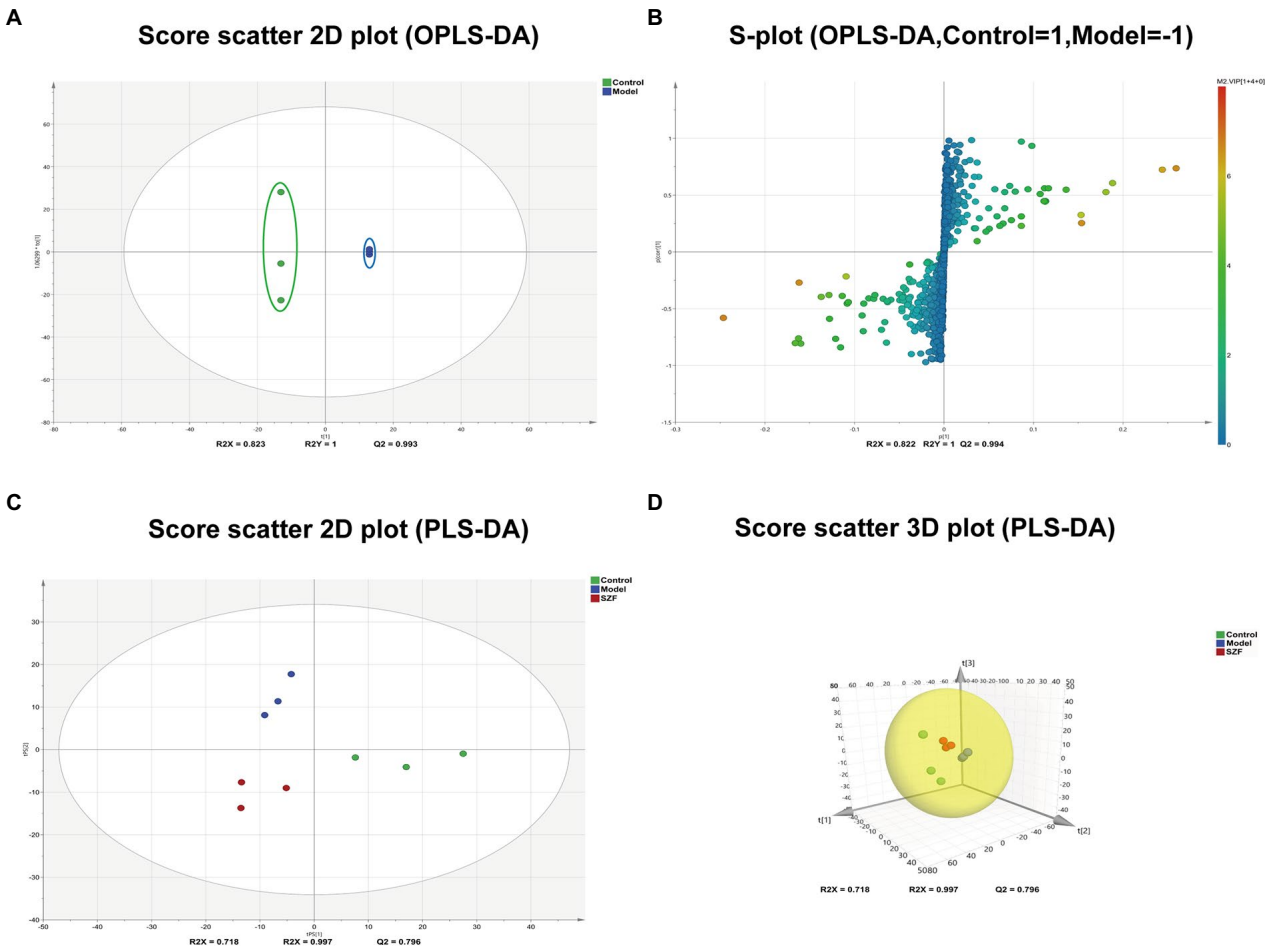
Multivariate statistical analysis and differential metabolic screening. Score scatter plot **(A)** and S-plot **(B)** were obtained by OPLS-DA analysis between control and model groups. Score scatter 2D **(C)** and 3D **(D)** plot of each group were obtained by PLS-DA analysis.

To initially explore the actual situation of intestinal flora metabolism in each group, we randomly drew 3 samples from each group, a total of 9 samples, and analyzed colorectal tissue samples by LC-ESI-MS/MS system in positive and negative ion models, and obtained the corresponding metabolic fingerprints of each group. Next, data were imported into SIMCA-P for multivariate statistical analysis by Analyst 1.6.3. Differences in colorectal metabolic profiles between groups were assessed by PLS-DA. The SZF group was distinctly separated from the model group, suggesting that SZF can modulate metabolism in model mice (Figures 4C,D).

In the next step, potential biomarkers were screened between the normal and model groups based on OPLS-DA. Scatter plots of OPLS-DA scores showed a complete separation of metabolic profiles between the blank control and model groups (Figures 4A,B). A combined plot of loaded S-plot and VIP values was used to identify differential metabolites. Hinged on the constraints (VIP > 1, fold change ≥2 and fold change ≤0.5), 124 features were obtained as potential differential metabolites associated with CRAs.

**Figure 5:**
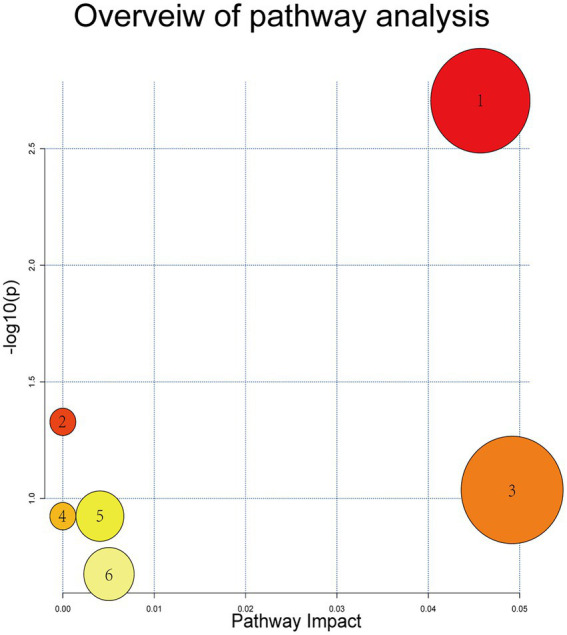
Metabolic pathways of potential biomarkers. (1) Primary bile acid bio-synthesis; (2) taurine and hypotaurine metabolism; (3) histidine metabolism; (4) beta-alanine metabolism; (5) sphingolipid metabolism; and (6) pyrimidine metabolism.

Based on MS spectral information and the self-built targeting marker database Metware database (MWDB), 11 potential biomarkers were eventually identified by qualitative analysis according to the RT, daughter and parent ion pair information, and secondary spectrum data. The metabolites visibly increased in the model group compared with the blank control group included *Deoxycytidine*, *Methacholine*, *N-Acetyl-D-Galactosamine, Taurocholic acid*, *Taurochenodesoxycholic acid*, *Hyocholic acid*, *Cholic acid*, *Tauro-alpha-muricholic acid*, and *Phytosphingosine*, while colorectal metabolites distinctly reduced in model mice were *Anserine* and *Ectoine*. Levels of these differential metabolites were overtly reversed after treatment with the SZF. Metabolic pathway analysis was performed using MetaboAnalyst online software^1^ and the KEGG database.^2^ On the basis of 11 identified biomarkers, 6 metabolic pathways were attained, 2 of which were selected as the most essential metabolic pathways associated with metabolic disorders (*p* < 0.05, effect >0.1). These metabolic pathways included Primary bile acid biosynthesis, Taurine and hypotaurine metabolism (Figure 5).

### Correlation between intestinal flora and colorectal metabolites

**Figure 6:**
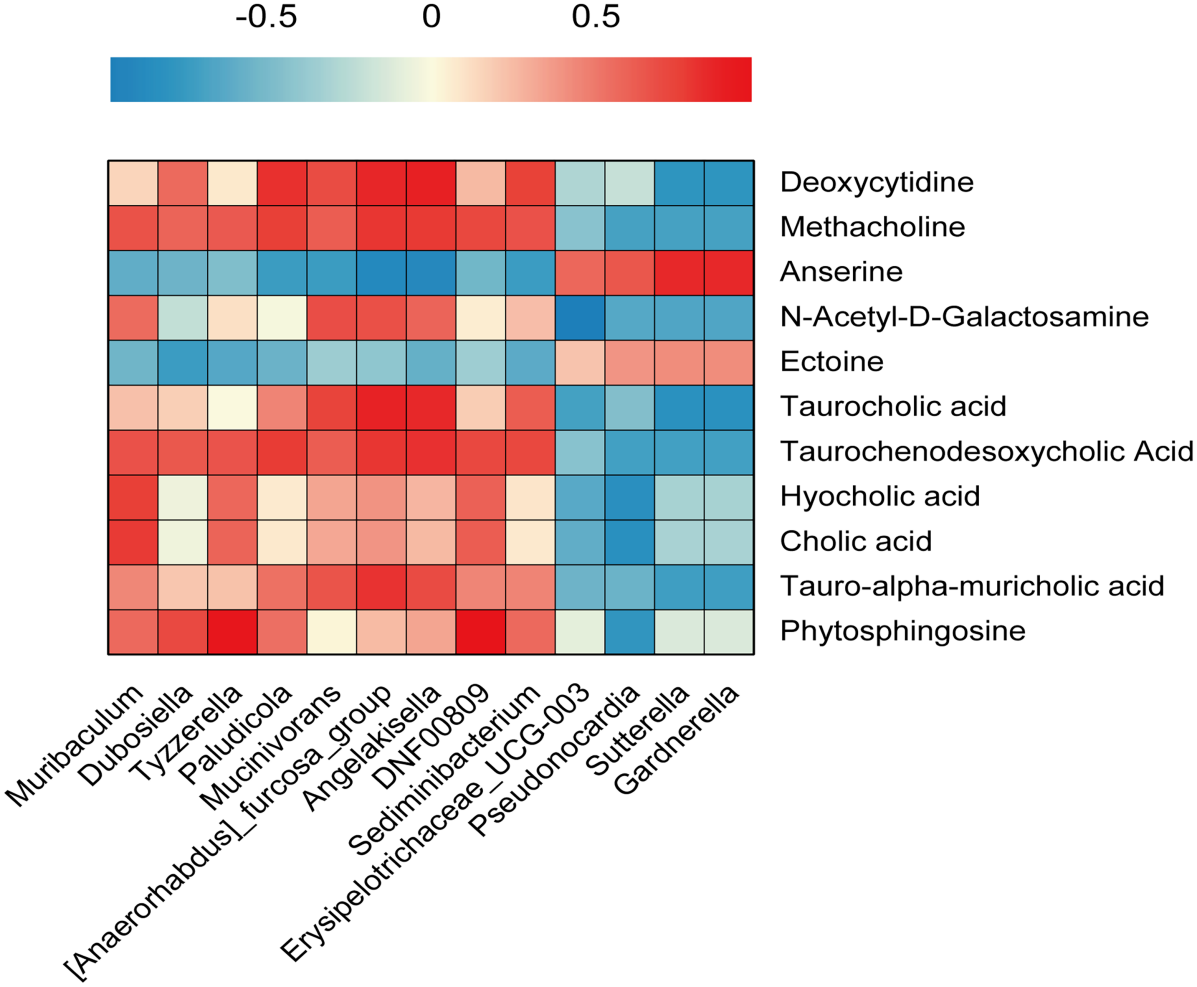
Gut microbiota and colorectal metabolomics. Pearson’s correlation analysis was performed between Gut microbiota abundance and colorectal metabolomics.

To explore the relationship between intestinal flora and colorectal metabolites, Pearson’s correlation analysis was performed and a heat map of correlation coefficients was achieved. The results indicated that bacteria such as *Sediminibacterium*, *Muribaculum*, *Angelakisella*, and *DNF00809* were positively correlated with nine metabolites, including *Taurocholic acid*, *Taurochenodesoxycholic acid*, *Methacholine*, *Tauro-alpha-Muricholic acid,* and *Phytosphingosine*, etc. *Pseudonocardia*, *Erysipe-lotrichaceae_UCG-003*, *Sutterella*, *and Gardnerella* were positively associated with *Anserine* and *Ectoine* (Figure 6).

## Discussion

Despite the strong association of colorectal carcinogenesis with genetic factors, the vast majority of bowel cancers are sporadic, with more than 90% of them being transformed by the adenoma-carcinoma sequence (Keum and Giovannucci, 2019). The adenoma-carcinoma sequence is a long-term and slowly progressive process, so how to effectively prevent and treat CRAs becomes an imperative part of reducing the incidence of colorectal cancer. Currently, the treatment of CRA is still limited to colonoscopy and surgical resection, and there are no practical and effective pharmacological means to prevent and treat recurrence after surgery. With the in-depth study of intestinal flora, there is an increasing number of evidence that the development of diseases may be closely related to intestinal flora, and this theory has been widely applied to the study of intestinal diseases, obesity, diabetes, and other fields (Liu X. et al., 2021; Liu B. et al., 2021). Therefore, regulation of intestinal microecology may be a potential research direction for the prophylaxis and treatment of CRAs.

There is growing evidence that traditional Chinese medicine (TCM) have a dominant role in regulating intestinal microecology. TCM have the ability to modulate gut microbial homeostasis and influence disease progression by altering the expression of specific flora, in a process in which TCM can both adjust gut flora-associated metabolites and herbal compounds can be transformed through the associated gut flora (Feng et al., 2019). As the second largest TCM system in China, Tibetan medicine has a long history of more than 2,400 years and a complete theory, which is widely used in the prevention and treatment of cancer, precancerous lesions, and other related fields (Fu et al., 2020). SZF is a Chinese herbal compound formula founded by Professor Tian Yaozhou rests on the theory of Chinese medicine and long-term clinical experience. Under the guidance of TCM theory, the formula combines the ideas of Tibetan medicine on tumor prevention and cure, and uses three Tibetan herbs with tumor-preventing effects, namely *Semen punicae granat*, *Entada phaseoloides*, and *Phyllanthi Fructus*. Modern research has shown that *gallic acid* and *Ellagic acid*, the main active ingredients of *Semen punicae granati*, can target and regulate some apoptosis pathways and have anti-cancer activity. *Ellagic acid* is also one of the meaningful active ingredients of *Phyllanthi Fructus* (Tang et al., 2020). Meanwhile, *Entada phaseoloides* plays a role in the process of inducing apoptosis pathway to inhibit cancer cell growth (Zhang et al., 2014). This formula has been applied clinically and has good efficacy in improving clinical symptoms and reducing the recurrence rate of CRA in patients with CRA. As mentioned earlier, several herbs in the SZF have the effect of interfering with intestinal flora homeostasis, and CRA-cancer transformation is also firmly related to the alteration of intestinal flora. A few metabolites such as *butyrate* and *bile acids* synthesized by intestinal flora have significant influences on CRA and even colorectal cancer (Winston and Theriot, 2020). Accordingly, the present study attempted to explore the role of SZF against CRA through intestinal flora as well as colorectal metabolome.

Apc^min/+^ mouse model is a classical model used to investigate CRA. In this experiment, in consideration of the possible impacts on the intestinal flora and related changes of metabolites in mice induced by the gastrointestinal administration of chemical agents, we adopted Apc^min/+^ mouse model. Therefore, the Apc^min/+^ mouse model was chosen to explore the mechanism of action of SZF on the intestinal flora of CRA as well as colorectal metabolites. Here we showed that compared with the Control group, the mice in the model group had obviously reduced body weight, bearing varying degrees of blood stools. Anatomically, CRAs were found, and pathological manifestations of adenomas such as dense arrangement of glands, cribriform structure, and obvious dysplasia of gland epithelial cells were presented. In contrast, the group using SZF was apparently more inclined to healthy mice.

In this study, we also observed that the intestinal flora of the model was disturbed and the treatment with SZF could improve the intestinal microecology. SZF significantly reduced the abundance of *Angelakisella*, *Dubosiella*, *Muribaculum*, *Erysipelotrichaceae UCG-003*, and *Sutterella* in the intestinal flora of CRA model mice. The increased abundance of *Angelakisella* and *Dubosiella* is thought to be closely linked to intestinal inflammation and intestinal microbial disorders (Qiu et al., 2021). High expression of *Muribaculum* has been suggested as a possible marker of *bile acid* metabolism disorders (Marion et al., 2020). *Erysipelotrichaceae UCG-003* has been reported to be associated with disorders of *bile acid* metabolism as well as intestinal dysfunction (Xu et al., 2022), which is also related to the induction of intestinal inflammation (Cheng et al., 2021) and tumor development (Zhao et al., 2021). *Sutterella* has been mentioned more in Crohn’s disease and inflammatory bowel diseases in recent years. Although it does not have a clear role in causing inflammation, it has been reported that increased *Sutterella* abundance provides a better environment for proliferation of inflammatory bacteria, thus promoting an inflammatory response (Kaakoush, 2020). SZF can substantially improve the abundance of *Gastranaerophilales* in CRA model mice. It has been reported that *Gastranaerophilales* is a vital bacterium for Indole production, which further promotes the synthesis of *indolepropionic acid* (IPA), and has anti-inflammatory and cancer-inhibiting effects in gastrointestinal IPA (Rosario et al., 2021). Hence, it is reasonable to assume that SZF has a role in preventing CRAs by promoting beneficial bacteria and inhibiting harmful bacteria, thus restoring intestinal microecology.

LC-ESI-MS/MS-based metabolomics has been widely used to study various groups of disease-related biomarkers. In order to investigate the relevant effects on colorectal metabolism after improving intestinal microecology with SZF treatment, WTM analysis has been adopted to screen for differential metabolites in colorectal tissues. Finally, 11 metabolites were identified in colorectal tissue samples, which were involved in six metabolic pathways. In addition, both Primary bile acid biosynthesis and Taurine and hypotaurine metabolism metabolic pathways were disturbed in CRA model mice, and SZF could typically improve these metabolic pathways. Anserine level in CRA model mice was increased by SZF, and previous studies have confirmed the tumor suppressive effects of anserine in colorectal cancer patients, which may be closely related to its antioxidant and anti-inflammatory effects (Wu, 2020). Compared with the blank control group mice, the levels of *Taurocholic acid*, *Taurochenodesoxycholic acid*, *Hyocholic acid*, *Cholic acid*, and *Tauro-alpha-muricholic acid* were increased in the rectal adenoma mouse model group, while the application of the SZF fairly lowered the levels of these metabolites. Reports indicated that expression of *Taurocholic acid* is closely correlated with the regulation of bile acid structure and can coordinate intestinal flora, thus promoting colorectal inflammation (Devkota et al., 2012). *Taurochenodesoxycholic acid* is deemed to have close connection with colorectal inflammation (Kolho et al., 2017), as its expression is elevated in ulcerative colitis and Crohn’s disease. *Hyocholic acid* is an important metabolite that regulates bile acid metabolism, and its elevated expression is a considerable marker for disorders of bile acid metabolism (Zheng et al., 2021). *Cholic acid* enhances the invasion of colorectal cancer cells by activating mmp9-related signaling pathways (Li et al., 2020). *Tauro- alpha-muricholic acid*, on the other hand, acting as an imperative FXR receptor antagonist, has a role in facilitating colitis and colon cancer. Many studies have demonstrated that *Hedysarum multijugum Maxim* and *licorice*, crucial components of SZF, have relevant effects in reducing the levels of bile acids such as *Taurocholic acid*, *Taurochenodesoxycholic acid*, *Hyocholic acid*, and *Cholic acid*, and in regulating the disorders of bile acid metabolism (Wu et al., 2017; Li W. et al., 2019).

In conclusion, bile acid metabolism is closely related to intestinal flora disorders and CRA-carcinoma transformation. The above experimental results suggest that SZF may prevent and treat CRA by regulating bile acid metabolism. Besides, the expression of *Deoxycytidine*, *N-Acetyl-D-Galactosamine*, and *Phytosphingosine*, all of which have some degree of toxicity and mucosal irritation, was elevated in the CRA model, while the high expression of these metabolites is considered to be closely tied with colorectal cancer (Goedert et al., 2014; Brown et al., 2016; Sun et al., 2021), and all showed a decreasing trend in expression after treatment with SZF. It has been reported that *Mume Fructus*, a vital component of SZF, has effects of maintaining microbial homeostasis, inhibiting the differentiation of CD4+ T cells in intestinal mucosa and secretion of pro-inflammatory cytokines such as TNFα (*tumor necrosis factor α*), IL-1β (*interleukin 1β*), γ-IFN (*interferon γ*) and IL-17A (*Interleukin 17A*), so as to improve intestinal mucosal inflammation (Wu et al., 2022). Therefore, SZF may suppress the progression of the adenoma-carcinoma sequence by inhibiting the intestinal inflammatory response and modulating the tumor microenvironment. Combined with the results of metabolomic analyses and studies on the role of the important components of SZF, it is suggested that SZF may prevent and control CRAs by regulating the disturbance of bile acid metabolism, inhibiting the colorectal inflammatory response, and reducing toxic metabolites of the intestinal flora.

In this study, we comprehensively evaluated the correlation between intestinal flora and colorectal metabolites. Three bile acids (*Taurochenodesoxycholic Acid*, *Hyocholic acid,* and *Cholic acid*) and two colorectal cancer-related metabolites (*Methacholine* and *N-Acetyl-D-Galactosamine*) were positively correlated with *Muribaculum*. The high expression of *Muribaculum* mentioned above is regarded as one of the possible markers of bile acid metabolism disorder, which coincides with the elevated expression of *Hyocholic acid* as one of the important markers of bile acid metabolism disorder. The relative abundance of *Muribaculum* as well as the metabolites tended to decrease after treatment with SZF. *Deoxycytidine*, *Methacholine,* and *Taurochenodesoxycholic Acid* were positively correlated with *Angelakisella* and *Dubosiella*. As above mentioned, *Angelakisella* and *Dubosiella* are closely associated with intestinal inflammation, *Taurochenodesoxycholic Acid* remains intimately associated with two important inflammatory bowel diseases (ulcerative, Crohn’s disease), and Methacholine is a common toxic metabolite, which once again confirms the two flora and intestinal inflammation, indicating that *Angelakisella* and *Dubosiella* may promote colorectal inflammatory changes through the synthesis of metabolites such as *Taurochenodesoxycholic Acid* and *Methacholine*. The relative abundance of *Angelakisella* and *Dubosiella* as well as the related metabolites tended to decrease after treatment with SZF. In general, SZF may achieve the prevention and treatment of CRA by interfering with the relevant intestinal flora, thus regulating the disturbance of bile acid metabolism and reducing the synthesis of related pro-inflammatory metabolites.

## Conclusion

In summary, Apc^min/+^ mouse CRA model was selected and applied to study the therapeutic effect of SZF against CRA. The results showed that SZF has a good effect in preventing and treating CRA. In addition, 16S rRNA gene sequencing and metabolomic analysis of colorectal tissues showed that SZF significantly improved the dysbiosis of intestinal flora and disorder of colorectal metabolite profile in CRA mice, and the reduced beneficial bacteria, such as *Gastranaerophilales*, notably increased in CRA mice after SZF treatment, while the harmful bacteria, such as *Angelakisella*, *Dubosiella*, *Muribaculum*, and *Erysipelotrichaceae UCG-003*, were prominently reduced. Moreover, colorectal metabolomic analysis indicated that colorectal metabolic profile was improved after the treatment with SZF, which significantly increased anti-inflammatory metabolites, such as *Anserine* and *Ectoine*, and significantly decreased harmful metabolites, such as *Taurocholic acid*, *Taurochenodesoxycholic acid*, *Hyocholic acid*, *Cholic acid*, and *Tauro-alpha-muricholic acid*, and some toxic metabolites such as *Deoxycytidine*, *N-Acetyl-D-Galactosamine*, and *Phytosphingosine*. *Taurochenodesoxycholic acid*, *Hyocholic acid*, *Cholic acid*, *Methacholine*, *N-Acetyl-D-Galactosamine,* and *Muribaculum* were positively correlated. *Deoxycytidine*, *Methacholine,* and *Taurochenodesoxycholic acid* were positively correlated with *Angelakisella* and *Dubosiella*. These results demonstrate that interactions between the gut microbiota, microbial-derived metabolites and the host may play an important role in CRA production and adenoma-carcinoma transformation. SZF may prevent and treat CRAs by improving intestinal flora disorders and their mechanisms and restoring intestinal flora homeostasis. Furthermore, this study suggests that the joint- application of multiple omics is a potential strategy to reveal the mechanism of action of herbal medicines in the treatment of intestinal diseases.

## Data availability statement

The datasets presented in this study can be found in online repositories. The names of the repository/repositories and accession number(s) can be found at: https://www.ncbi.nlm.nih.gov/, PRJNA860747.

## Ethics statement

The animal study was reviewed and approved by he Laboratory animal welfare and Ethics Management Committee of Jiangsu Hospital of integrated traditional Chinese and Western Medicine.

## Author contributions

JS: performed laboratory work and wrote first draft. HG: developed the concept, managed samples collection, and supervised laboratory work and data analysis. ZL and LW: performed revision and contributed to writing manuscript. ZY: performed laboratory work. JL and YH: managed samples collection. YT and LL: supervised, reviewed, and edited the manuscript. All authors contributed to the article and approved the submitted version.

## Funding

This research was funded by the Project of National Clinical Research Base of Traditional Chinese Medicine in Jiangsu Province (JD2019SZXZD02 and JD2019SZXZD03) and Youth Scientific research Project of Jiangsu Academy of Traditional Chinese Medicine (QNKXYJ202106).

## Conflict of interest

The authors declare that the research was conducted in the absence of any commercial or financial relationships that could be construed as a potential conflict of interest.

## Publisher’s note

All claims expressed in this article are solely those of the authors and do not necessarily represent those of their affiliated organizations, or those of the publisher, the editors and the reviewers. Any product that may be evaluated in this article, or claim that may be made by its manufacturer, is not guaranteed or endorsed by the publisher.

## Footnotes

^1^ http://www.metaboanalyst.ca/

^2^ https://www.genome.jp/KE-GG/

## Abbreviations

SZF: Sanzi formula
CRA: colorectal adenoma
OTUs: operational taxonomic units
FMT: Fecal microbiota transplantation
LC-ESI-MS/MS: Liquid chromatography-electrospray ionization-Tandem mass spectrometry
QQQ: triple quadrupole
QTRAP: triple quadrupole-linear ion trap mass spectrometer
ANOVA: analysis of variance
PLS-DA: the partial least squares discrimination analysis
VIP value: the variable importance of projection value
NMDS: non-metric multidimensional scaling
LEfSe: the linear discriminant analysis effect size
MWDB: Metware database
TCM: Traditional Chinese Medicine
IPA: indolepropionic acid
WTM: Widely Targeted Metabolome
FXR: Farnesoid X receptor
TNFα: tumor necrosis factor α
IL-1β: interleukin 1β
γ-IFN: interferon γ
IL-17A: Interleukin 17A
CD4: cluster of differentiation 4
