# Supplementary figures and images for: Exploring the mechanism of action of Sanzi formula in intervening colorectal adenoma by targeting intestinal flora and intestinal metabolism
Source: Front Microbiol. 2022 Sep 8;13:1001372. doi: 10.3389/fmicb.2022.1001372 (PMC9504867; doi:10.3389/fmicb.2022.1001372)

p-value =  $1.9\text{e-}05$ ; n = 30

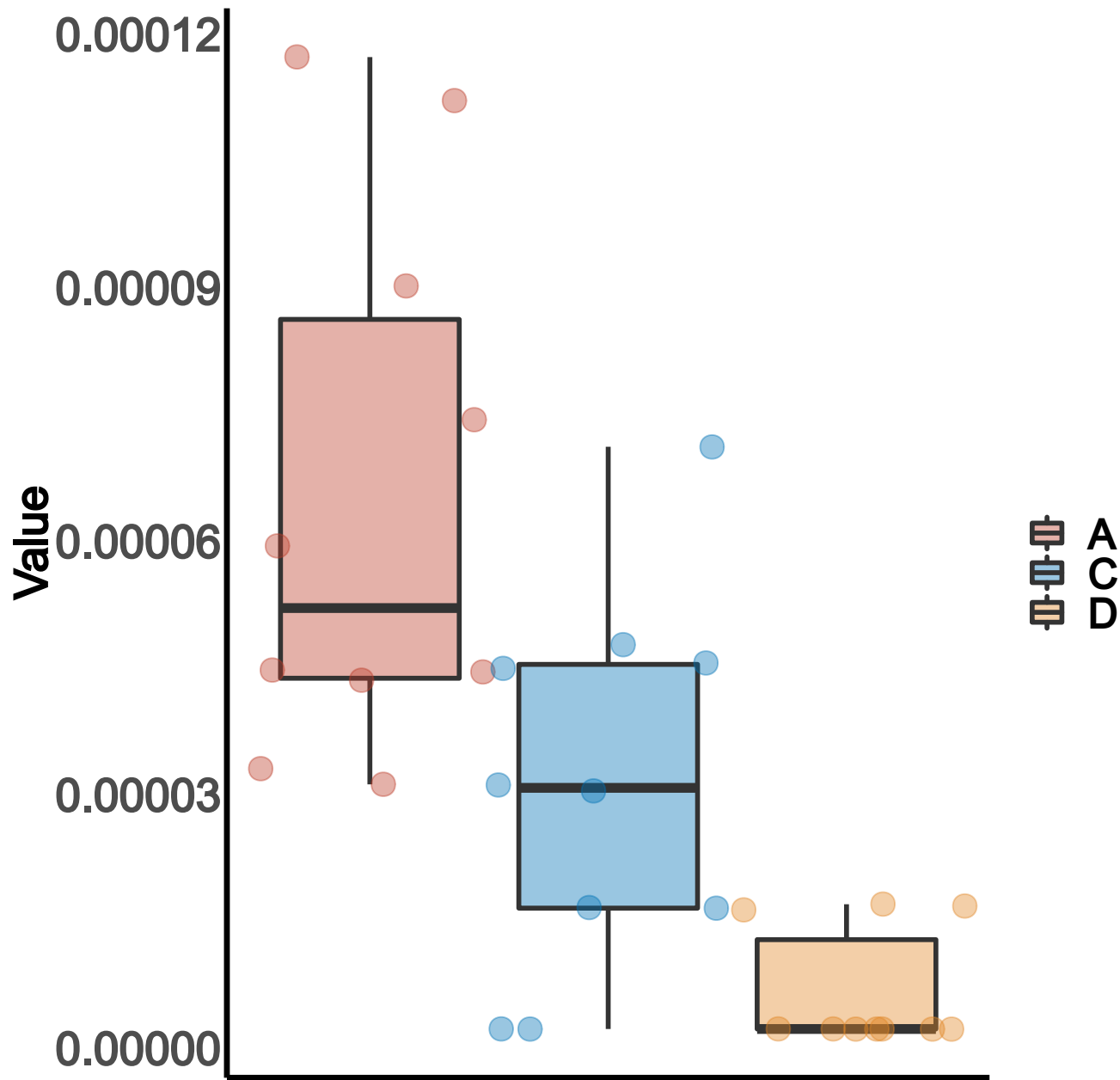

Supplement: Supplementary file 5 [file Data_Sheet_1.ZIP › boxplot/index10_boxplot_ANOVA.pdf]

p-value =  $2.7\text{e-}05$ ; n = 30

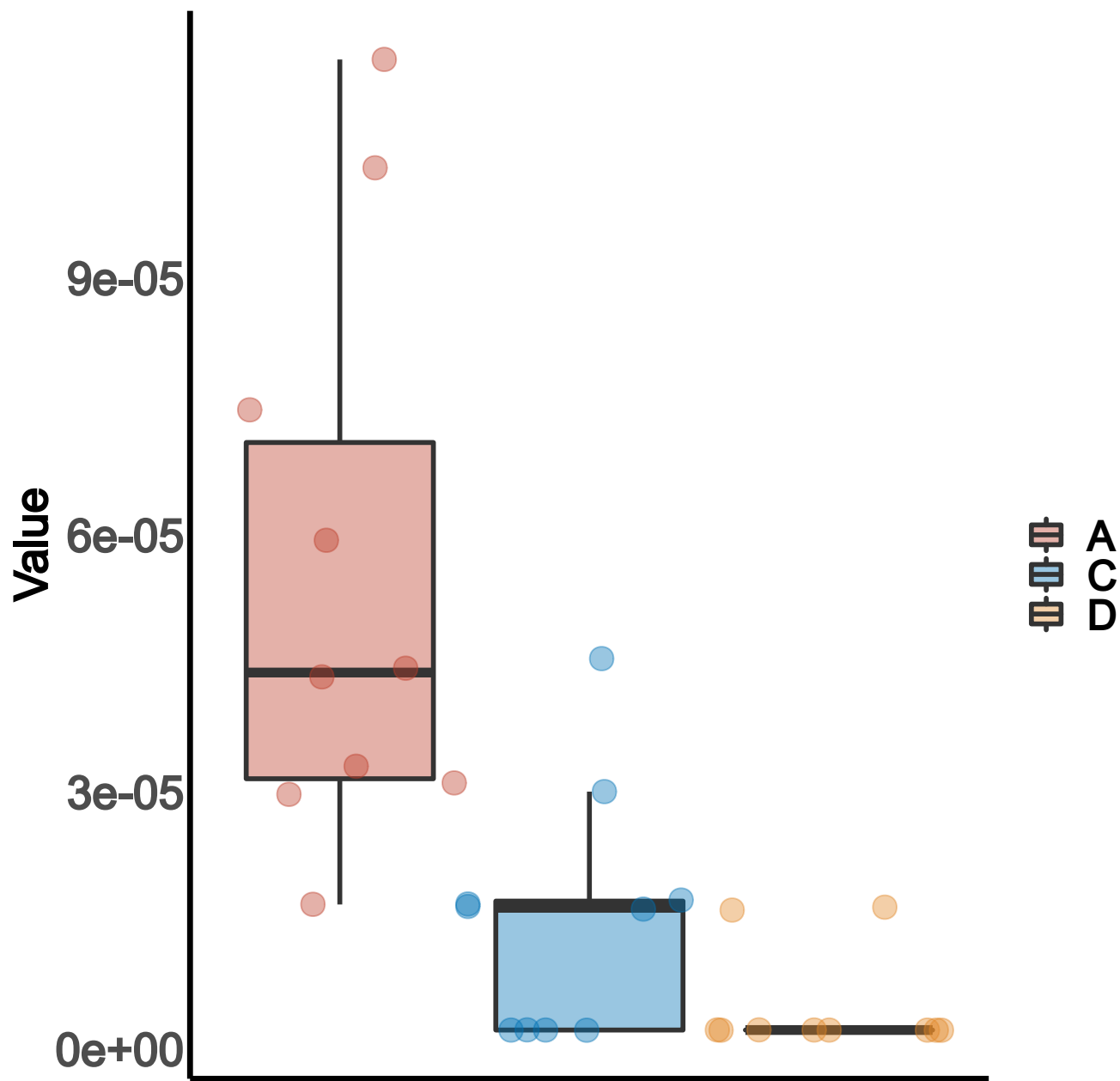

Supplement: Supplementary file 5 [file Data_Sheet_1.ZIP › boxplot/index11_boxplot_ANOVA.pdf]

p-value =  $3e-05$ ; n = 30

Value

$0e+00$

$2e-05$

$4e-05$

A  
C  
D

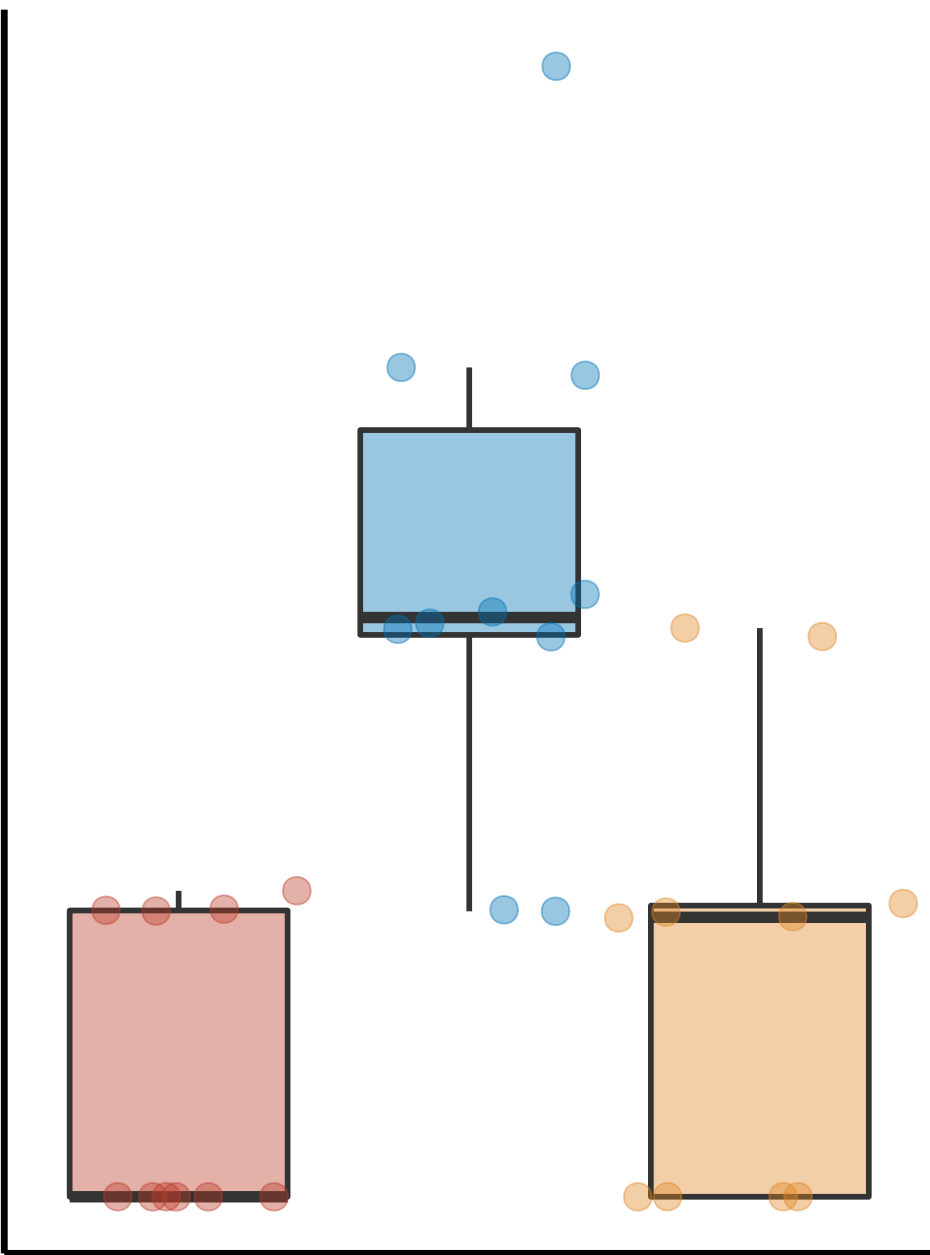

Supplement: Supplementary file 5 [file Data_Sheet_1.ZIP › boxplot/index12_boxplot_ANOVA.pdf]

p-value =  $3.1\text{e-}05$ ; n = 30

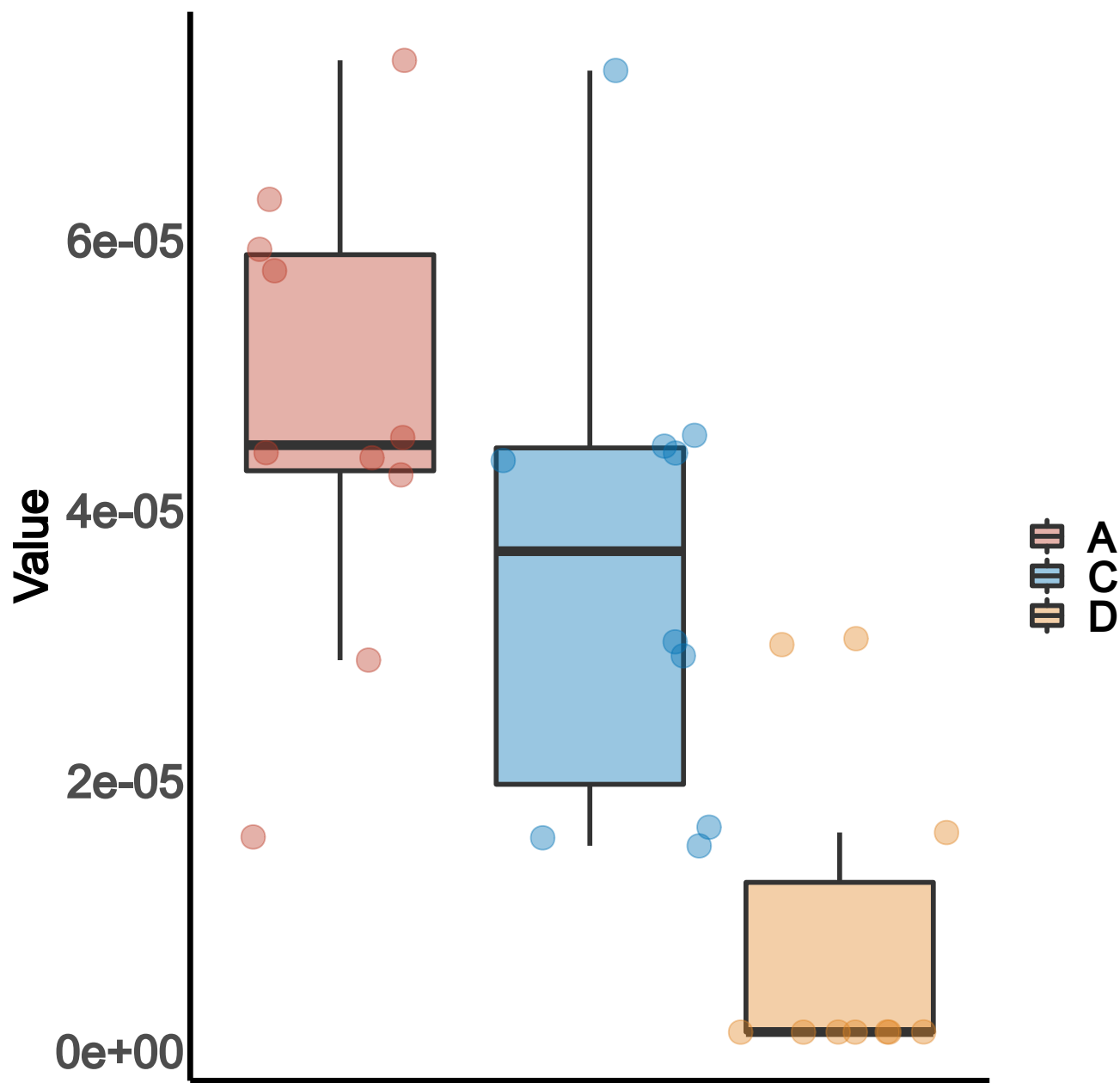

Supplement: Supplementary file 5 [file Data_Sheet_1.ZIP › boxplot/index13_boxplot_ANOVA.pdf]

**p-value =  $3.2\text{e-}05$ ; n = 30**

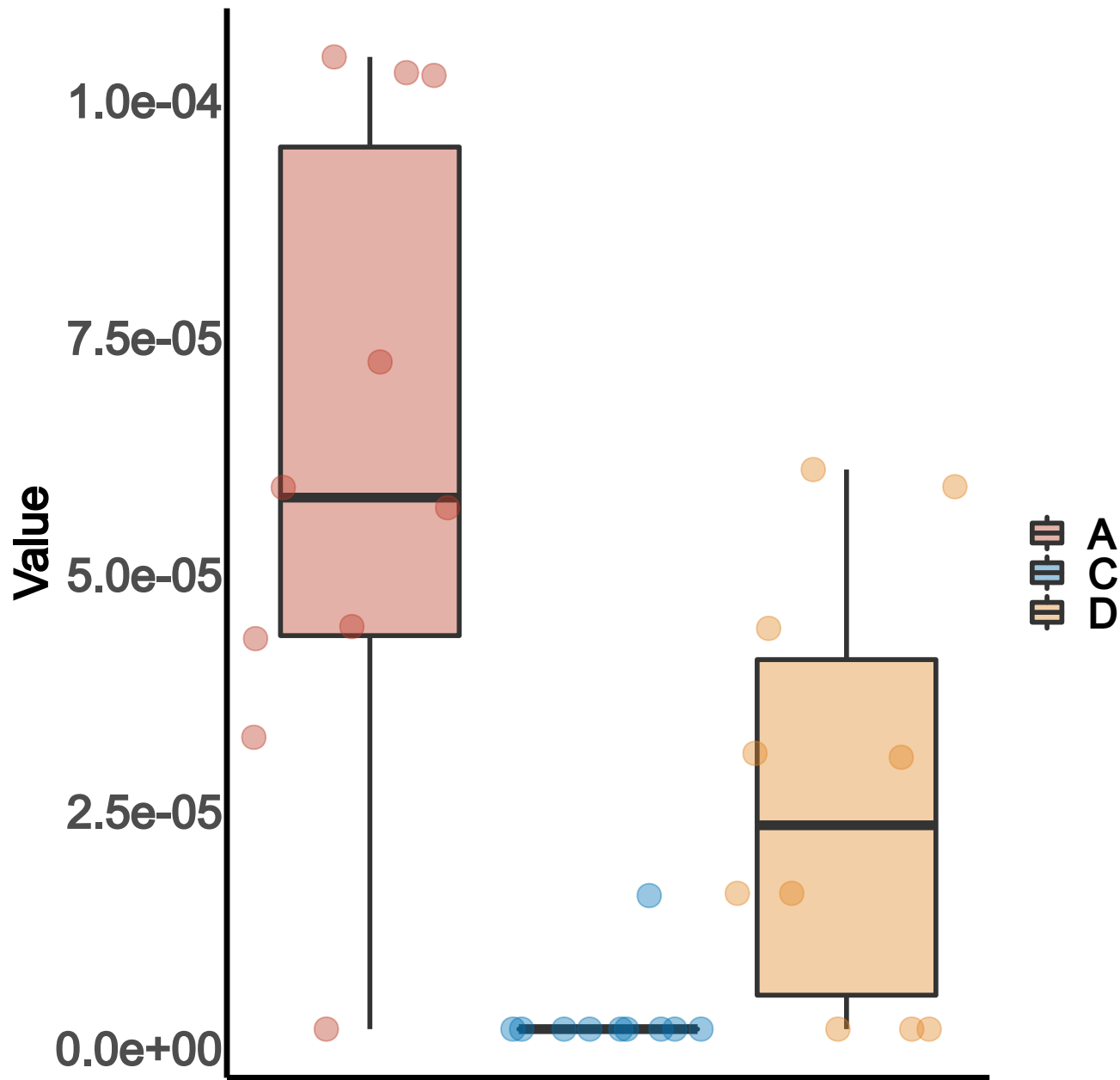

Supplement: Supplementary file 5 [file Data_Sheet_1.ZIP › boxplot/index14_boxplot_ANOVA.pdf]

p-value =  $4.7e-05$ ; n = 30

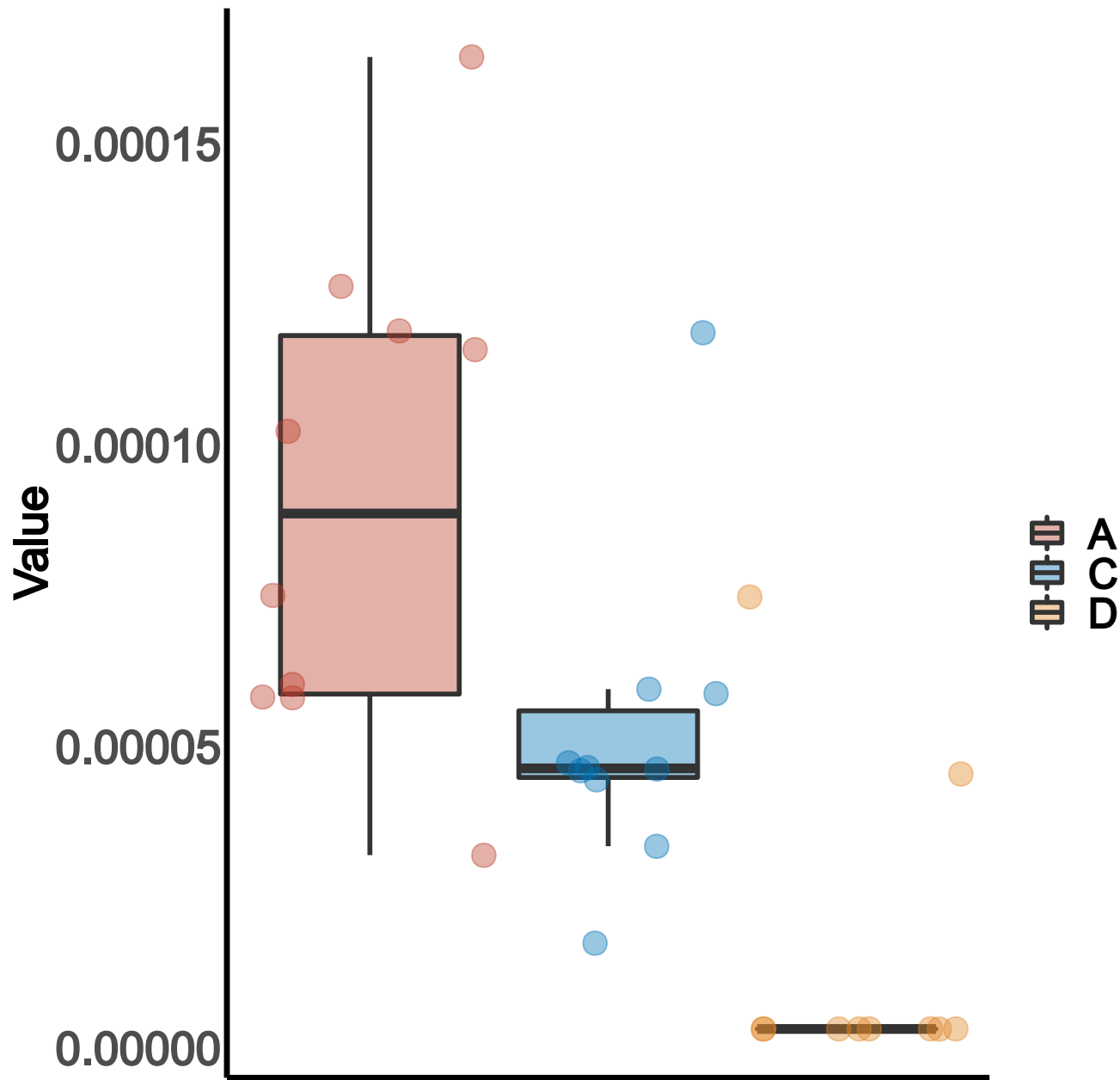

Supplement: Supplementary file 5 [file Data_Sheet_1.ZIP › boxplot/index15_boxplot_ANOVA.pdf]

p-value =  $8.6\text{e-}05$ ; n = 30

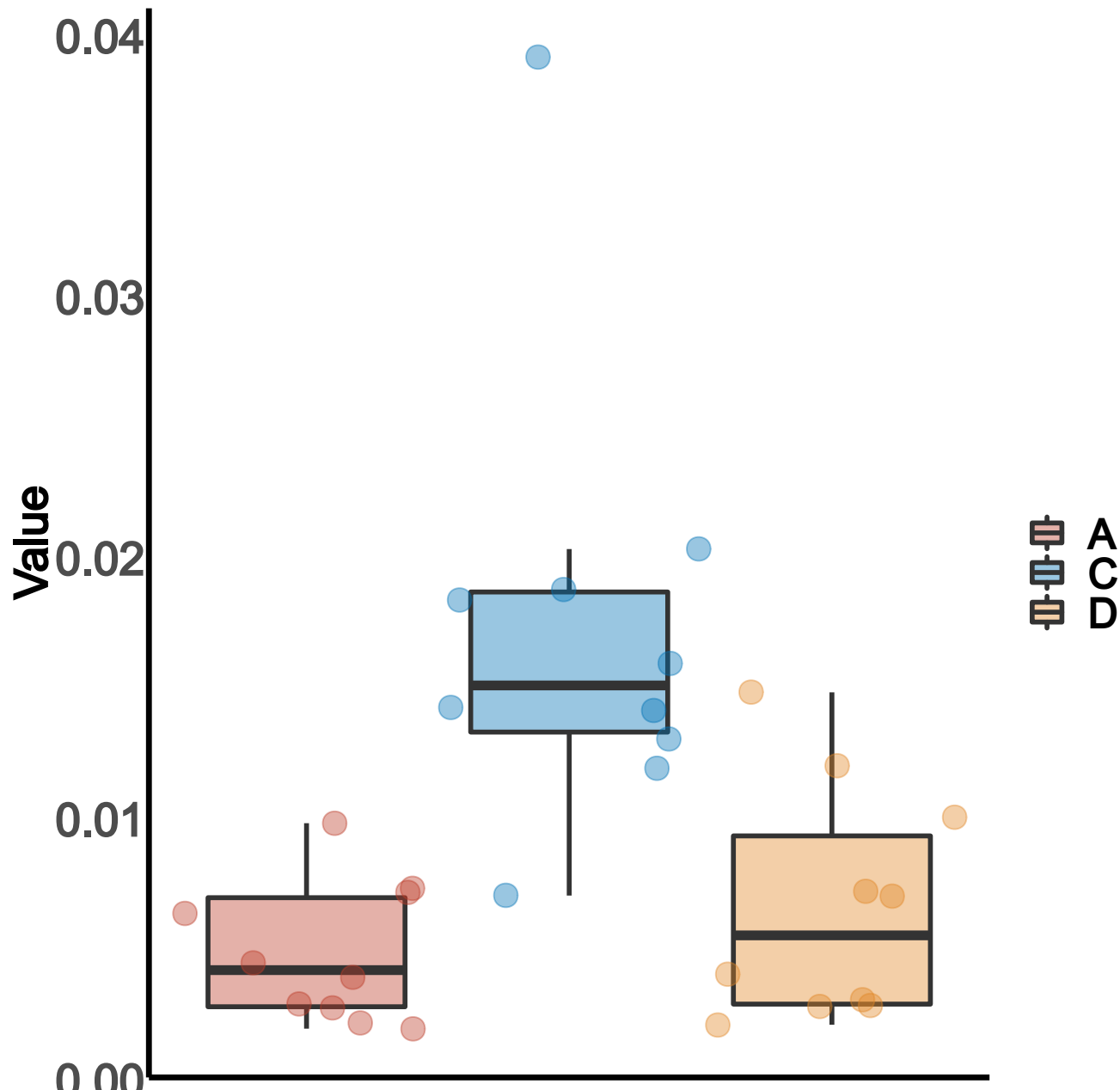

Supplement: Supplementary file 5 [file Data_Sheet_1.ZIP › boxplot/index16_boxplot_ANOVA.pdf]

p-value = 0.00012; n = 30

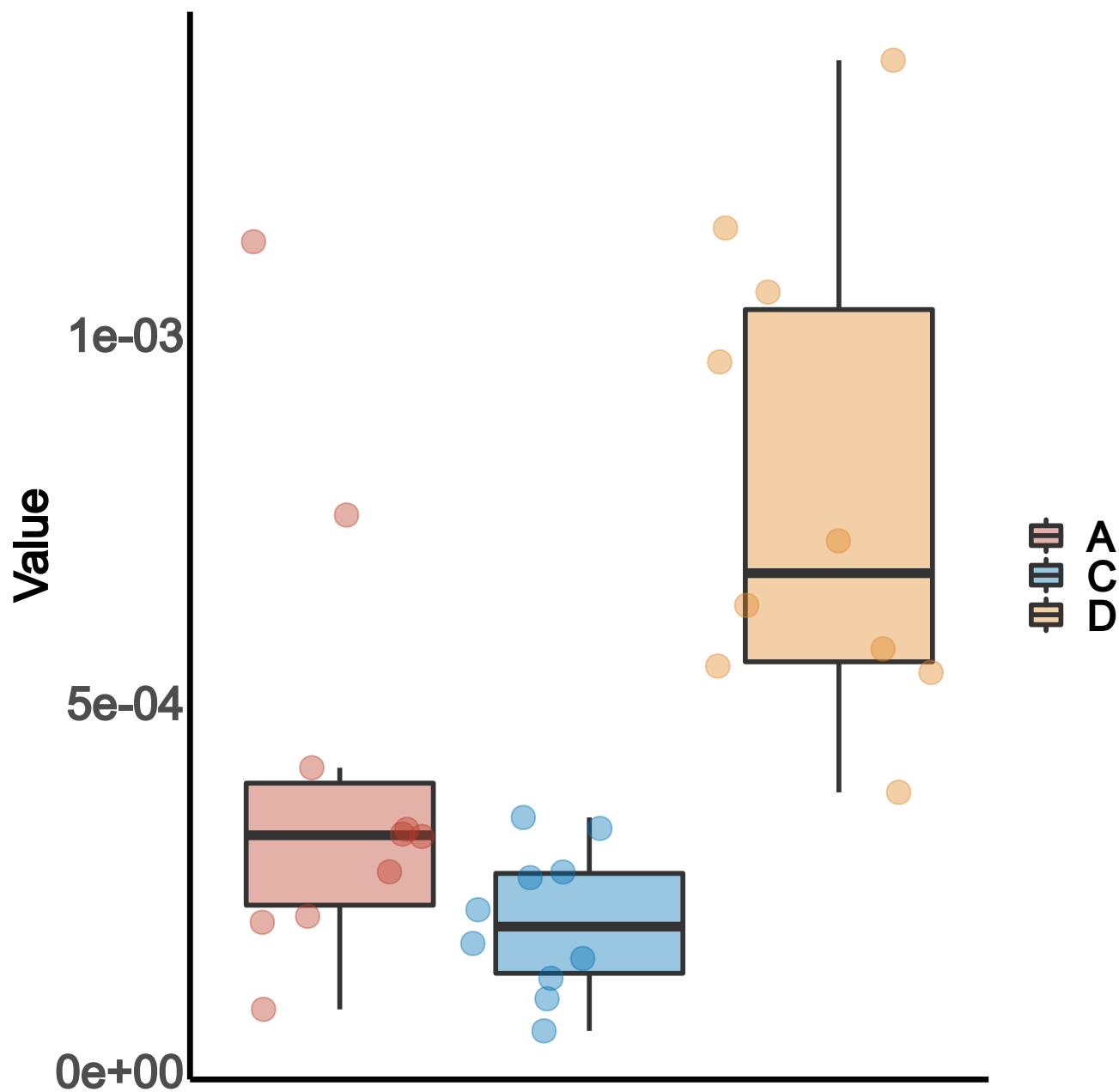

Supplement: Supplementary file 5 [file Data_Sheet_1.ZIP › boxplot/index18_boxplot_ANOVA.pdf]

**p-value = 0.00015; n = 30**

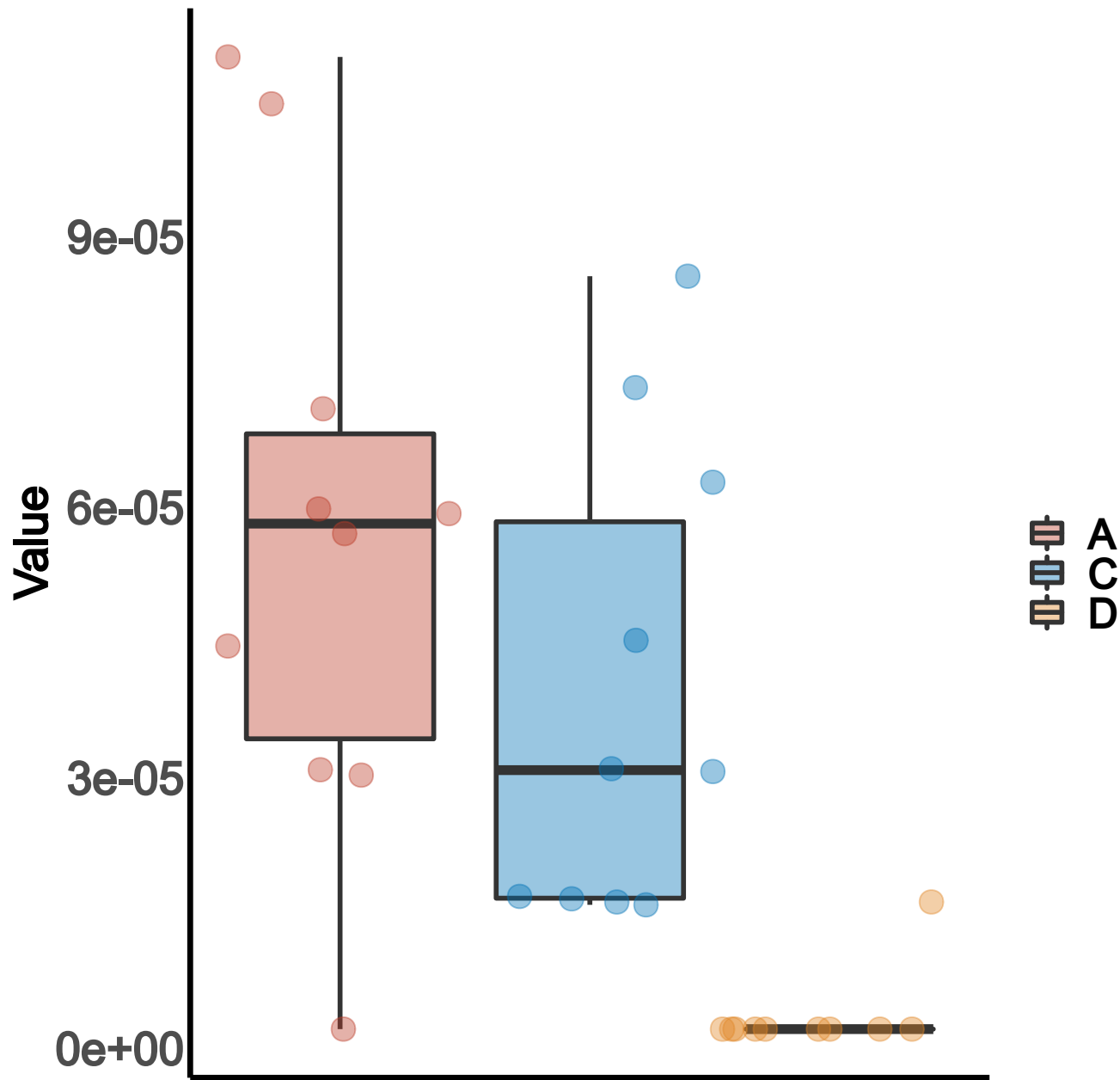

Supplement: Supplementary file 5 [file Data_Sheet_1.ZIP › boxplot/index19_boxplot_ANOVA.pdf]

**p-value = 5.3e-09; n = 30**

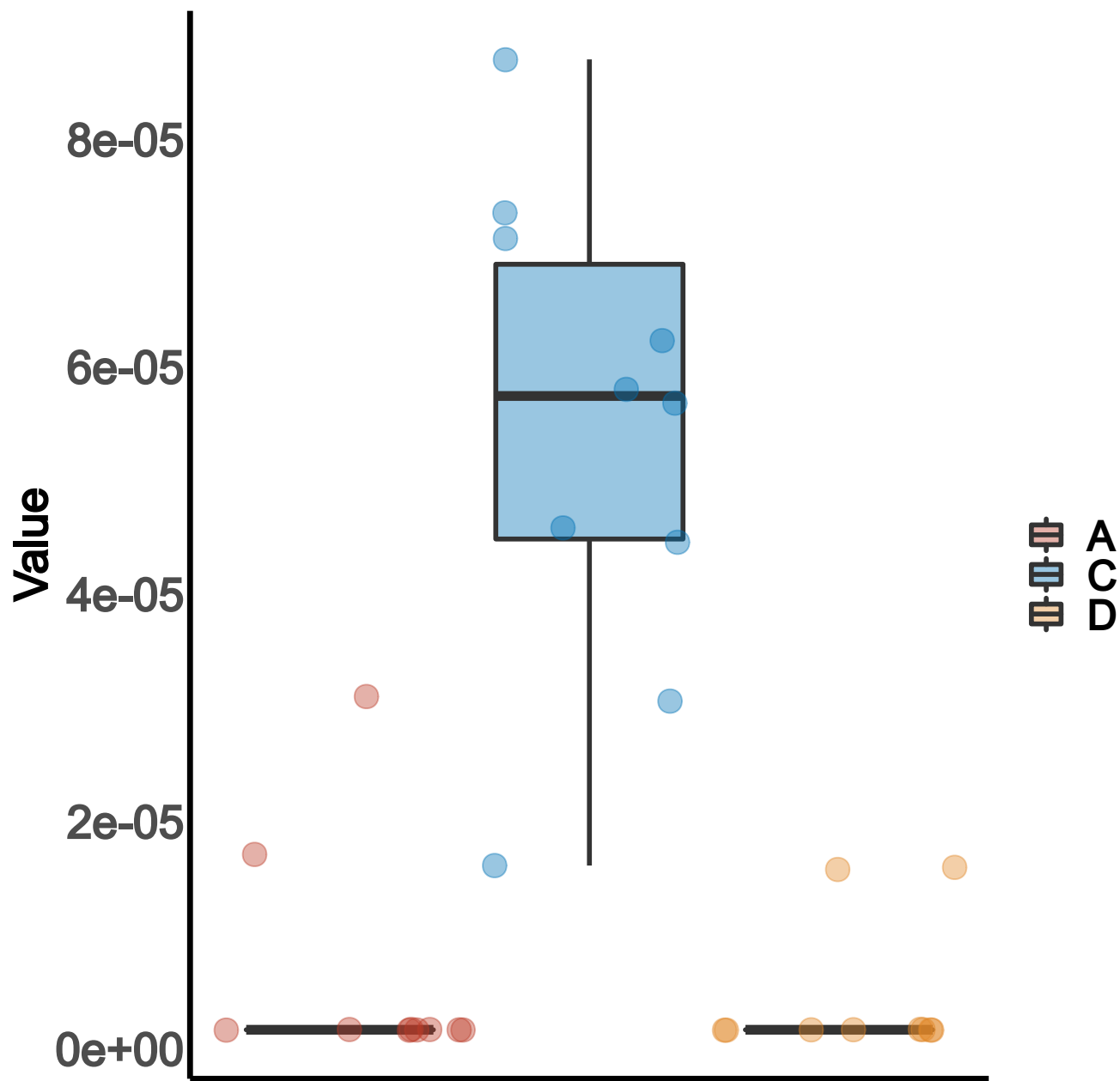

Supplement: Supplementary file 5 [file Data_Sheet_1.ZIP › boxplot/index1_boxplot_ANOVA.pdf]

**p-value = 0.00015; n = 30**

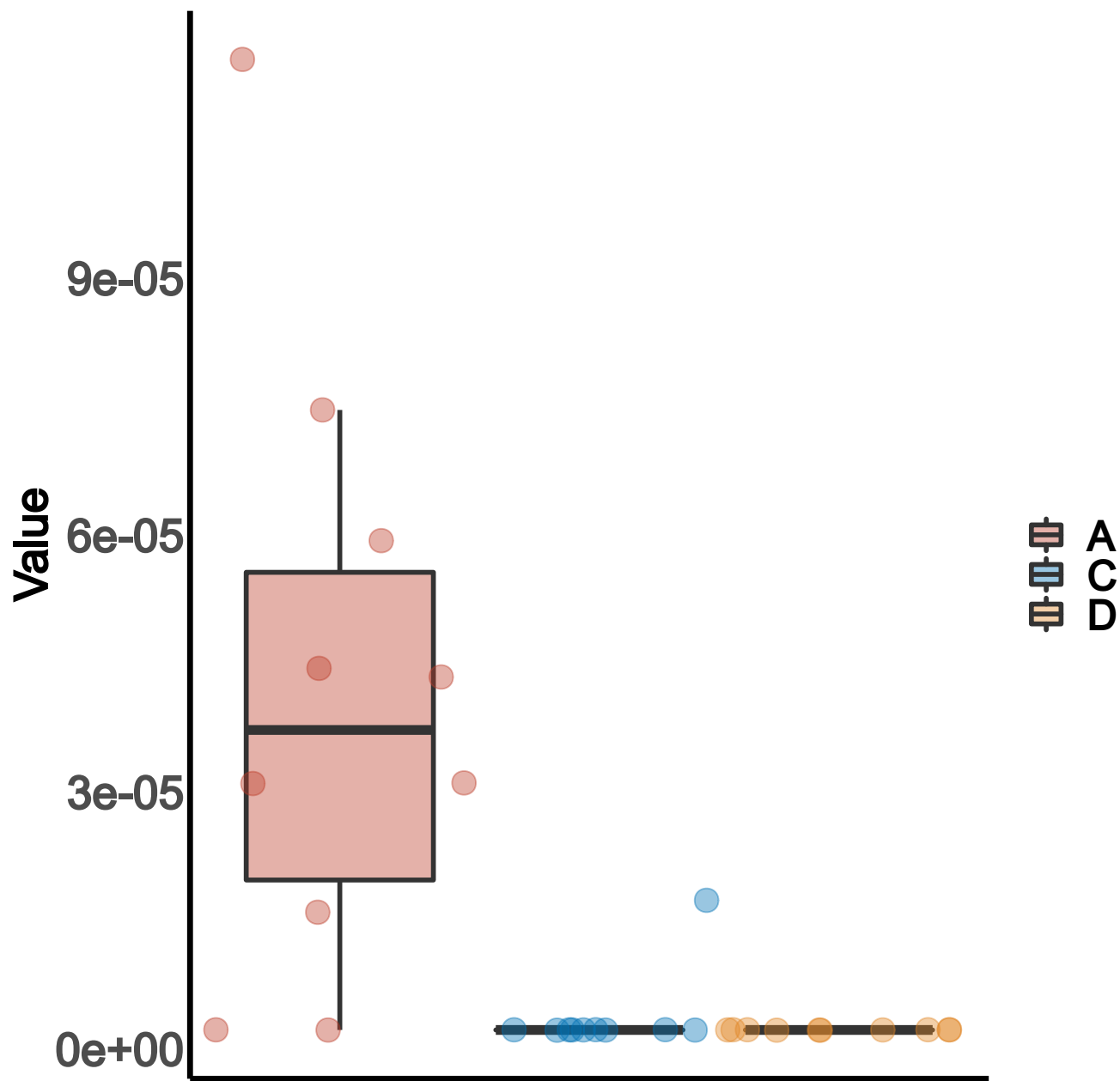

Supplement: Supplementary file 5 [file Data_Sheet_1.ZIP › boxplot/index20_boxplot_ANOVA.pdf]

**p-value = 0.00025; n = 30**

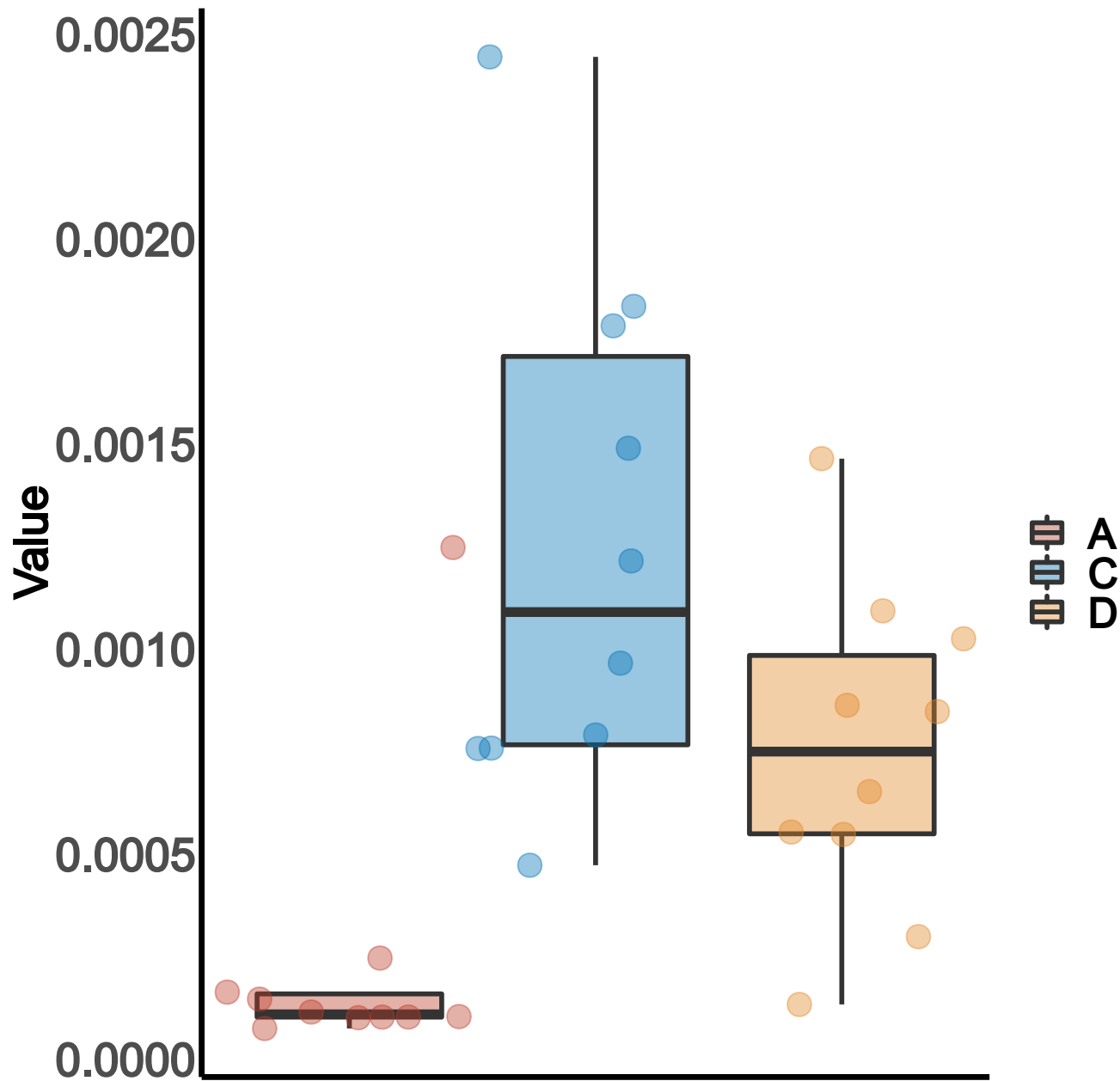

Supplement: Supplementary file 5 [file Data_Sheet_1.ZIP › boxplot/index21_boxplot_ANOVA.pdf]

**p-value = 0.00039; n = 30**

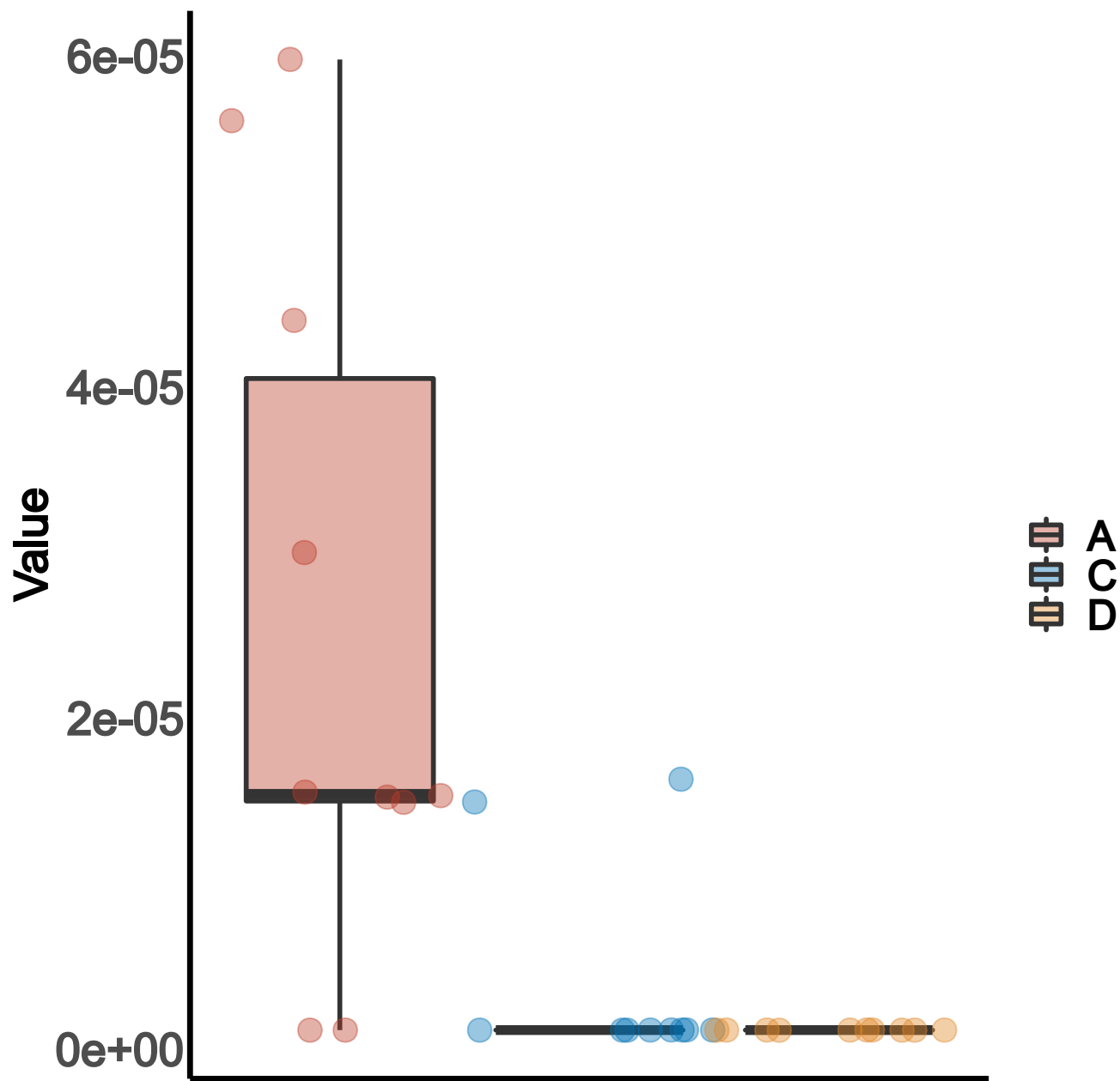

Supplement: Supplementary file 5 [file Data_Sheet_1.ZIP › boxplot/index22_boxplot_ANOVA.pdf]

**p-value = 0.00073; n = 30**

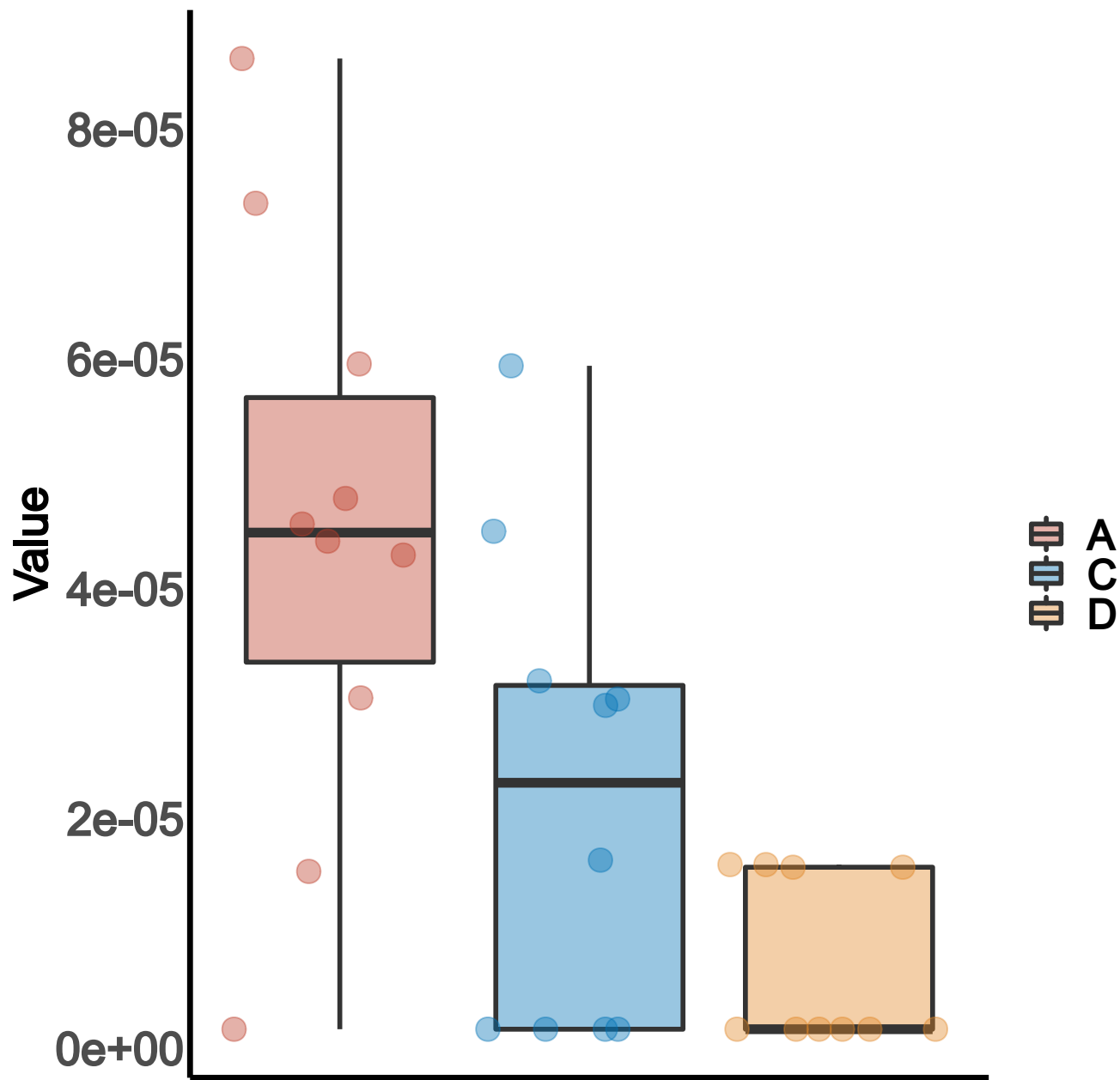

Supplement: Supplementary file 5 [file Data_Sheet_1.ZIP › boxplot/index23_boxplot_ANOVA.pdf]

p-value = 0.00078; n = 30

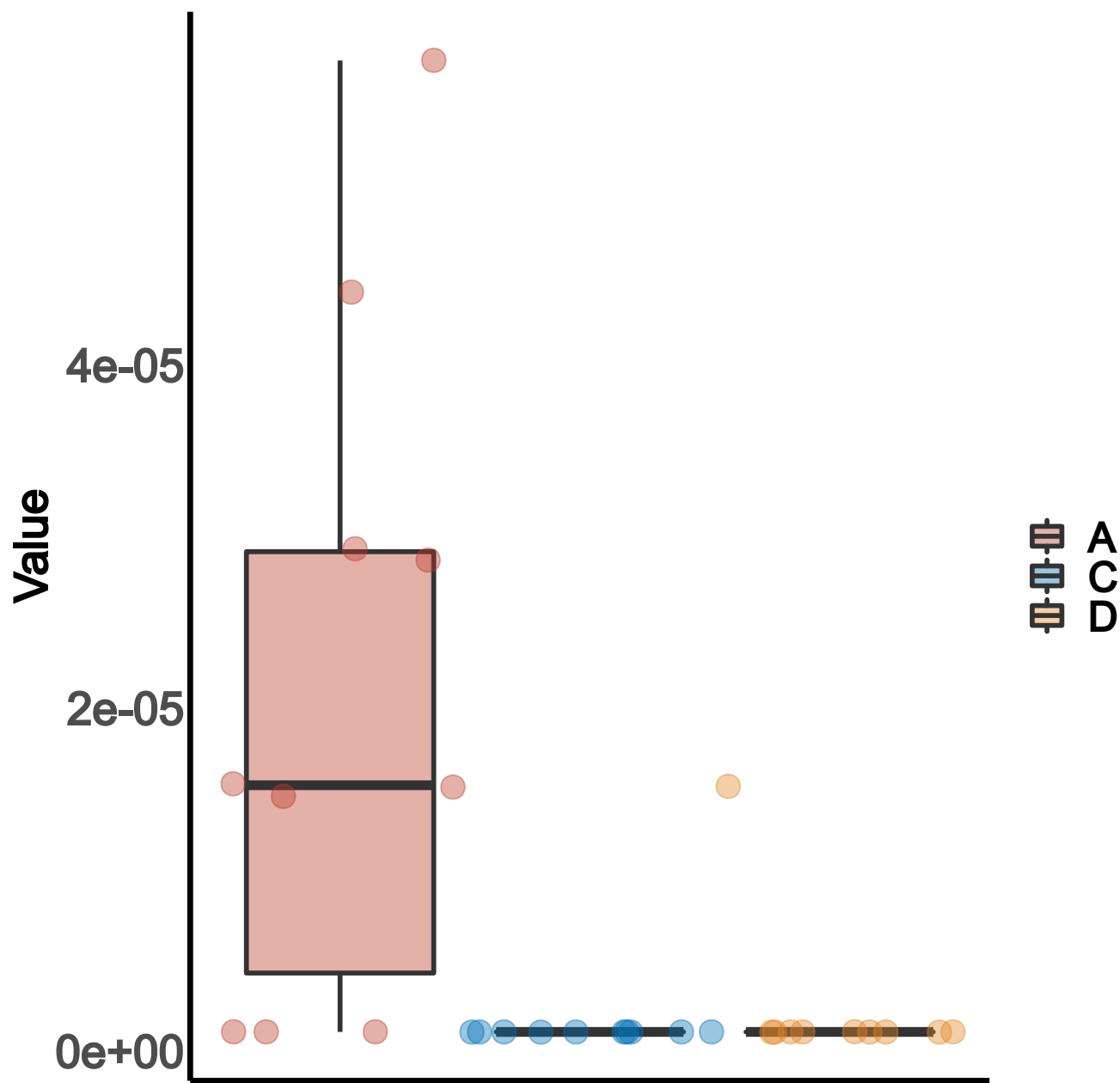

Supplement: Supplementary file 5 [file Data_Sheet_1.ZIP › boxplot/index24_boxplot_ANOVA.pdf]

**p-value = 0.00081; n = 30**

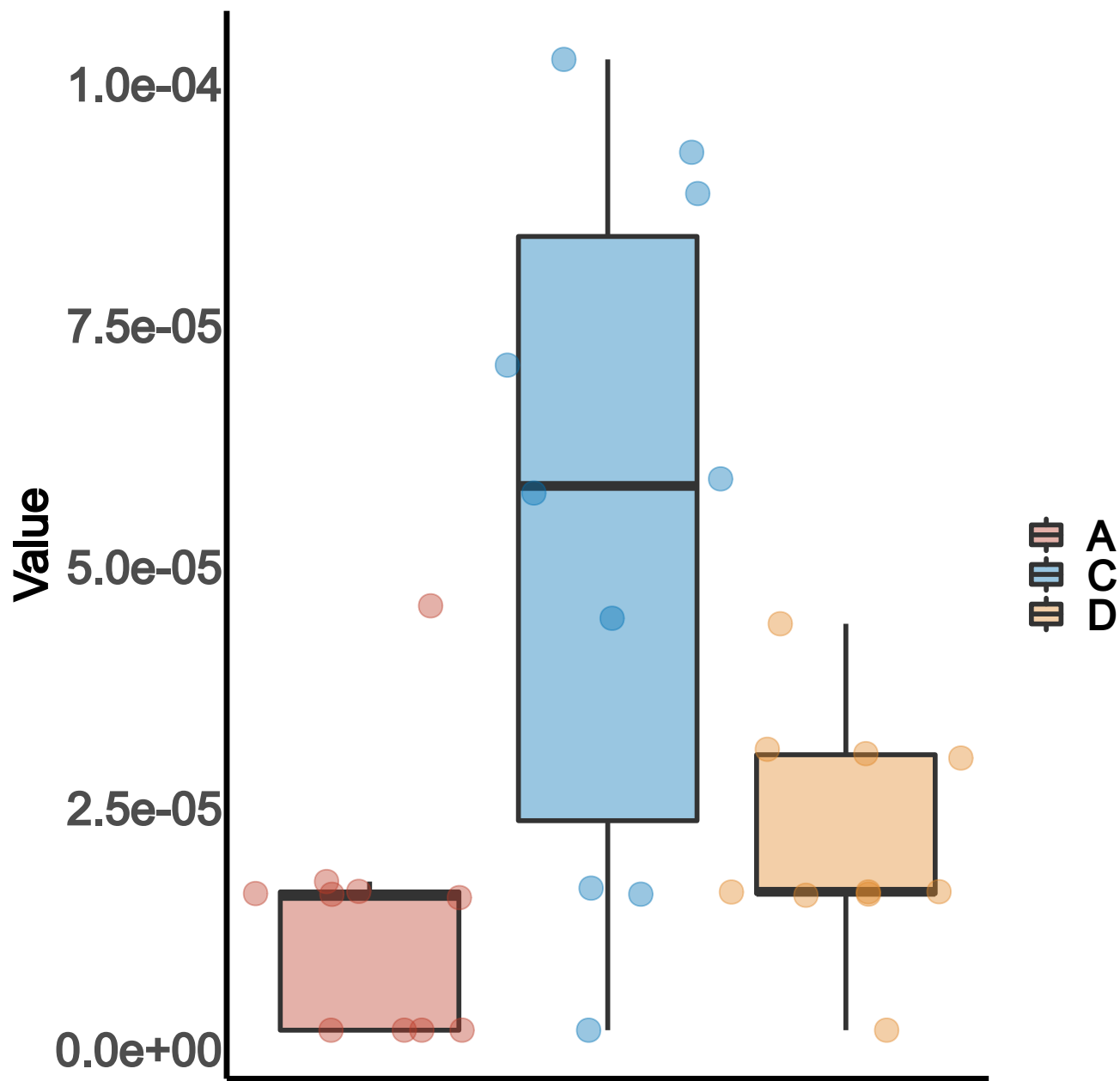

Supplement: Supplementary file 5 [file Data_Sheet_1.ZIP › boxplot/index25_boxplot_ANOVA.pdf]

**p-value = 0.00084; n = 30**

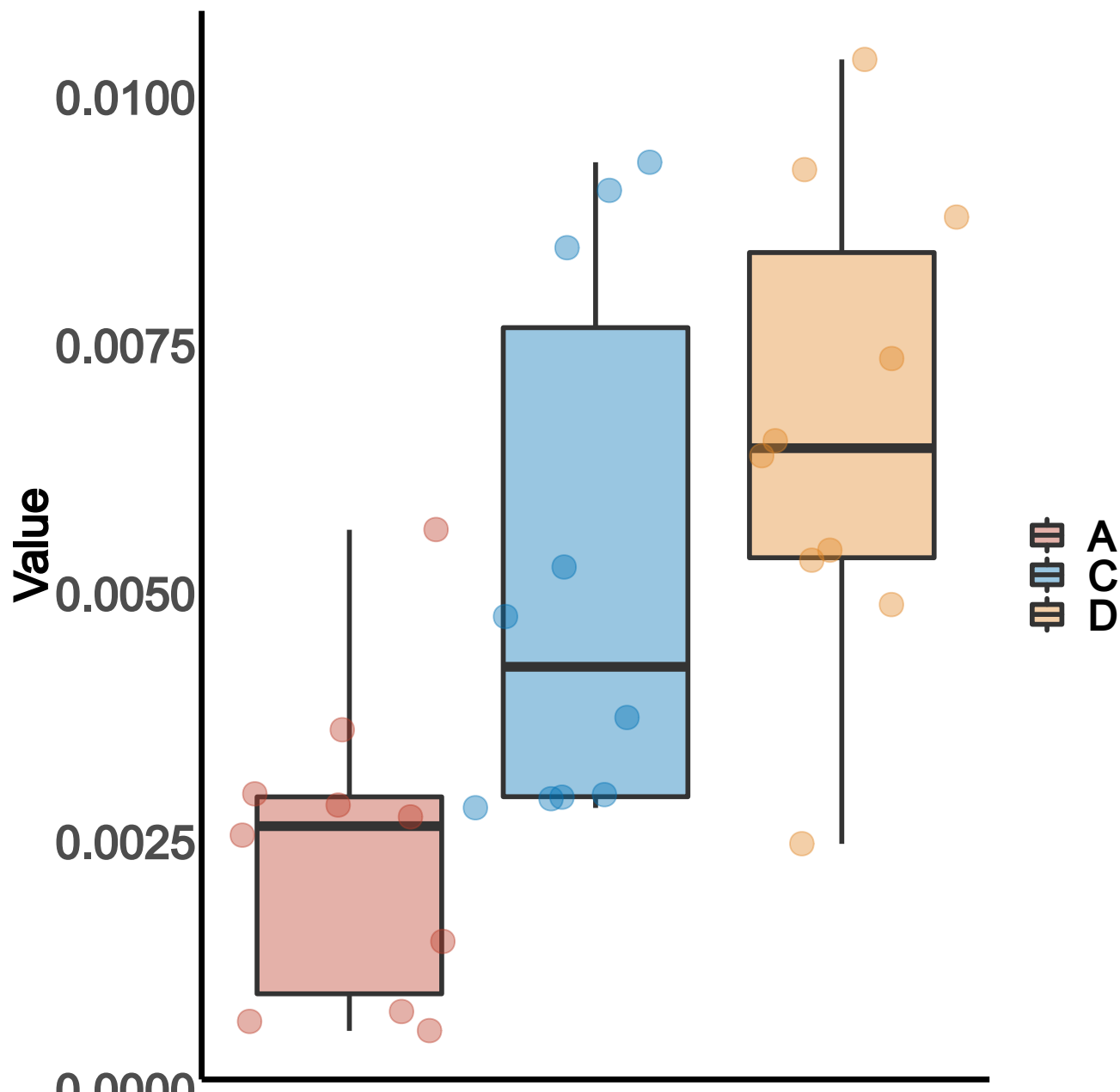

Supplement: Supplementary file 5 [file Data_Sheet_1.ZIP › boxplot/index26_boxplot_ANOVA.pdf]

**p-value = 0.0011; n = 30**

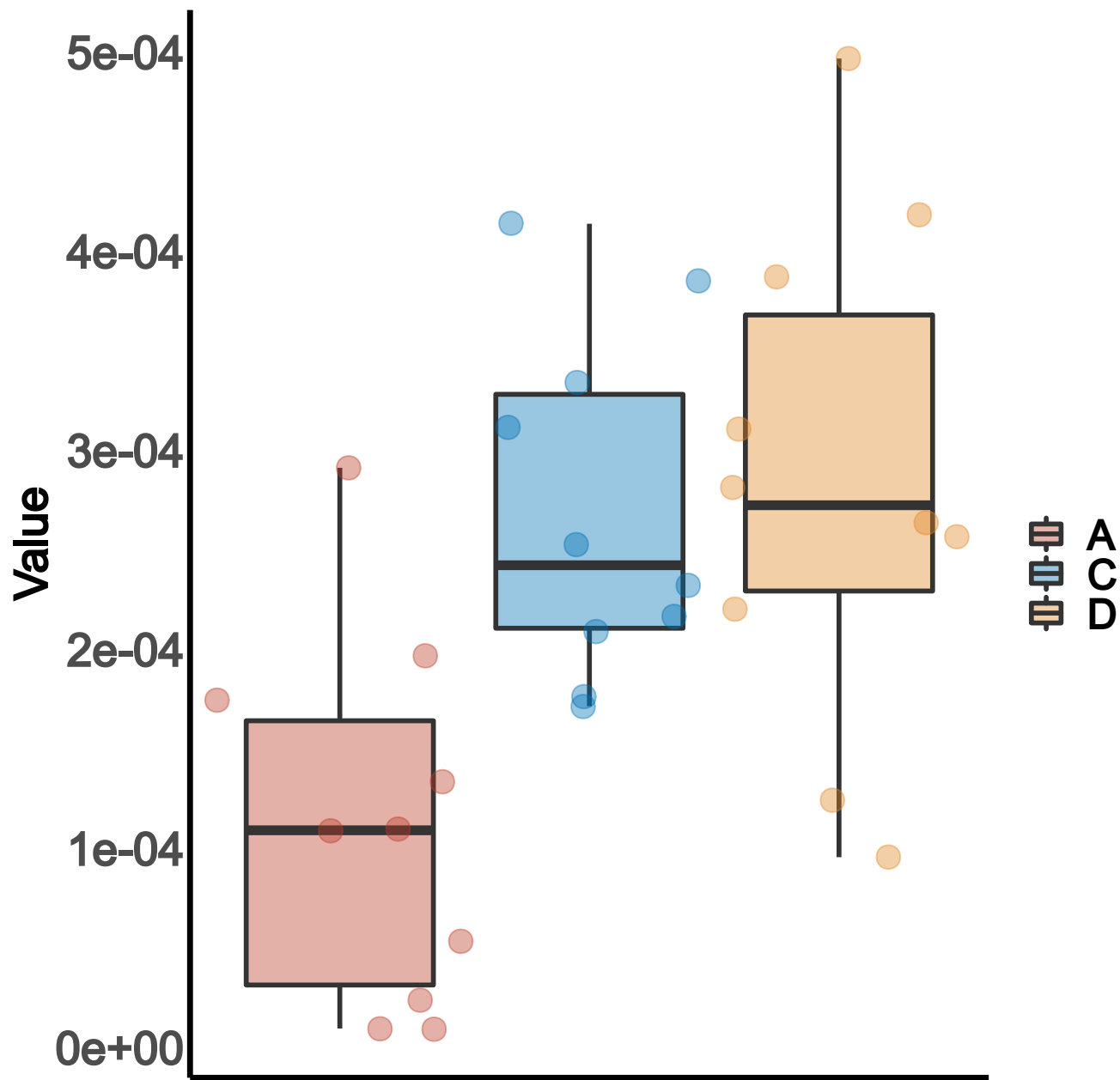

Supplement: Supplementary file 5 [file Data_Sheet_1.ZIP › boxplot/index28_boxplot_ANOVA.pdf]

**p-value = 0.0018; n = 30**

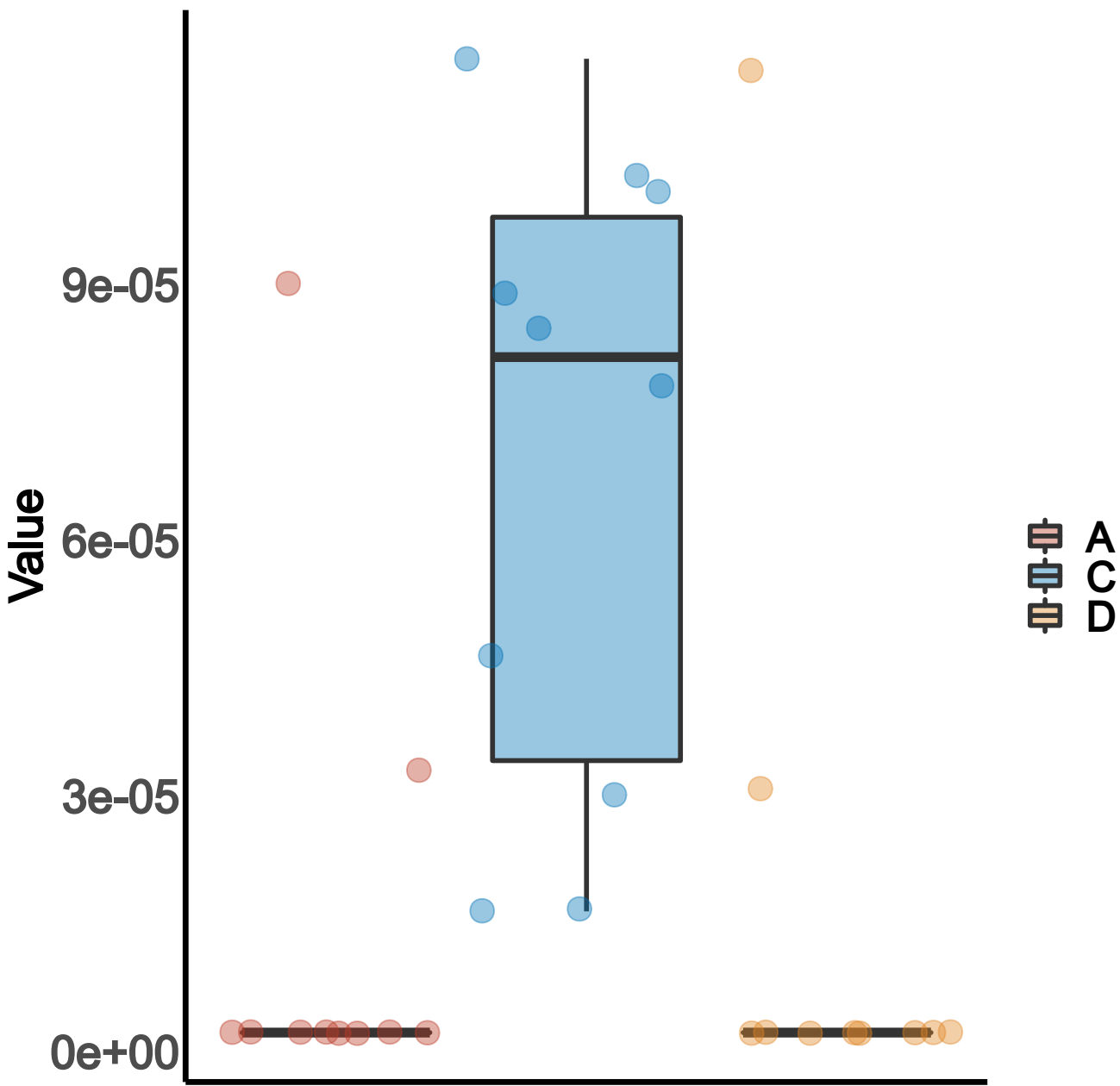

Supplement: Supplementary file 5 [file Data_Sheet_1.ZIP › boxplot/index29_boxplot_ANOVA.pdf]

p-value =  $1.7\text{e-}08$ ; n = 30

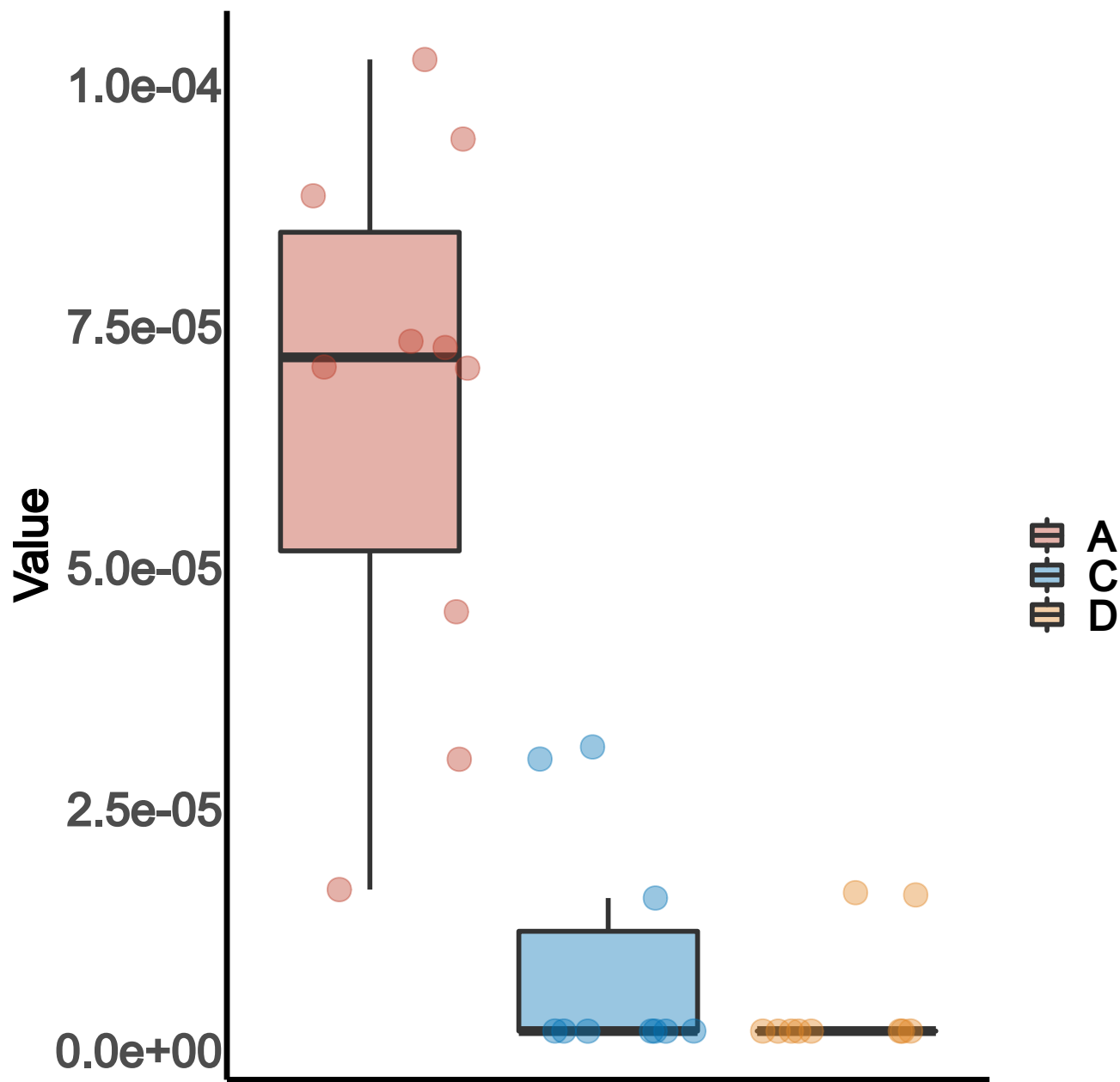

Supplement: Supplementary file 5 [file Data_Sheet_1.ZIP › boxplot/index2_boxplot_ANOVA.pdf]

**p-value = 0.0036; n = 30**

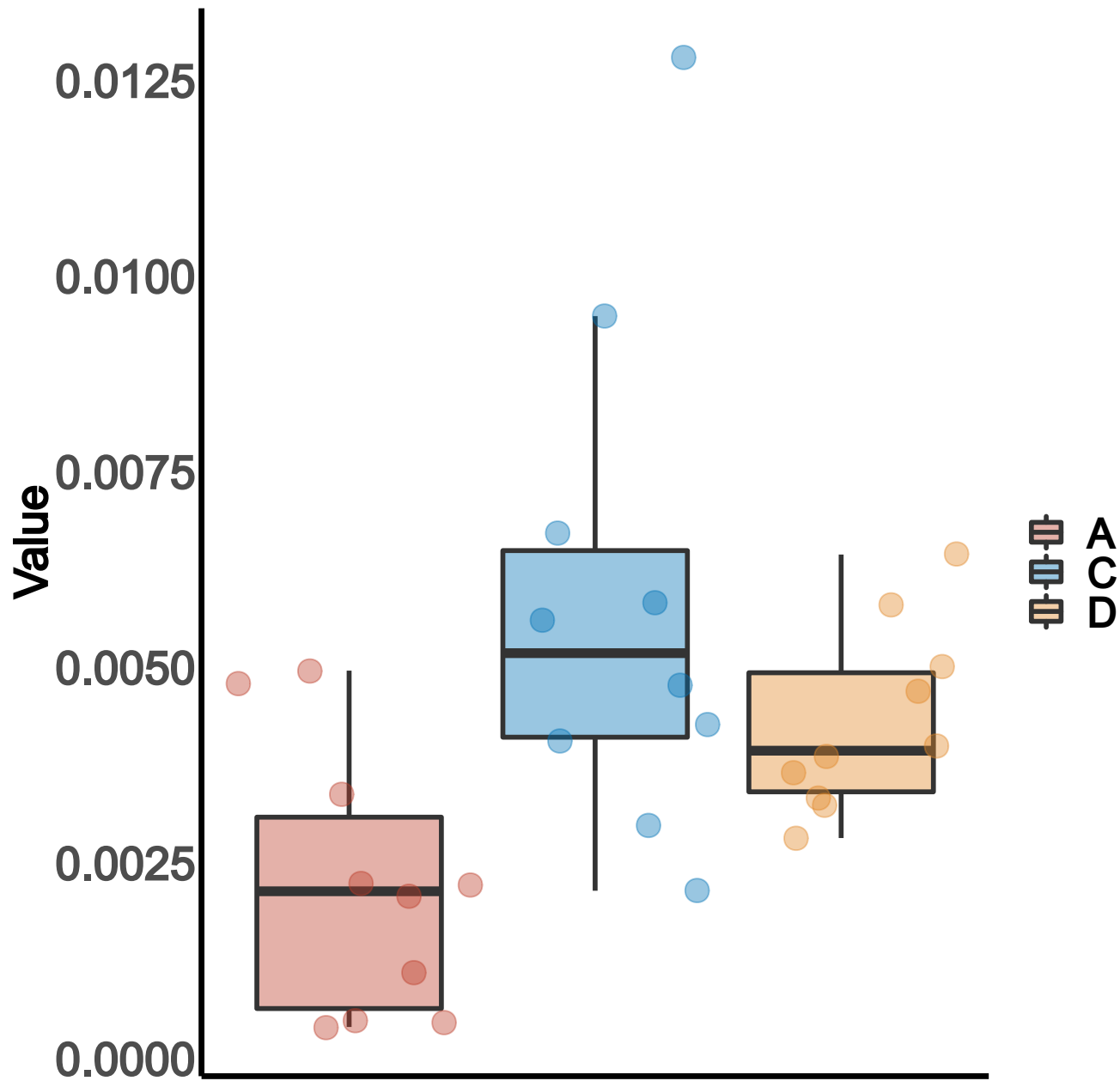

Supplement: Supplementary file 5 [file Data_Sheet_1.ZIP › boxplot/index32_boxplot_ANOVA.pdf]

p-value = 0.0038; n = 30

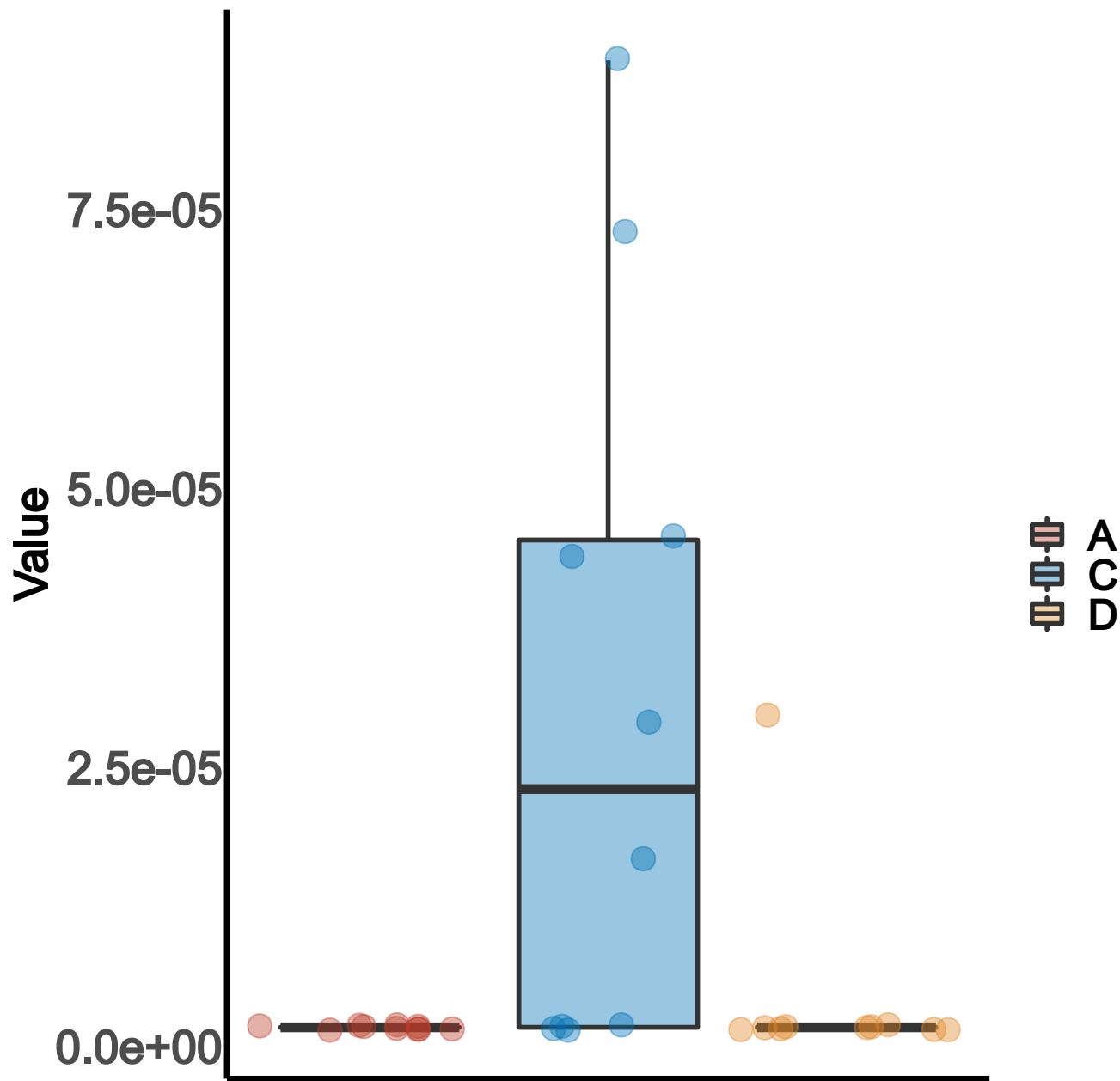

Supplement: Supplementary file 5 [file Data_Sheet_1.ZIP › boxplot/index33_boxplot_ANOVA.pdf]

**p-value = 0.0049; n = 30**

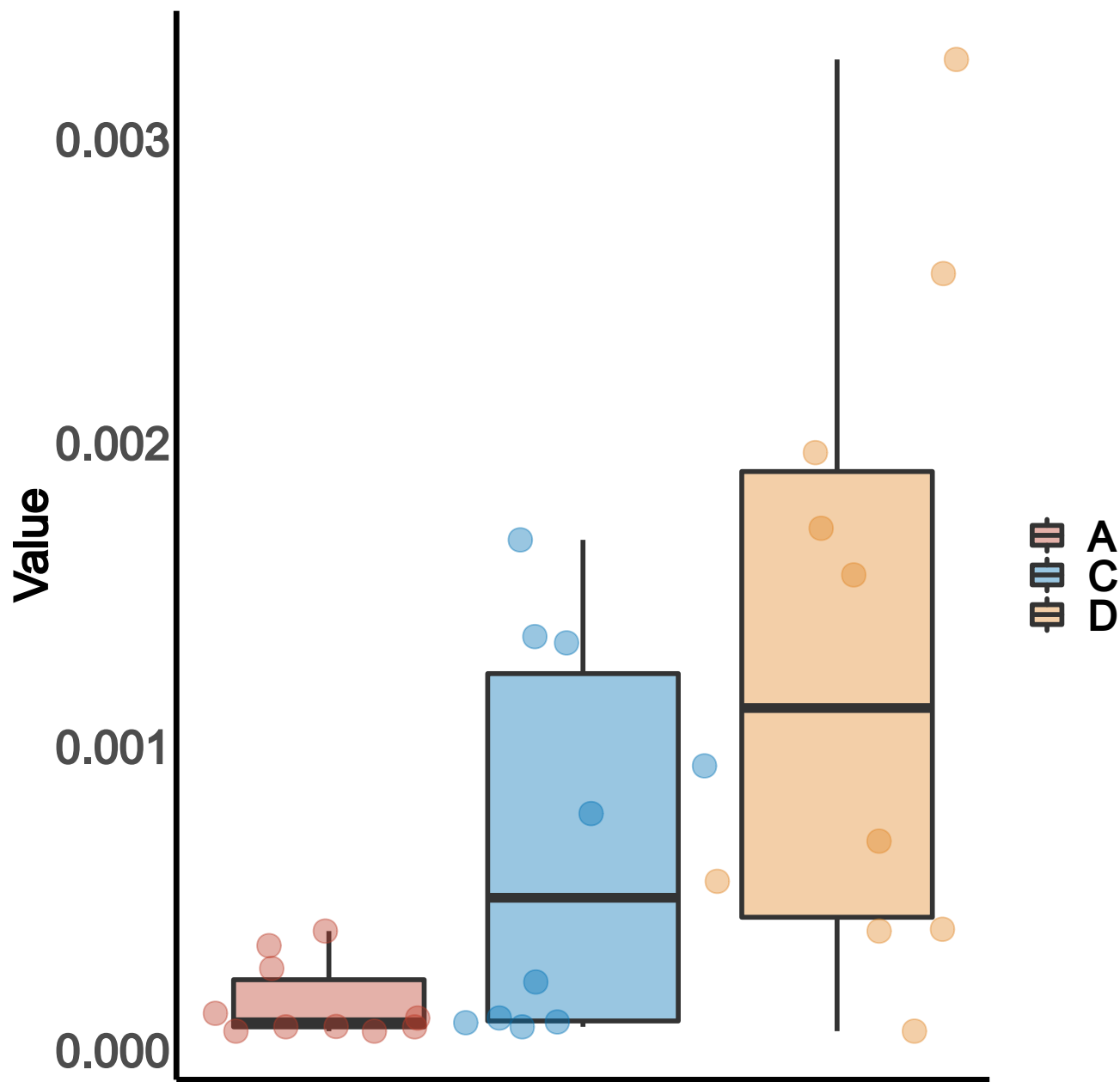

Supplement: Supplementary file 5 [file Data_Sheet_1.ZIP › boxplot/index35_boxplot_ANOVA.pdf]

p-value = 0.005; n = 30

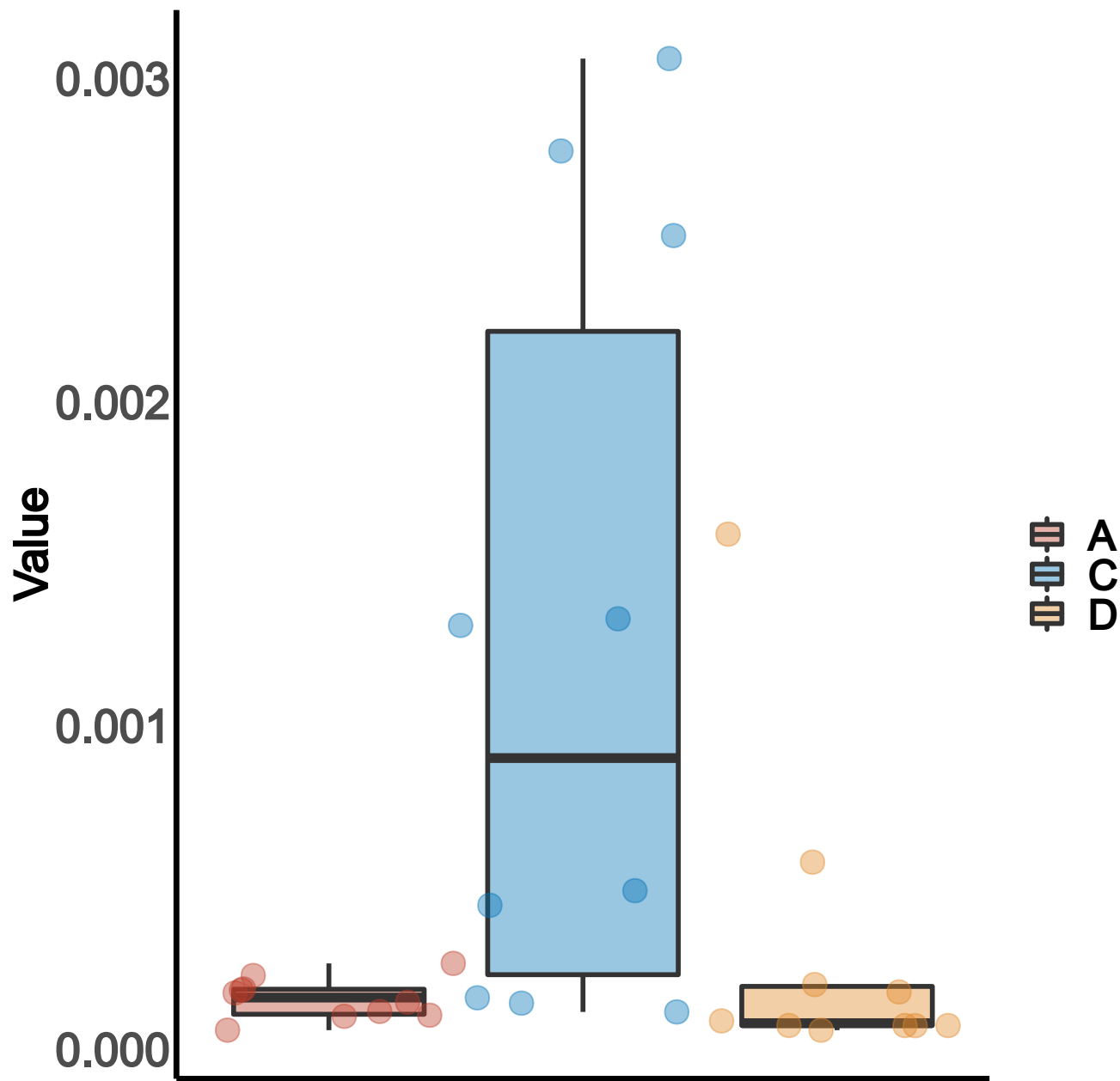

Supplement: Supplementary file 5 [file Data_Sheet_1.ZIP › boxplot/index36_boxplot_ANOVA.pdf]

p-value = 0.0052; n = 30

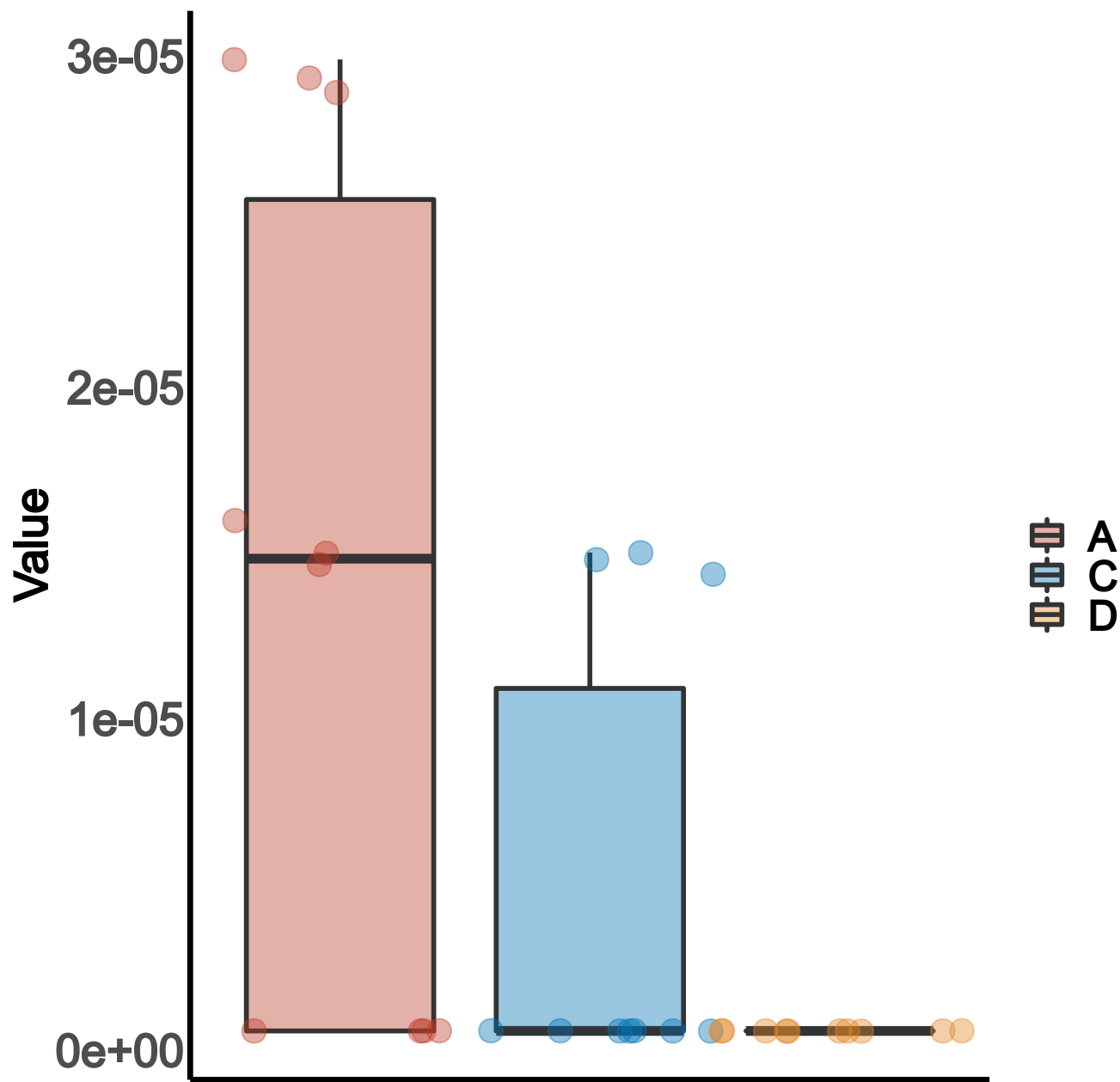

Supplement: Supplementary file 5 [file Data_Sheet_1.ZIP › boxplot/index37_boxplot_ANOVA.pdf]

**p-value = 0.0053; n = 30**

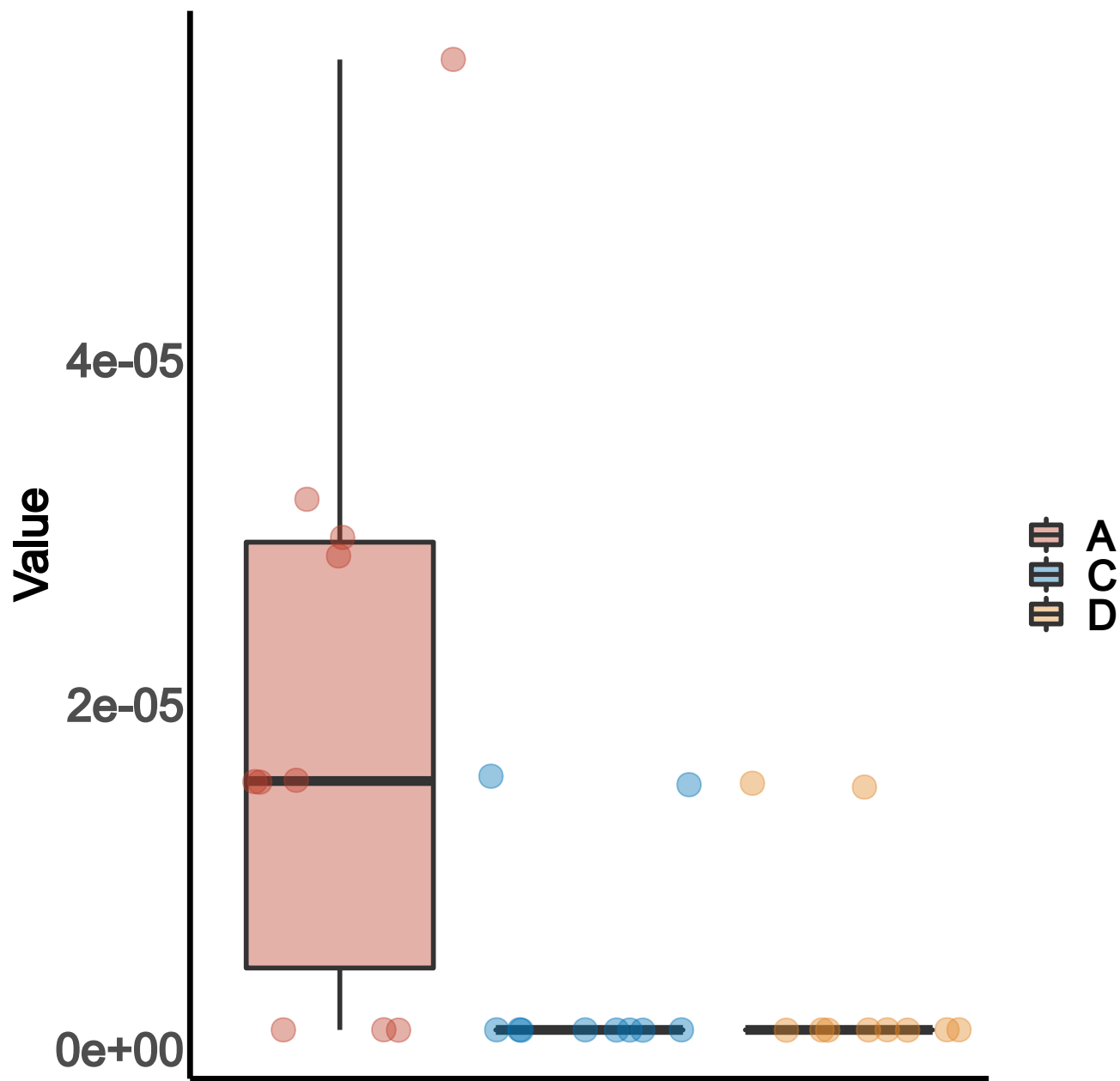

Supplement: Supplementary file 5 [file Data_Sheet_1.ZIP › boxplot/index38_boxplot_ANOVA.pdf]

p-value = 0.0054; n = 30

Value

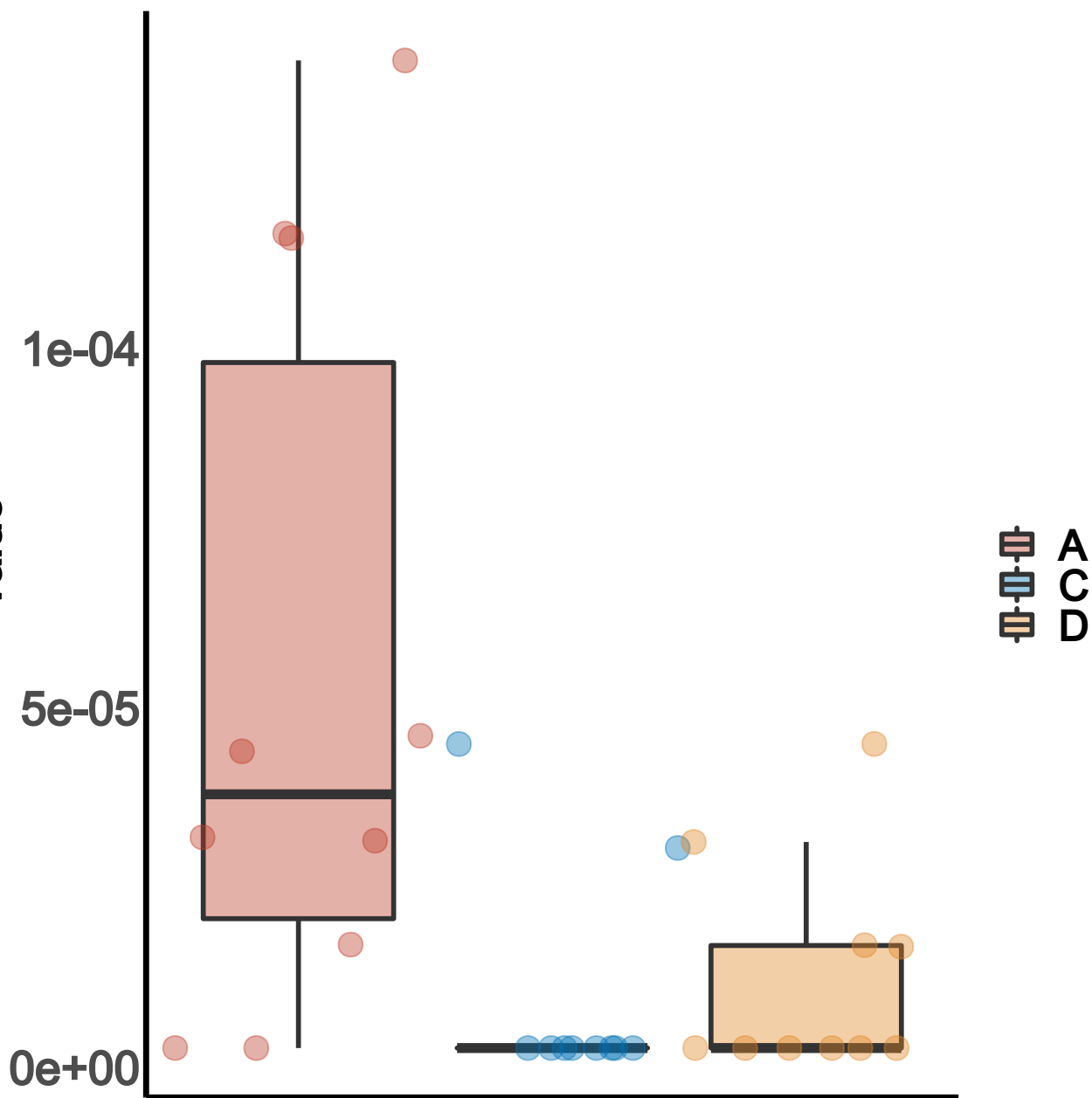

Supplement: Supplementary file 5 [file Data_Sheet_1.ZIP › boxplot/index39_boxplot_ANOVA.pdf]

p-value =  $5.6\text{e-}08$ ; n = 30

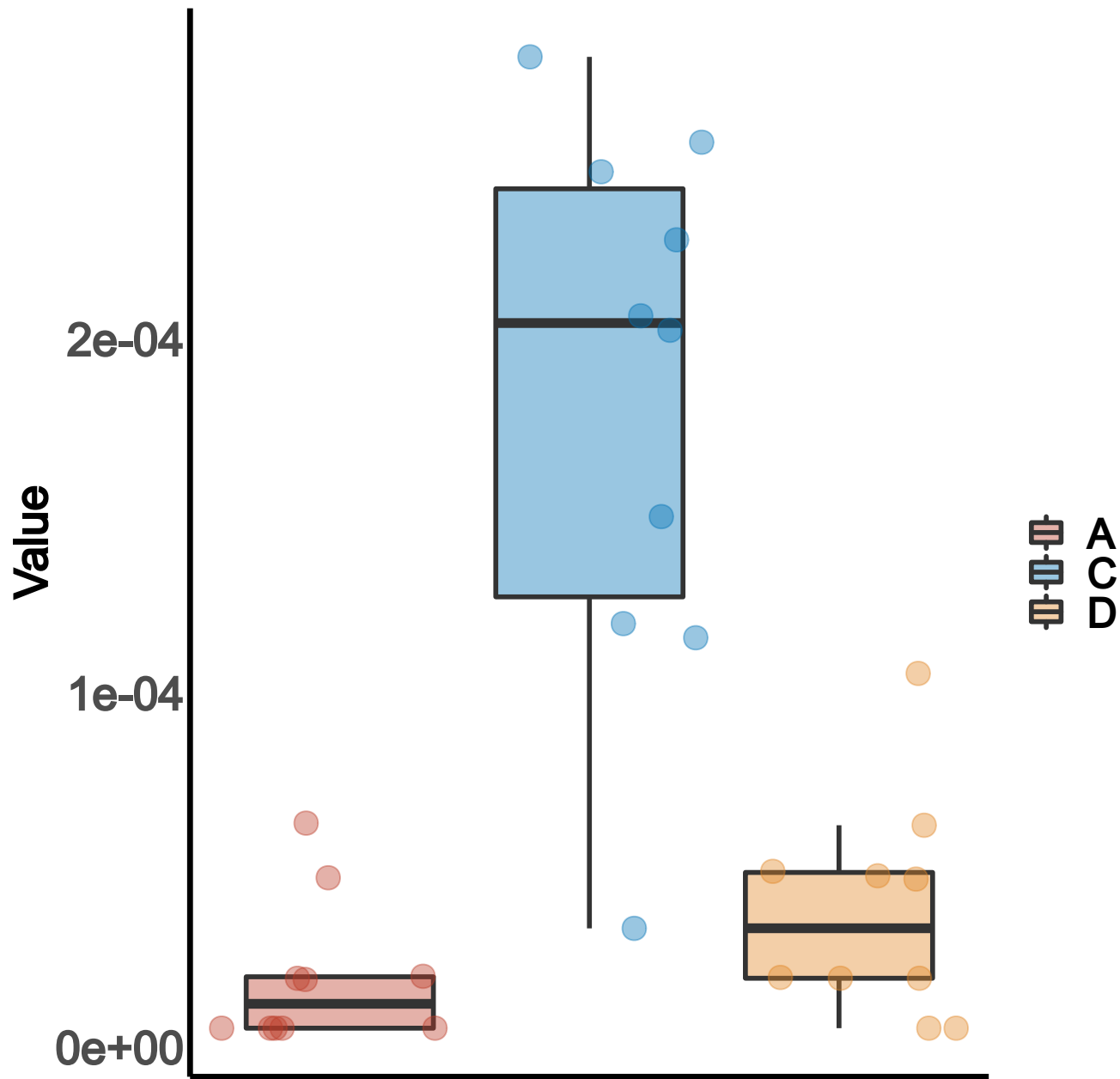

Supplement: Supplementary file 5 [file Data_Sheet_1.ZIP › boxplot/index3_boxplot_ANOVA.pdf]

p-value = 0.006; n = 30

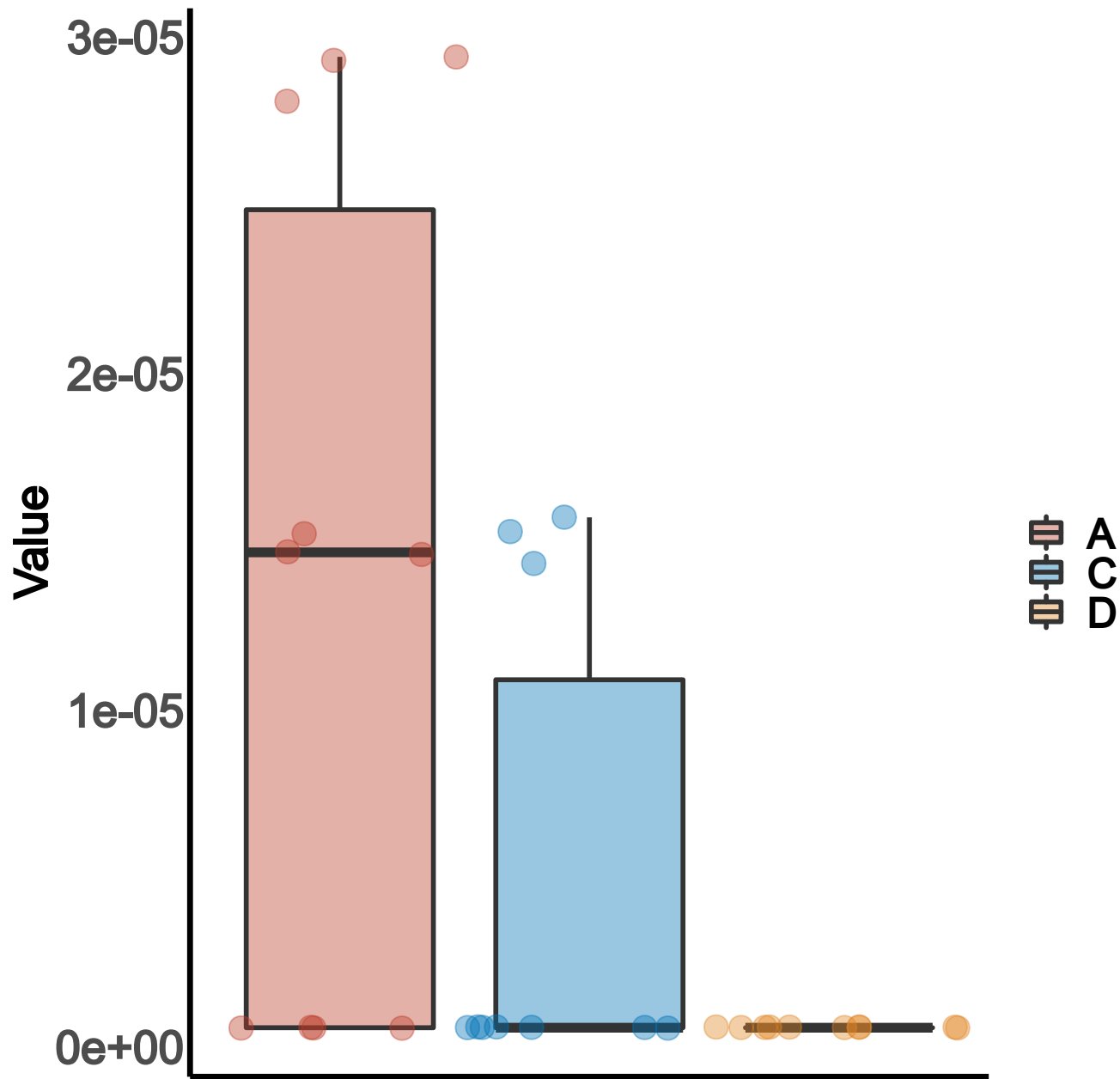

Supplement: Supplementary file 5 [file Data_Sheet_1.ZIP › boxplot/index40_boxplot_ANOVA.pdf]

p-value = 0.006; n = 30

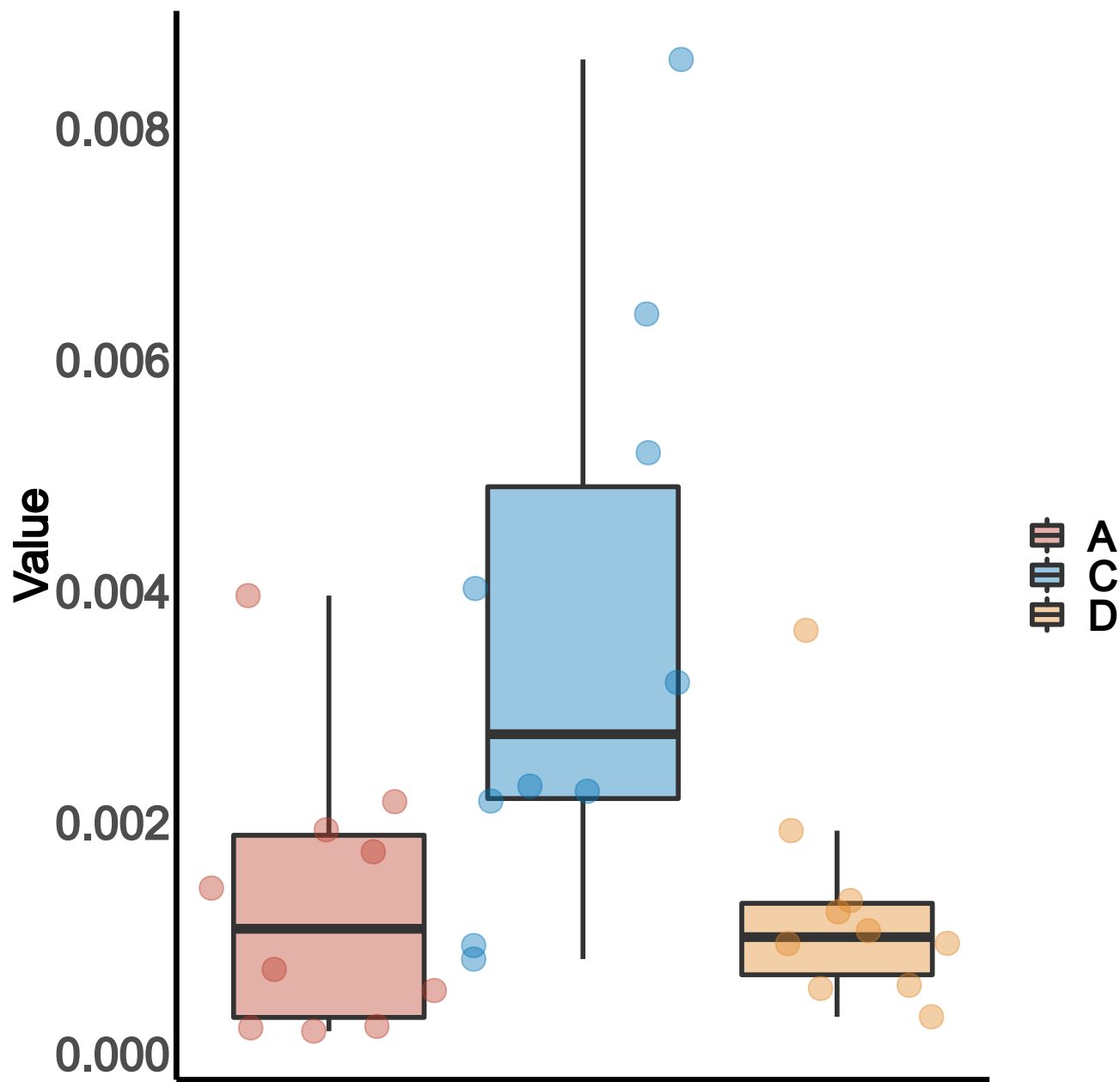

Supplement: Supplementary file 5 [file Data_Sheet_1.ZIP › boxplot/index41_boxplot_ANOVA.pdf]

p-value = 0.0067; n = 30

Value

0e+00

2e-05

4e-05

6e-05

A  
C  
D

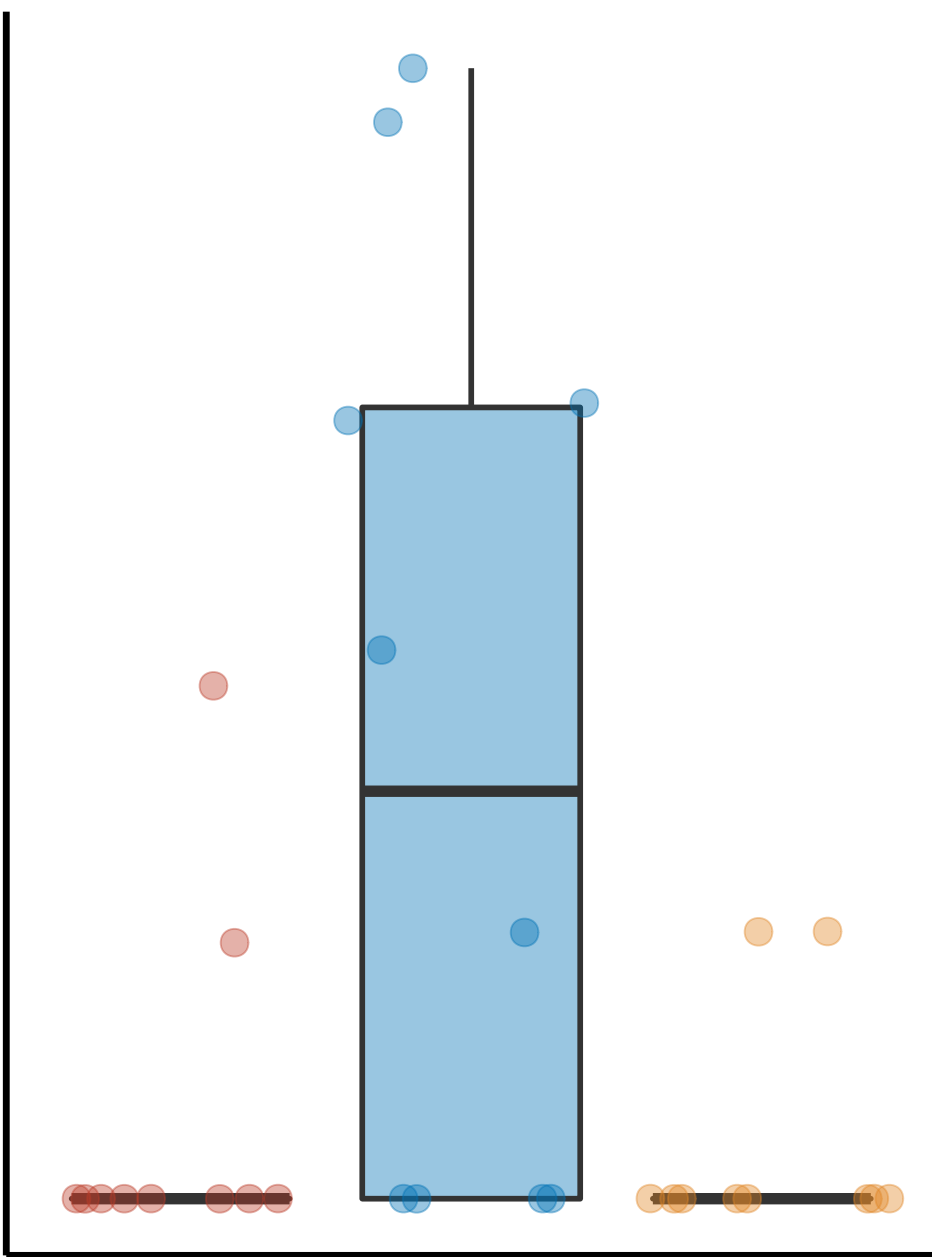

Supplement: Supplementary file 5 [file Data_Sheet_1.ZIP › boxplot/index42_boxplot_ANOVA.pdf]

p-value = 0.0067; n = 30

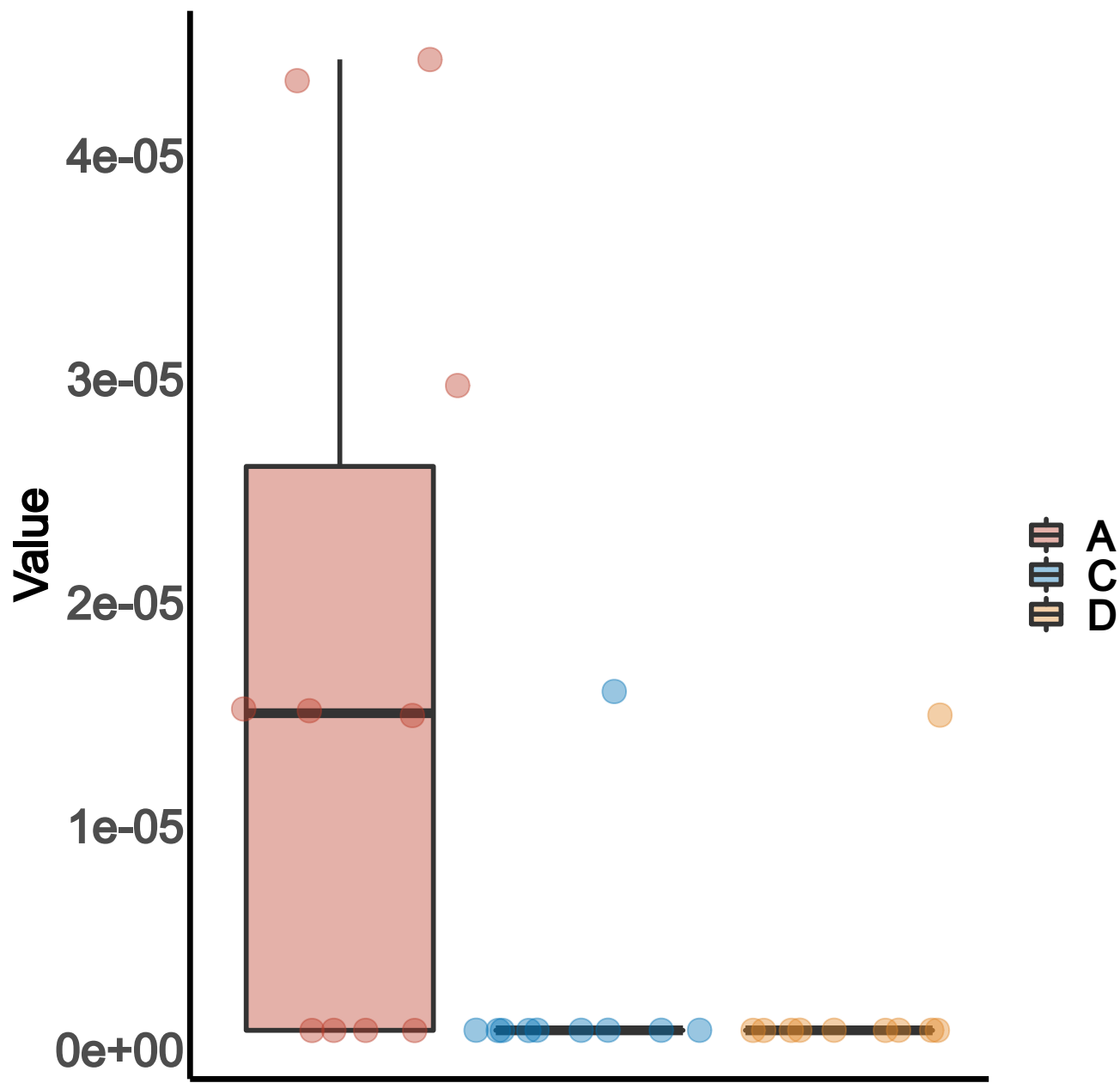

Supplement: Supplementary file 5 [file Data_Sheet_1.ZIP › boxplot/index43_boxplot_ANOVA.pdf]

**p-value = 0.0079; n = 30**

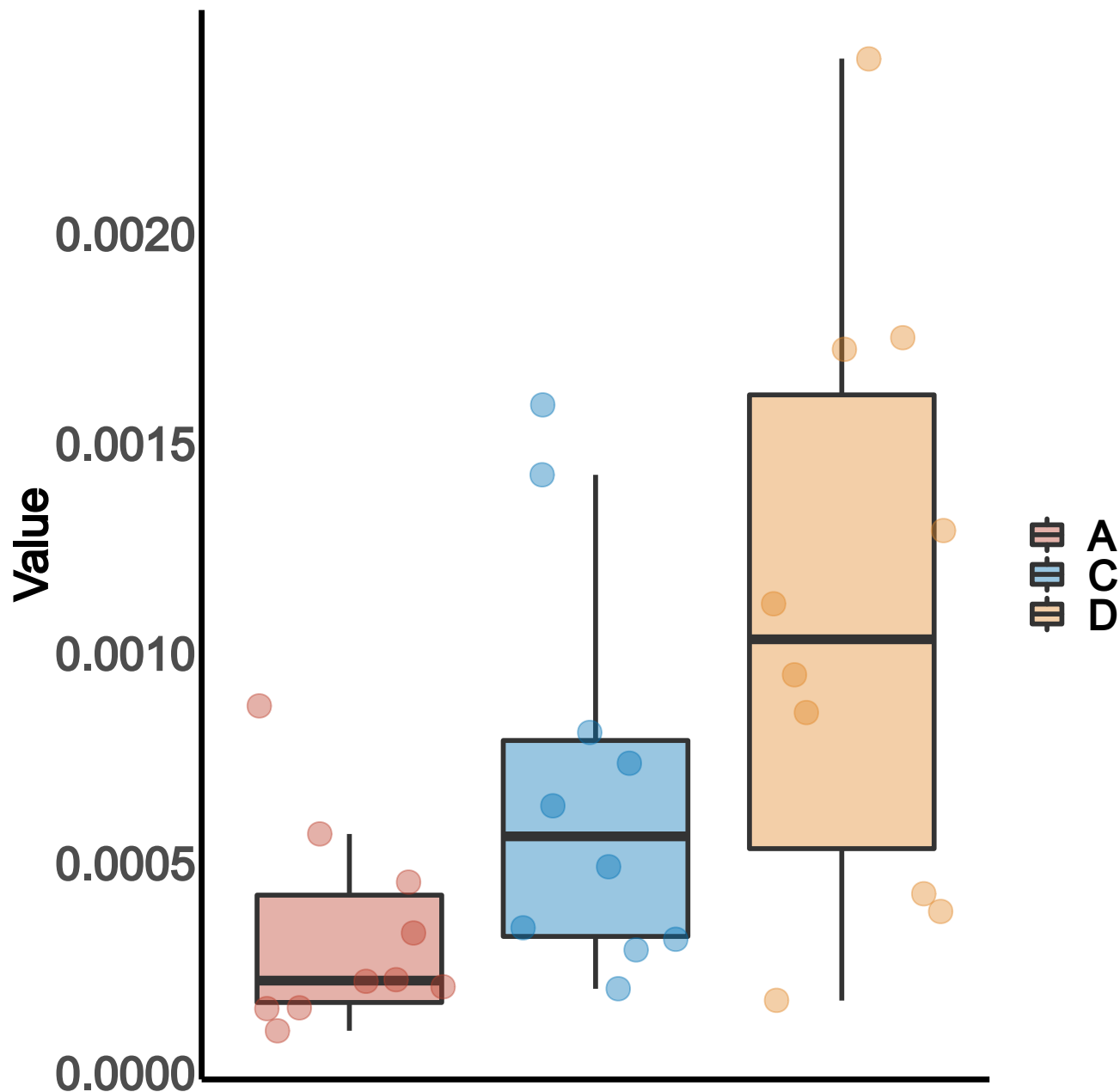

Supplement: Supplementary file 5 [file Data_Sheet_1.ZIP › boxplot/index45_boxplot_ANOVA.pdf]

p-value = 0.0081; n = 30

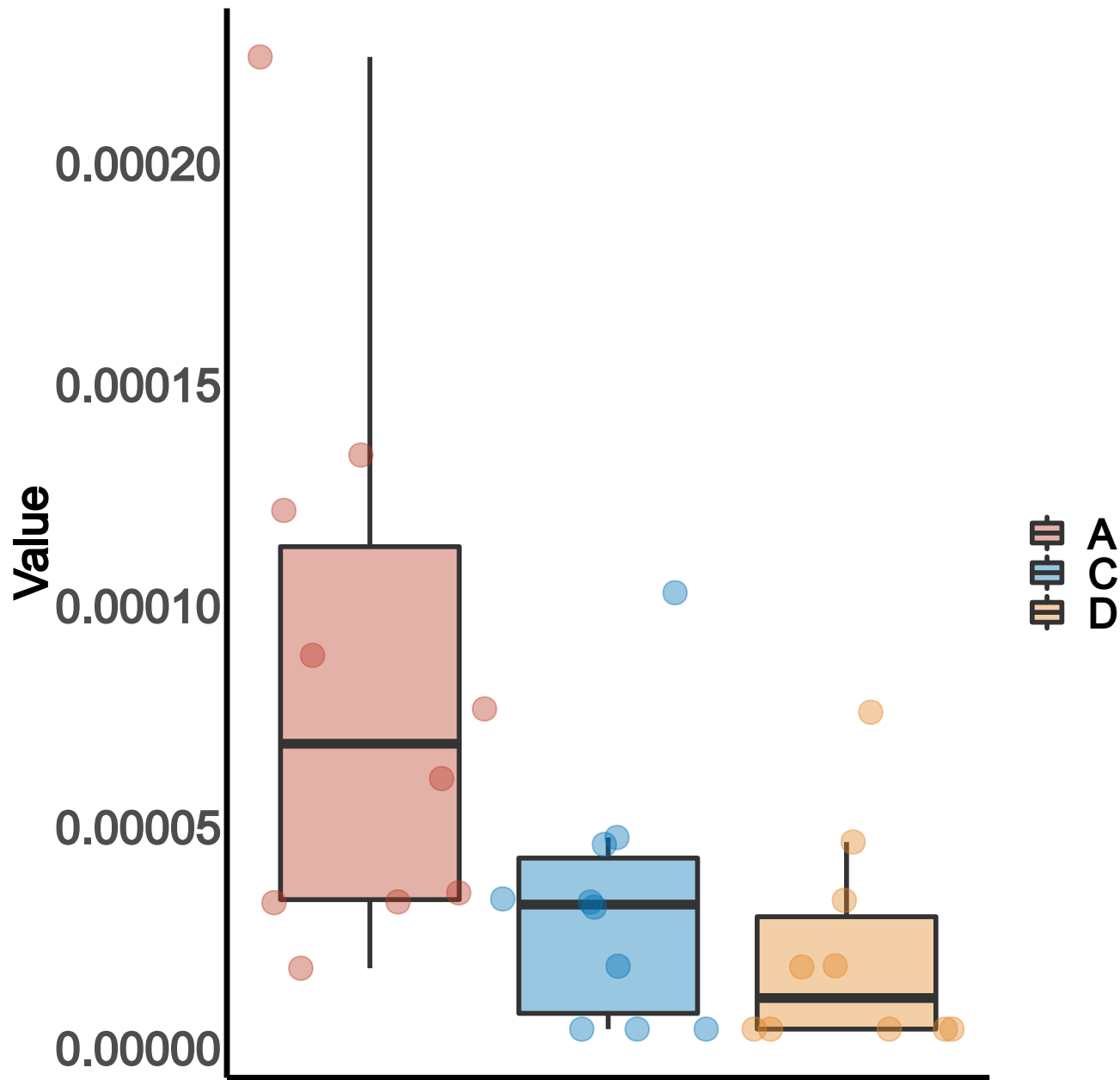

Supplement: Supplementary file 5 [file Data_Sheet_1.ZIP › boxplot/index46_boxplot_ANOVA.pdf]

p-value = 0.0085; n = 30

Value

3e-05  
2e-05  
1e-05  
0e+00

A  
C  
D

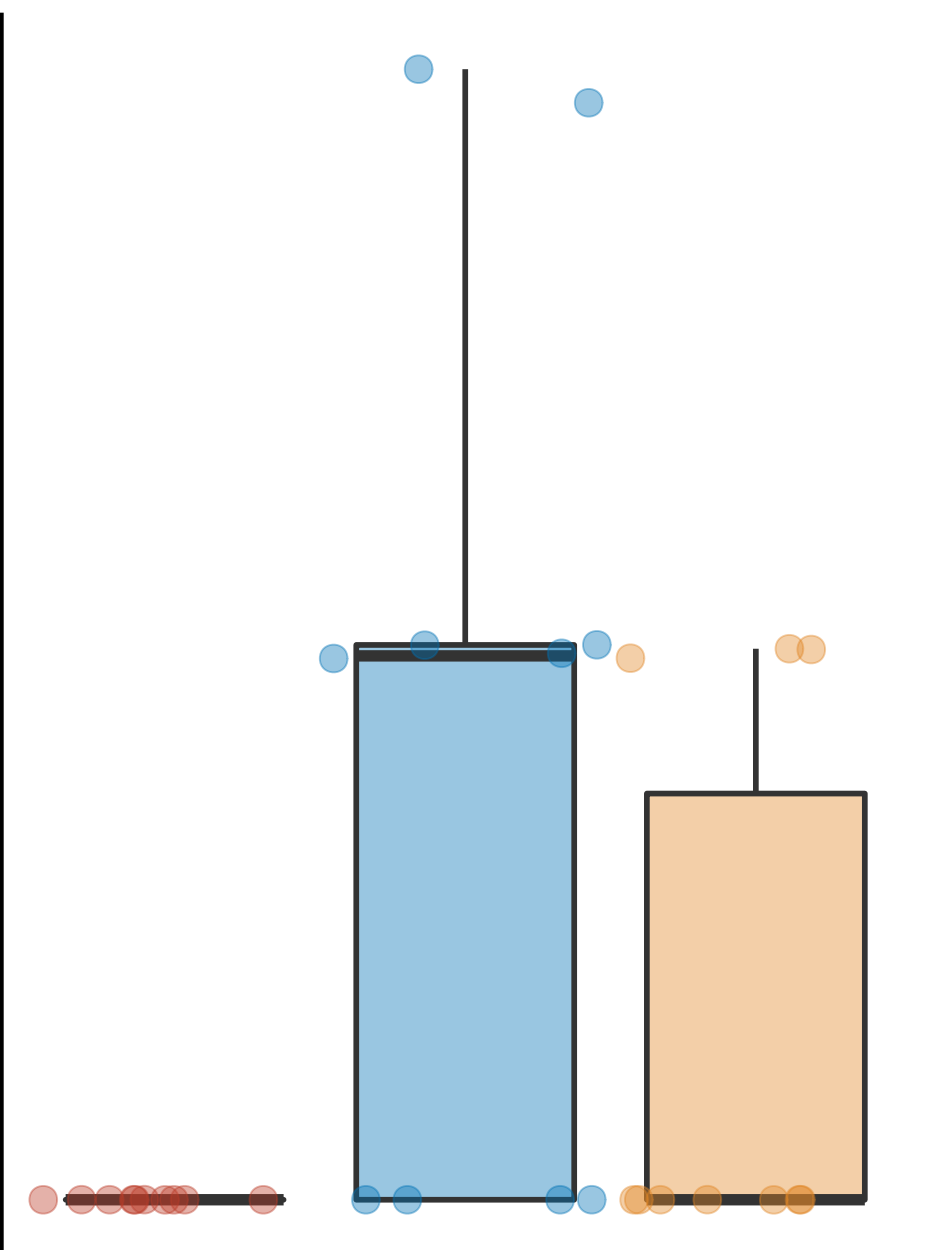

Supplement: Supplementary file 5 [file Data_Sheet_1.ZIP › boxplot/index47_boxplot_ANOVA.pdf]

p-value = 0.009; n = 30

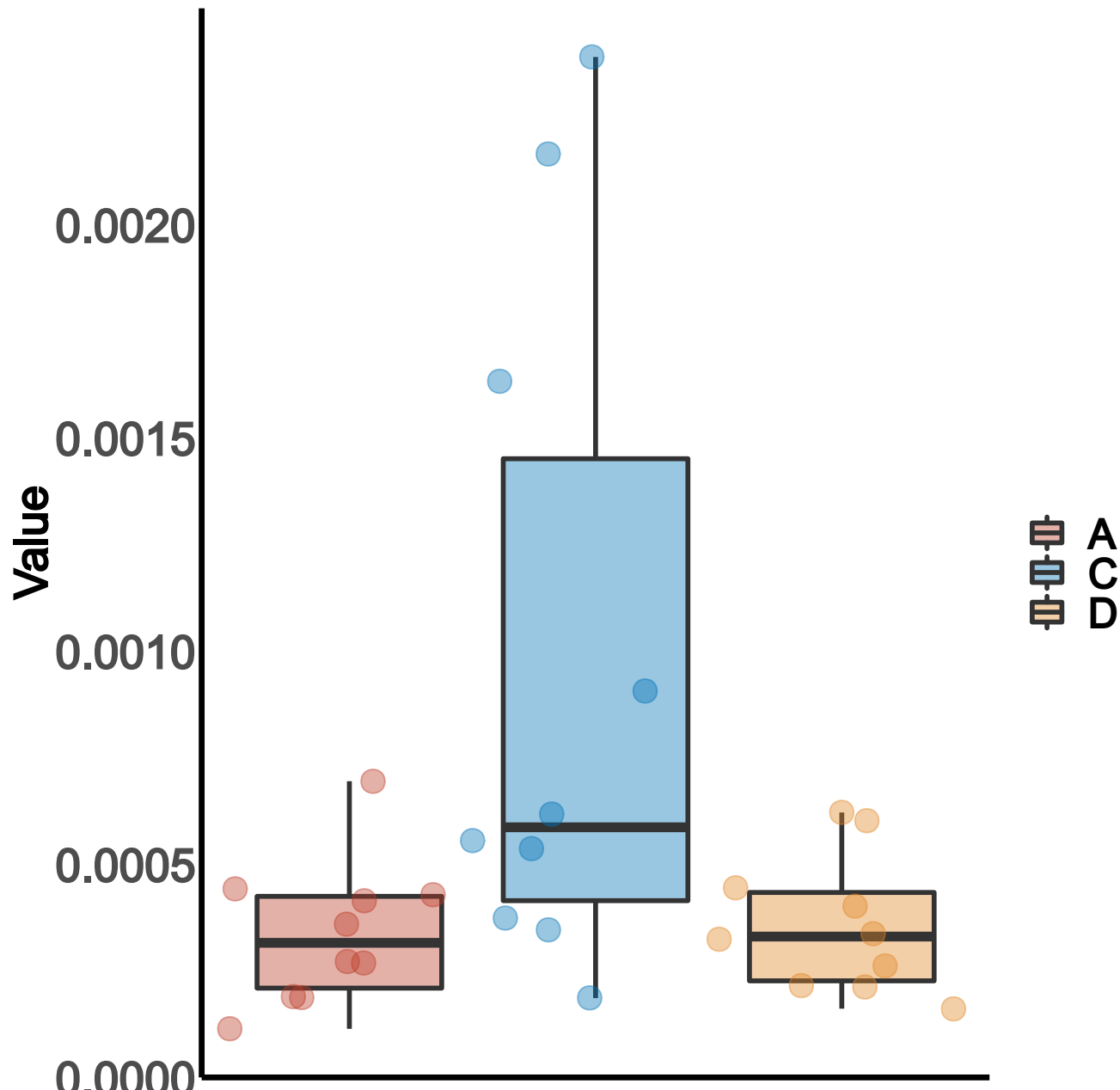

Supplement: Supplementary file 5 [file Data_Sheet_1.ZIP › boxplot/index48_boxplot_ANOVA.pdf]

**p-value =  $1.2\text{e-}07$ ; n = 30**

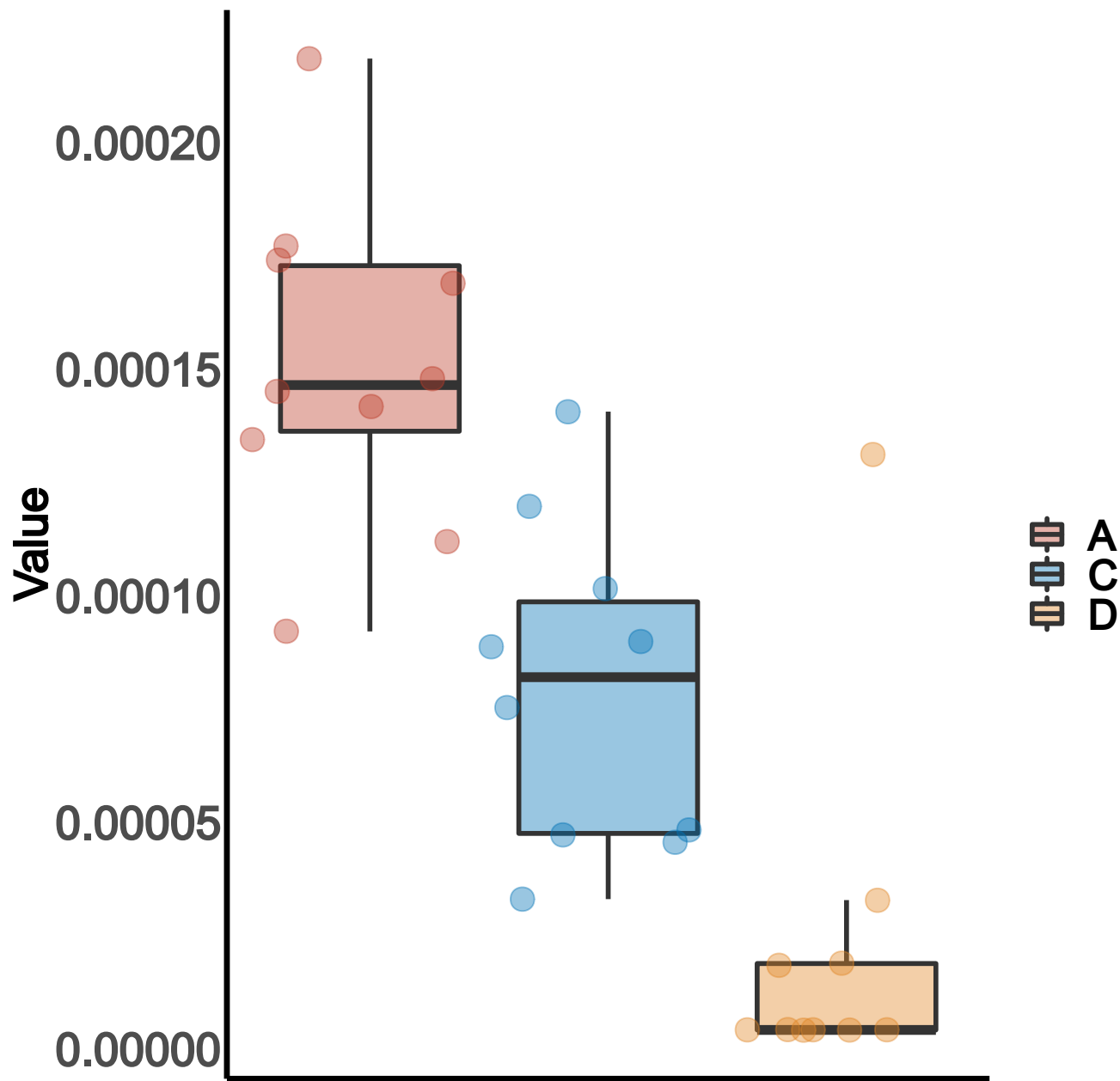

Supplement: Supplementary file 5 [file Data_Sheet_1.ZIP › boxplot/index4_boxplot_ANOVA.pdf]

**p-value = 0.01; n = 30**

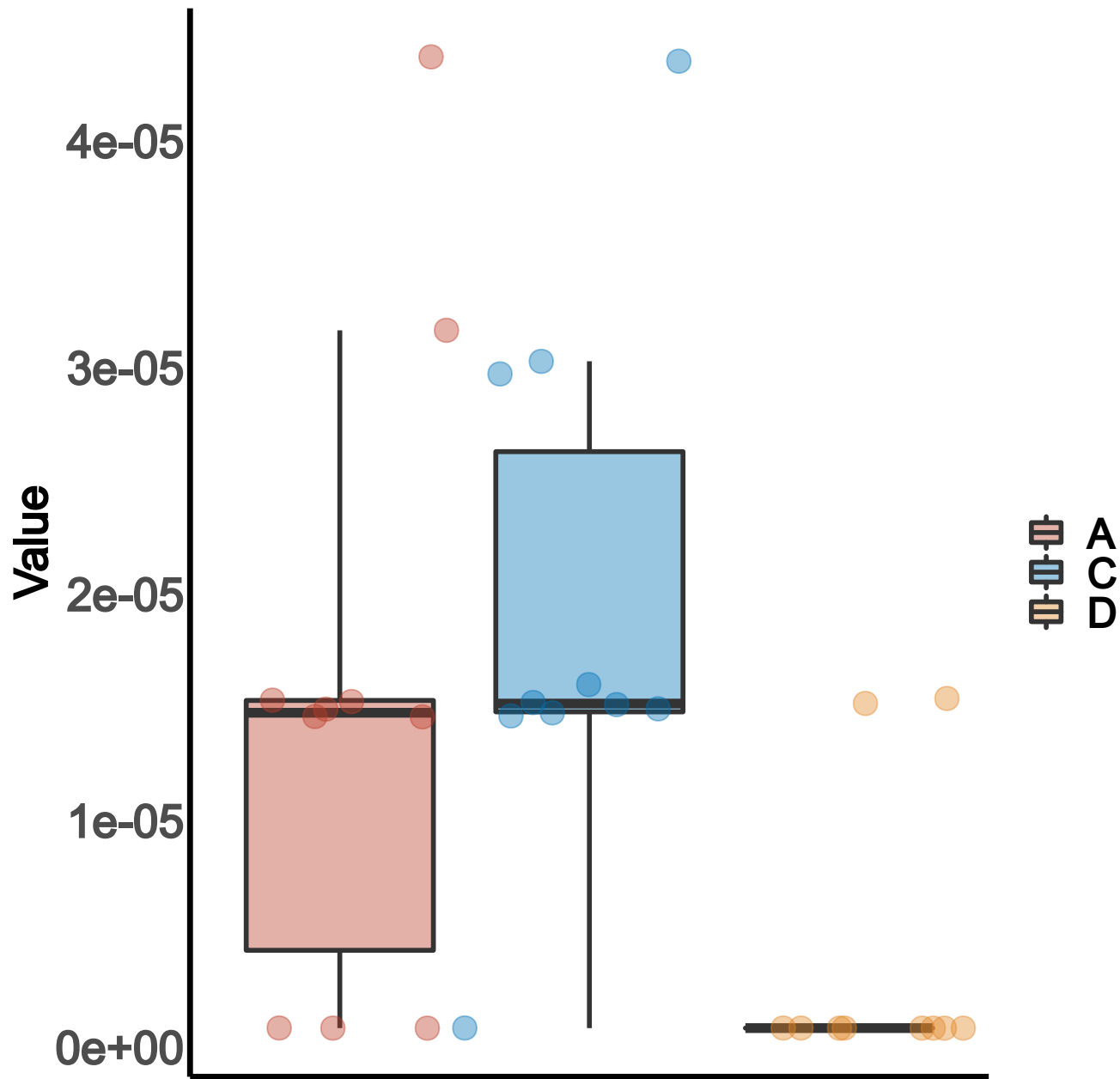

Supplement: Supplementary file 5 [file Data_Sheet_1.ZIP › boxplot/index50_boxplot_ANOVA.pdf]

p-value = 0.01; n = 30

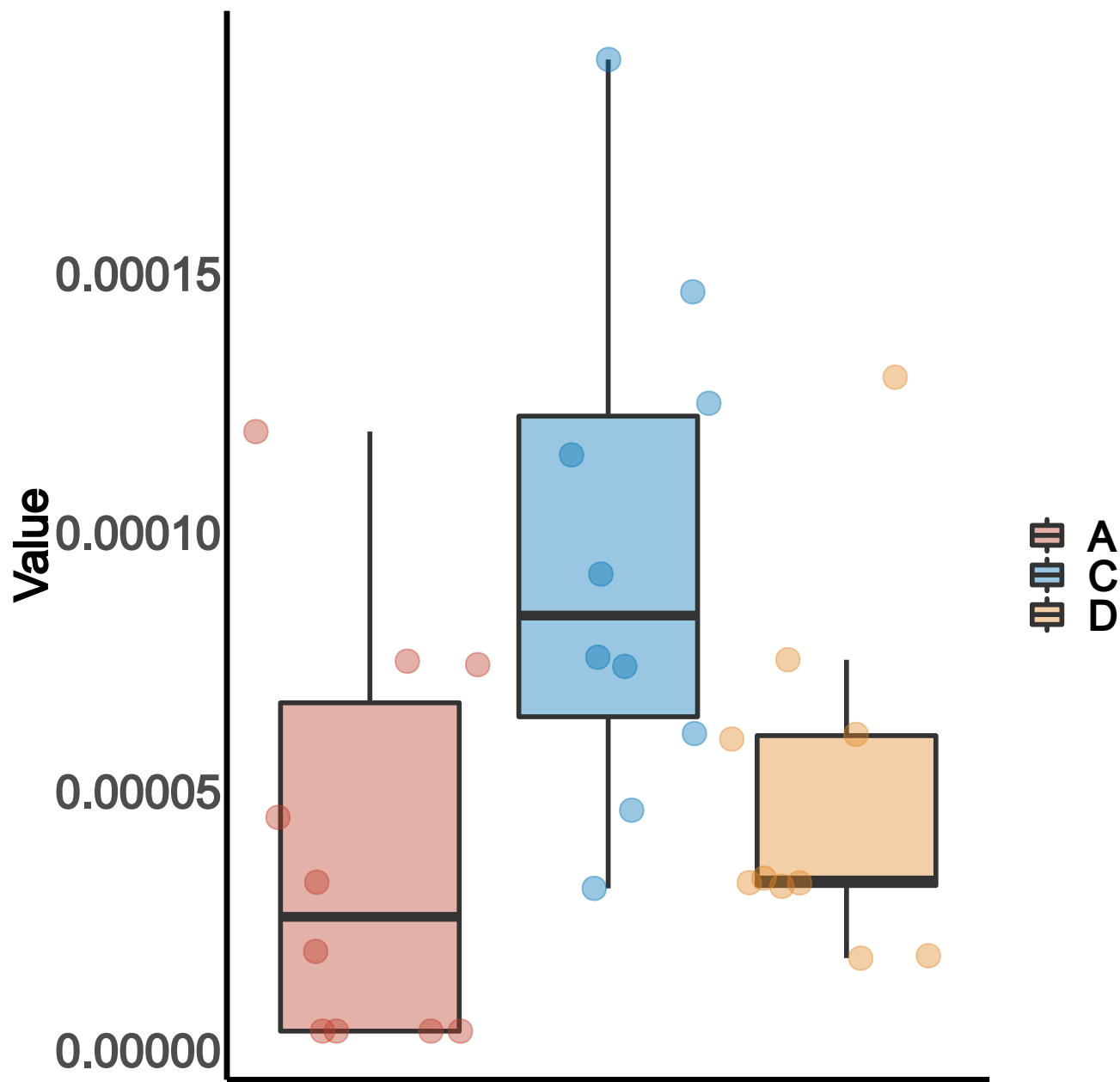

Supplement: Supplementary file 5 [file Data_Sheet_1.ZIP › boxplot/index51_boxplot_ANOVA.pdf]

**p-value = 0.01; n = 30**

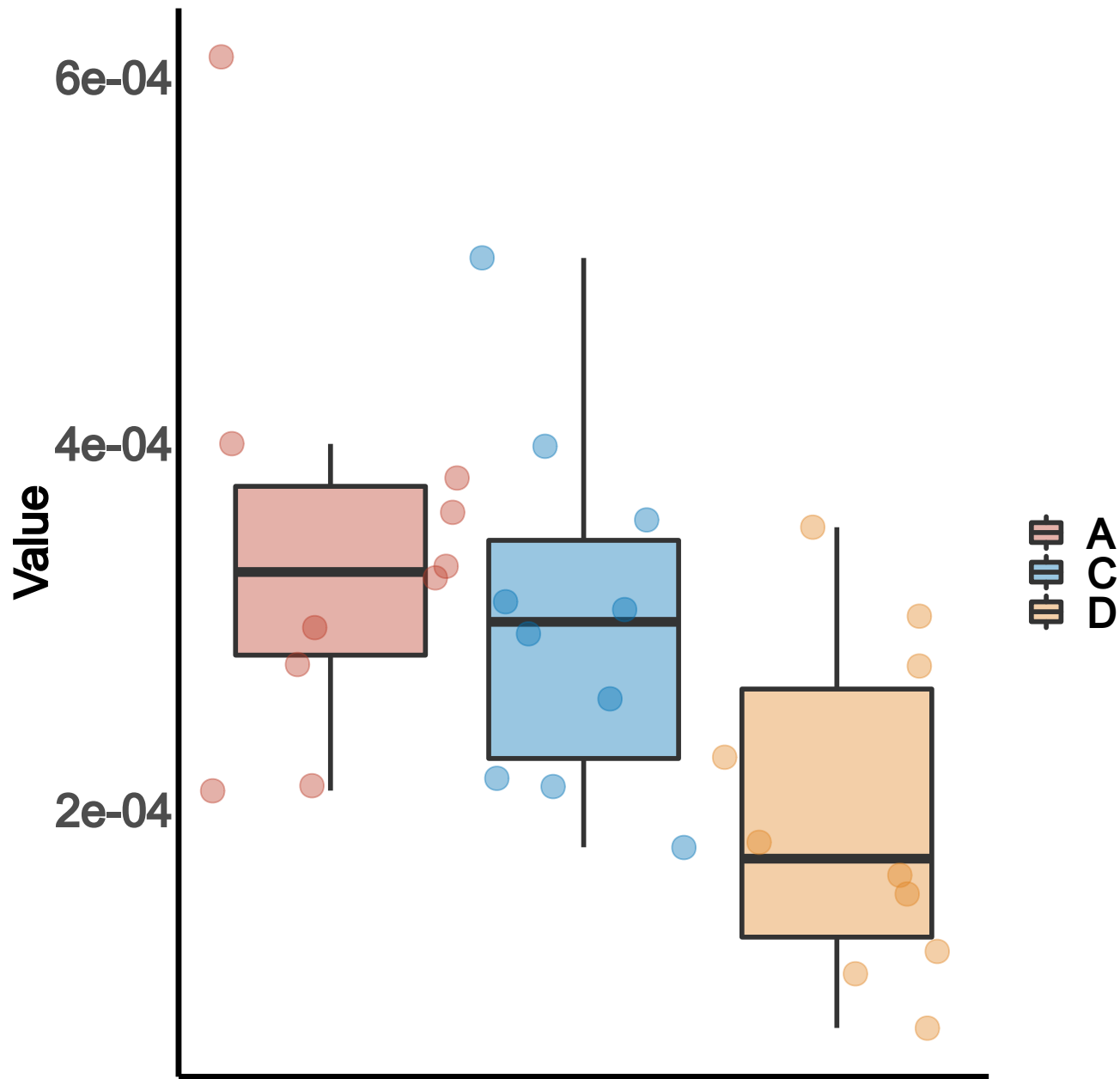

Supplement: Supplementary file 5 [file Data_Sheet_1.ZIP › boxplot/index52_boxplot_ANOVA.pdf]

p-value = 0.011; n = 30

Value

0e+00

4e-04

2e-04

A  
C  
D

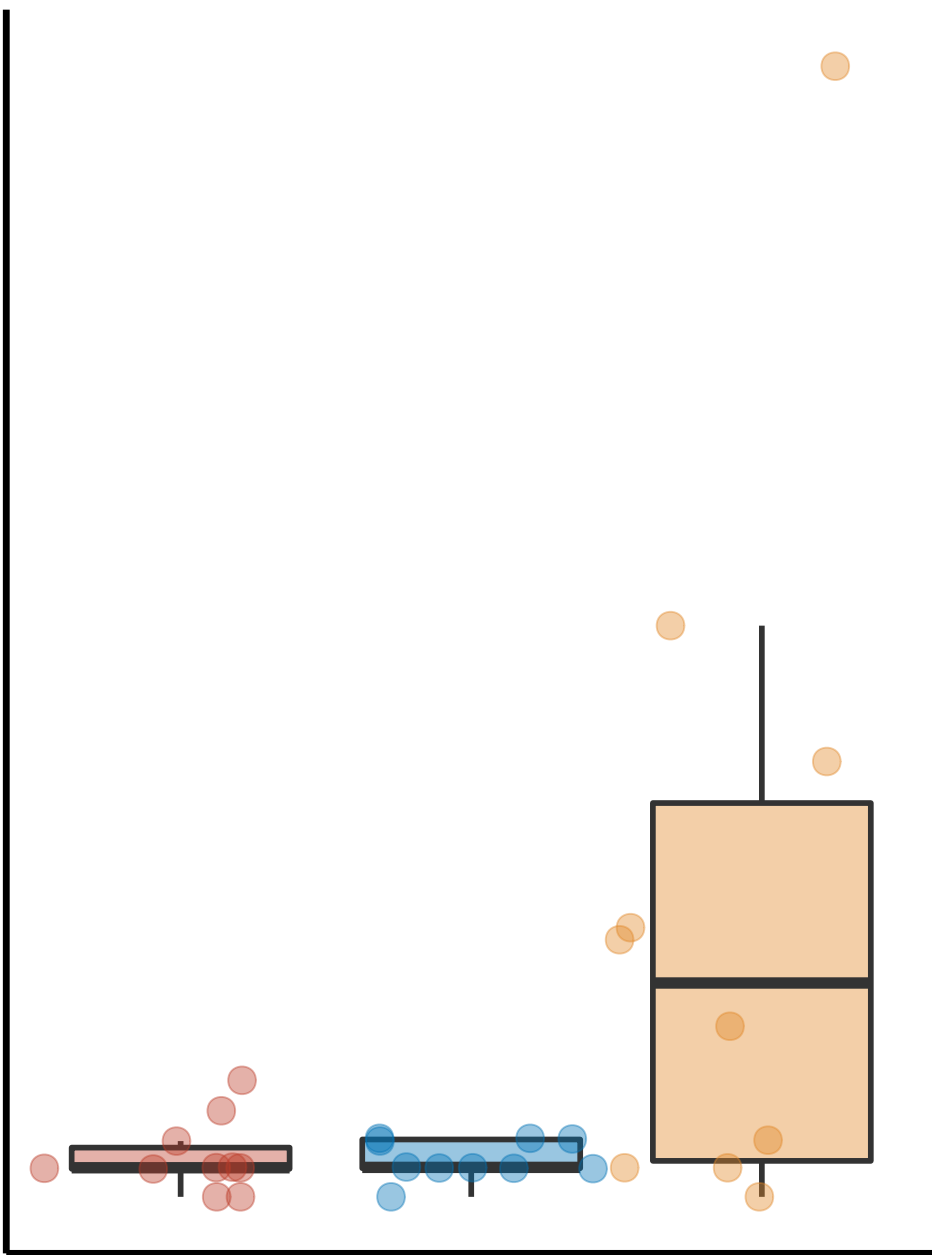

Supplement: Supplementary file 5 [file Data_Sheet_1.ZIP › boxplot/index53_boxplot_ANOVA.pdf]

p-value = 0.012; n = 30

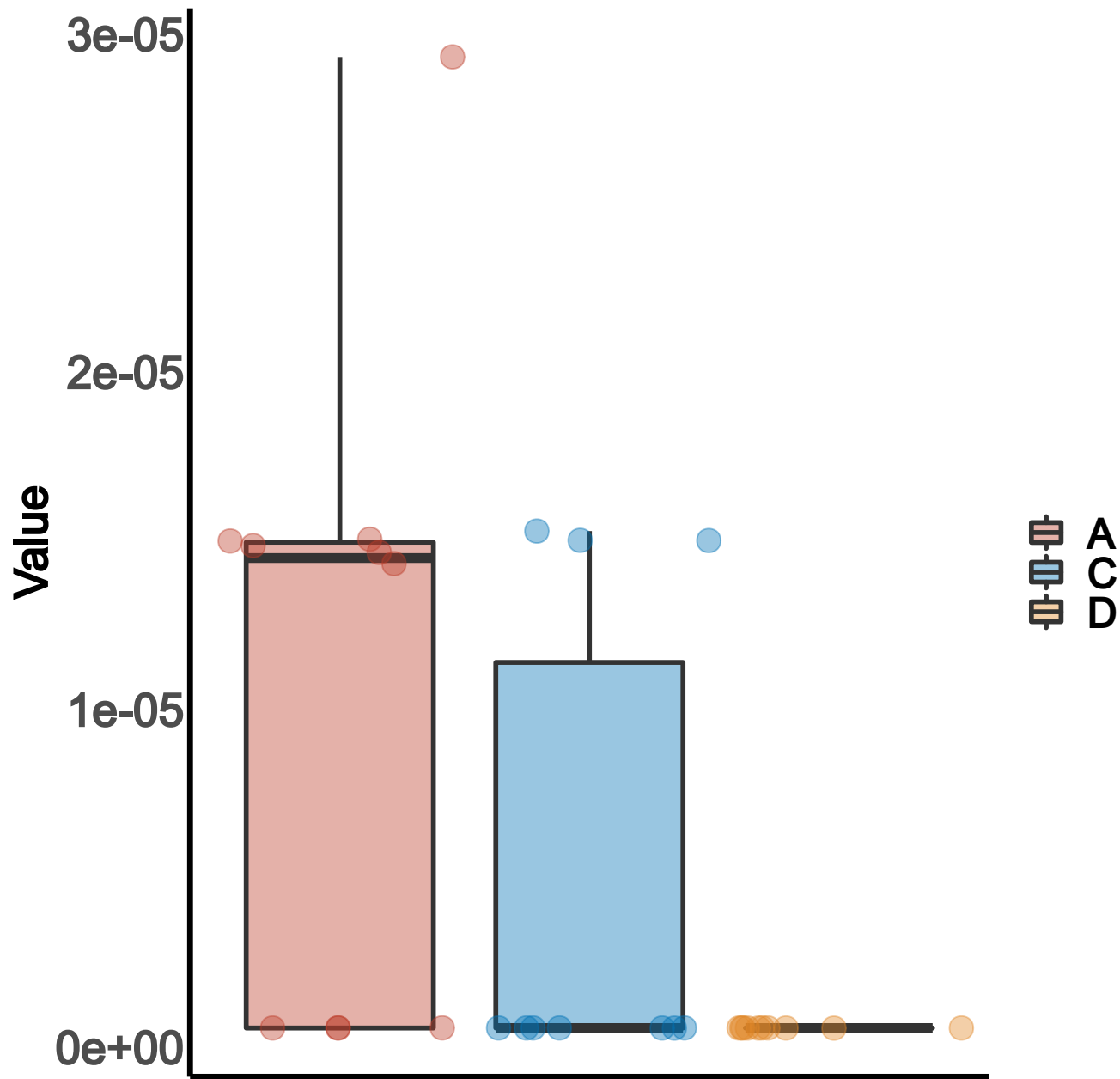

Supplement: Supplementary file 5 [file Data_Sheet_1.ZIP › boxplot/index54_boxplot_ANOVA.pdf]

p-value = 0.013; n = 30

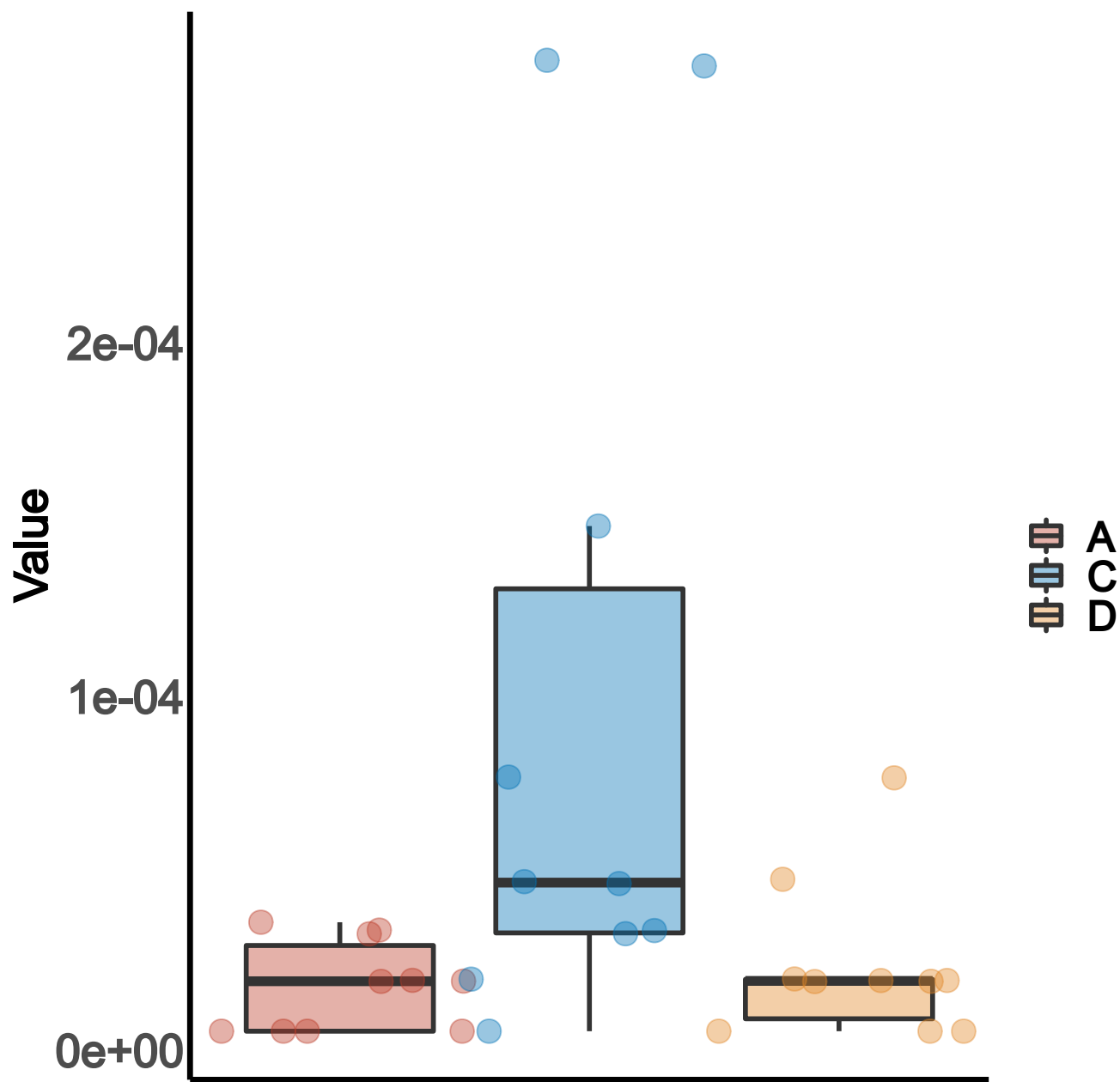

Supplement: Supplementary file 5 [file Data_Sheet_1.ZIP › boxplot/index55_boxplot_ANOVA.pdf]

p-value = 0.015; n = 30

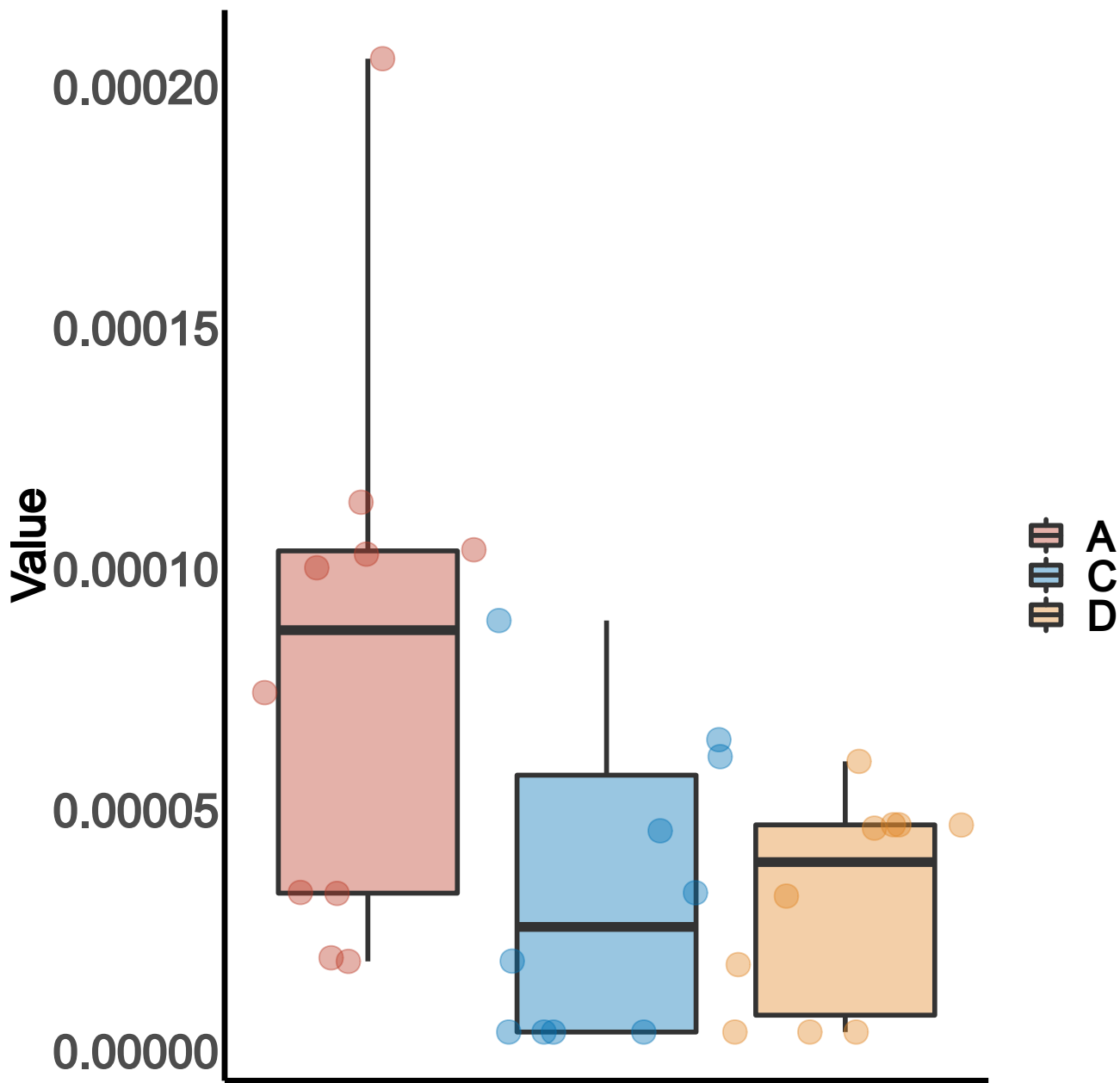

Supplement: Supplementary file 5 [file Data_Sheet_1.ZIP › boxplot/index56_boxplot_ANOVA.pdf]

p-value = 0.016; n = 30

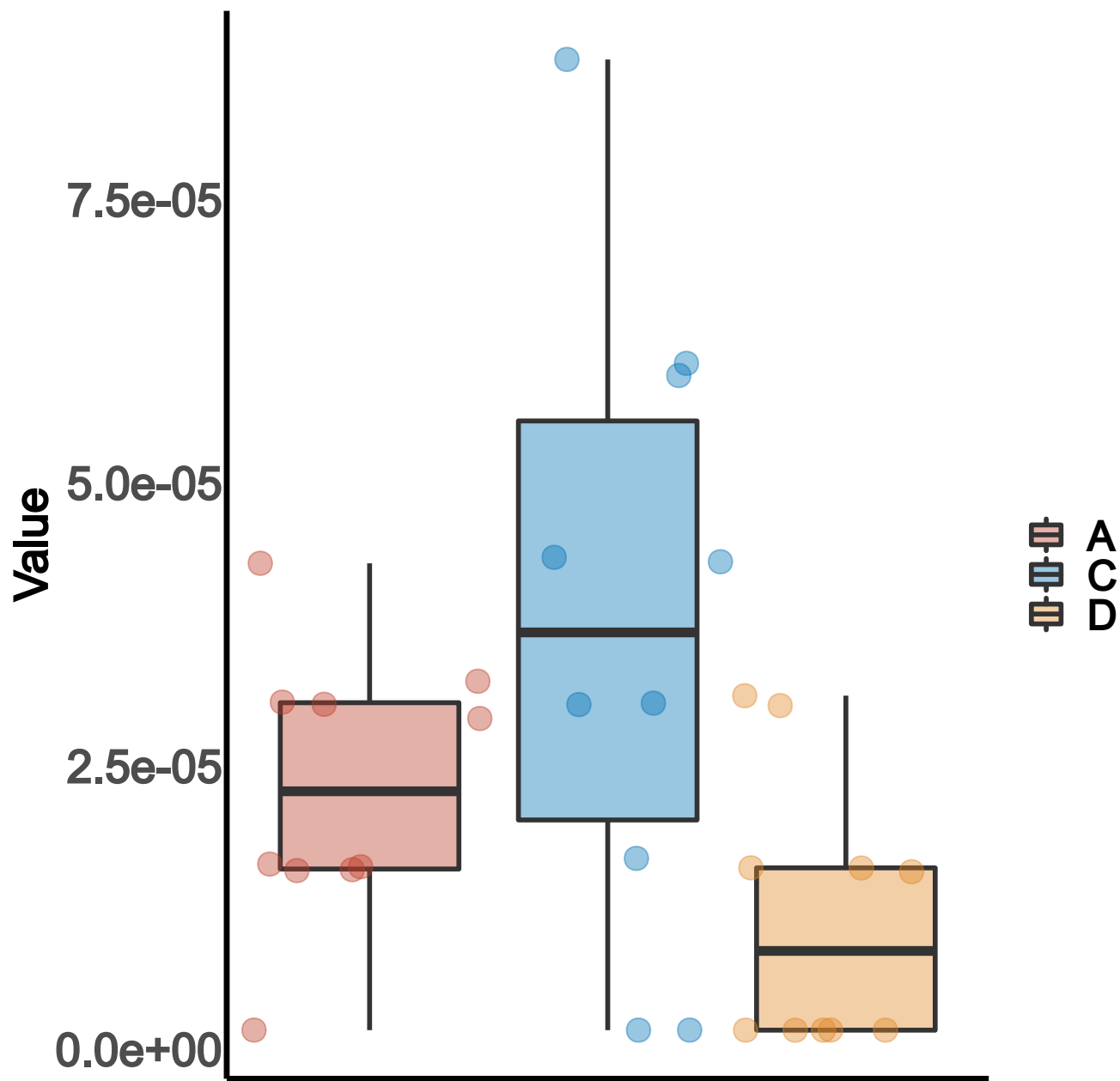

Supplement: Supplementary file 5 [file Data_Sheet_1.ZIP › boxplot/index57_boxplot_ANOVA.pdf]

**p-value = 0.016; n = 30**

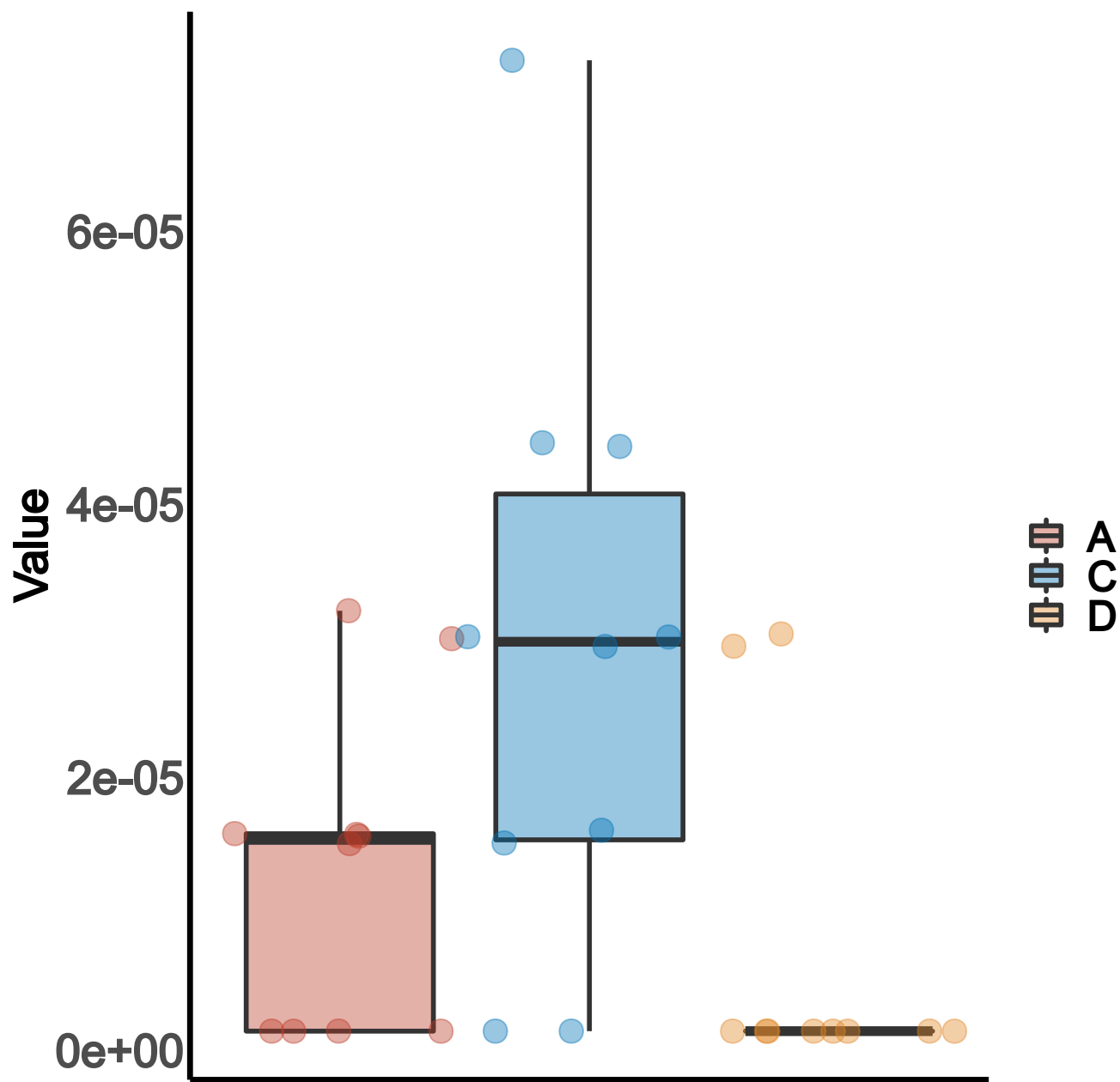

Supplement: Supplementary file 5 [file Data_Sheet_1.ZIP › boxplot/index58_boxplot_ANOVA.pdf]

p-value = 0.016; n = 30

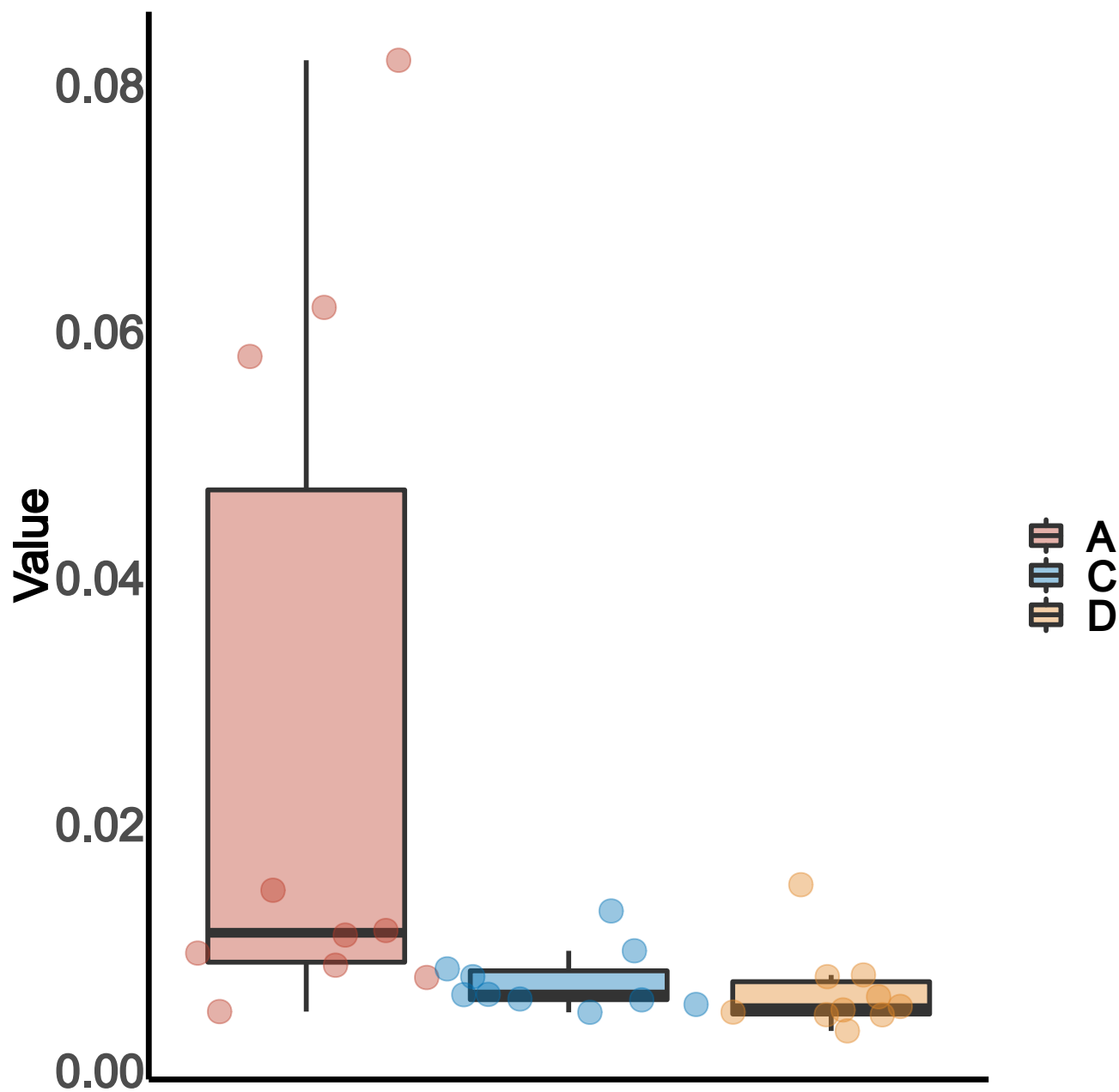

Supplement: Supplementary file 5 [file Data_Sheet_1.ZIP › boxplot/index59_boxplot_ANOVA.pdf]

p-value =  $5e-07$ ; n = 30

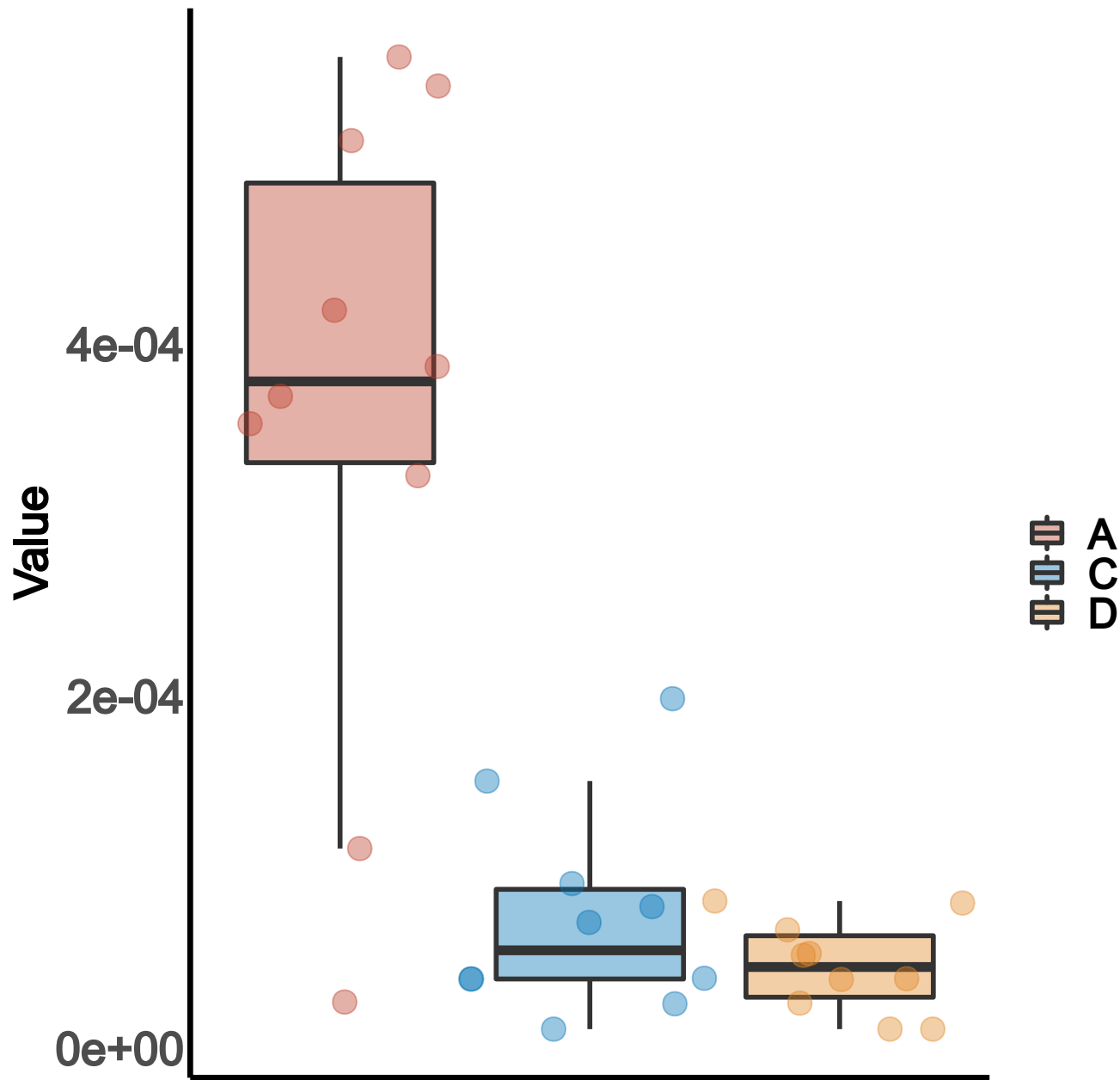

Supplement: Supplementary file 5 [file Data_Sheet_1.ZIP › boxplot/index5_boxplot_ANOVA.pdf]

p-value = 0.017; n = 30

Value

0.00015

0.00010

0.00005

0.00000

A  
C  
D

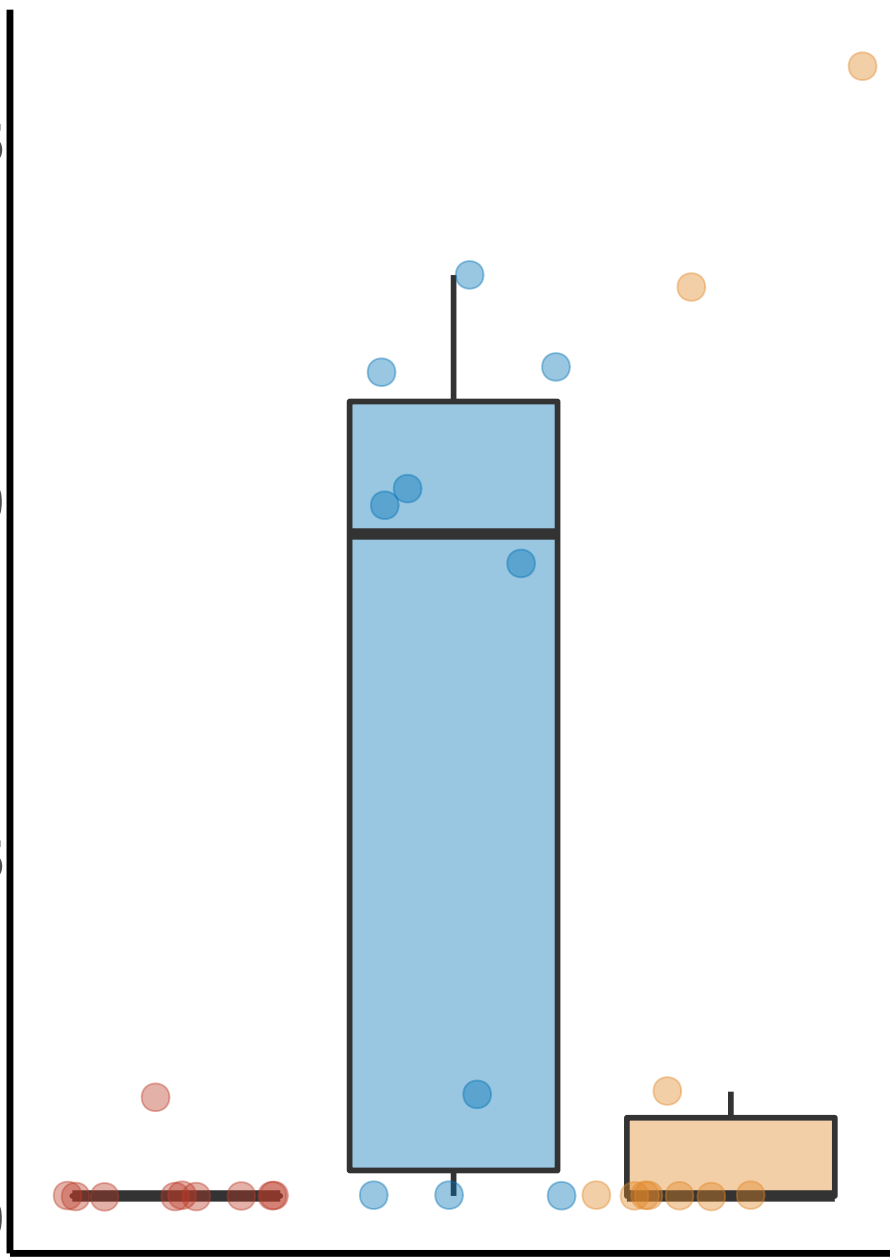

Supplement: Supplementary file 5 [file Data_Sheet_1.ZIP › boxplot/index60_boxplot_ANOVA.pdf]

p-value = 0.018; n = 30

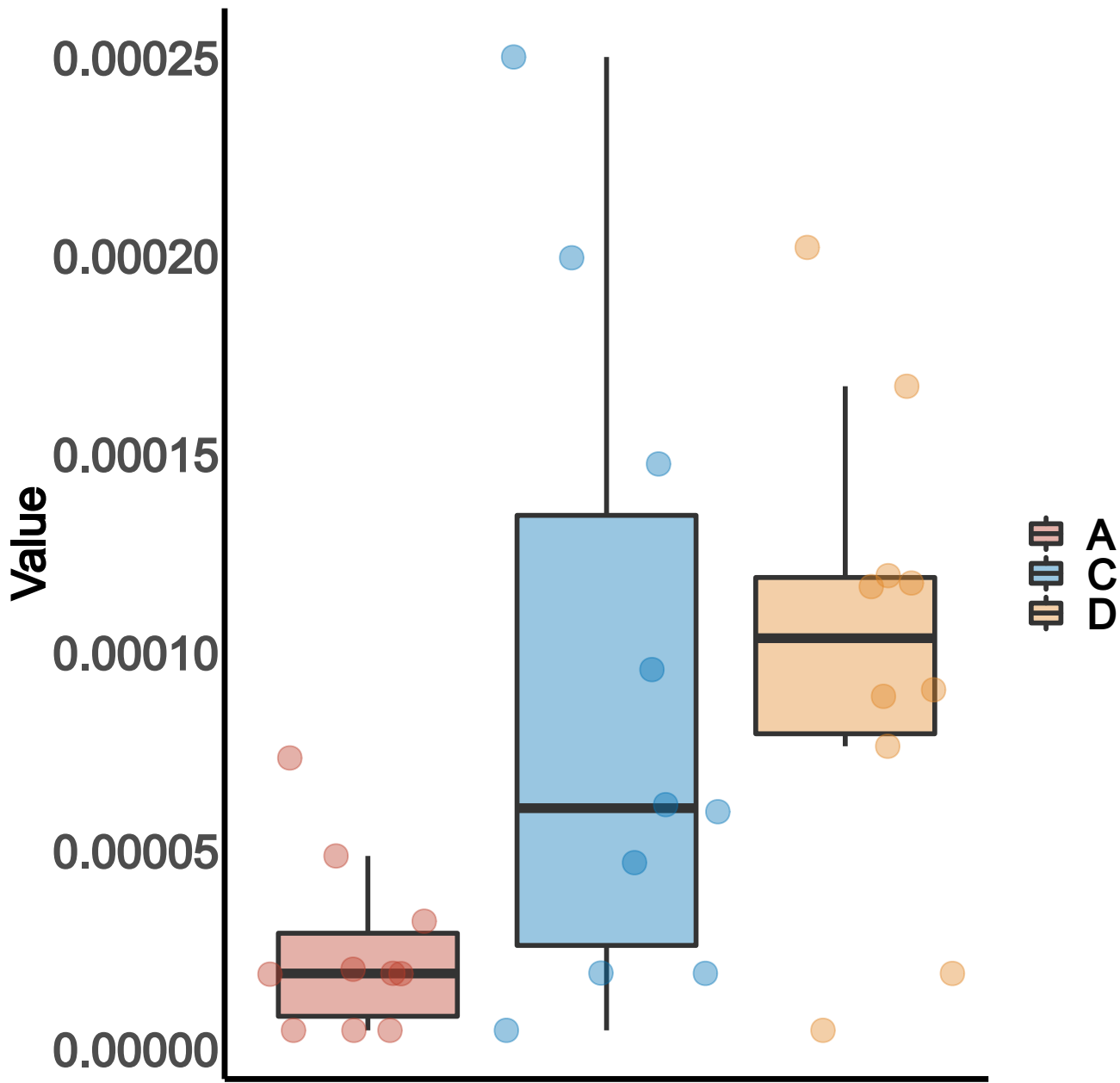

Supplement: Supplementary file 5 [file Data_Sheet_1.ZIP › boxplot/index61_boxplot_ANOVA.pdf]

p-value = 0.019; n = 30

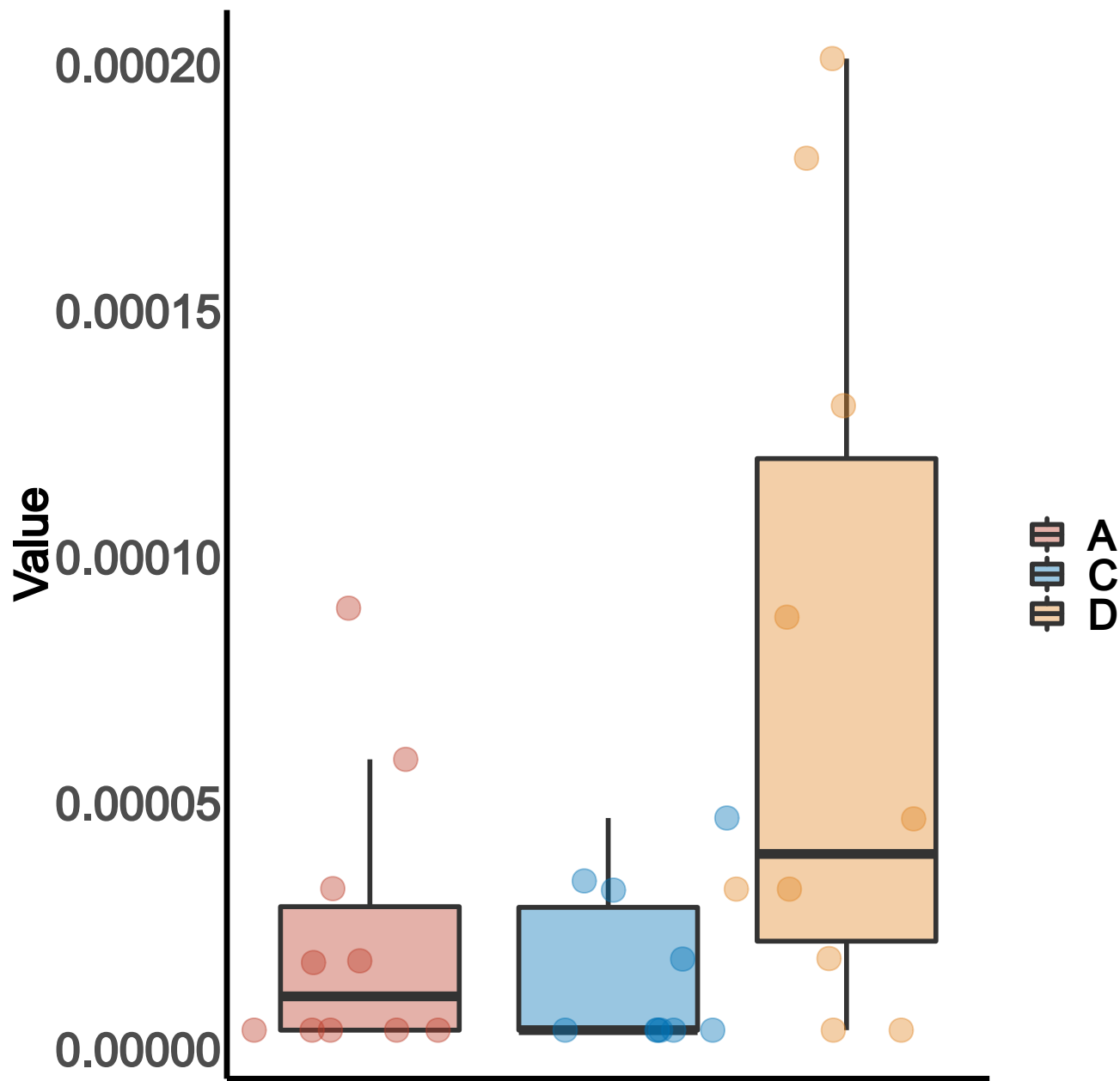

Supplement: Supplementary file 5 [file Data_Sheet_1.ZIP › boxplot/index62_boxplot_ANOVA.pdf]

**p-value = 0.019; n = 30**

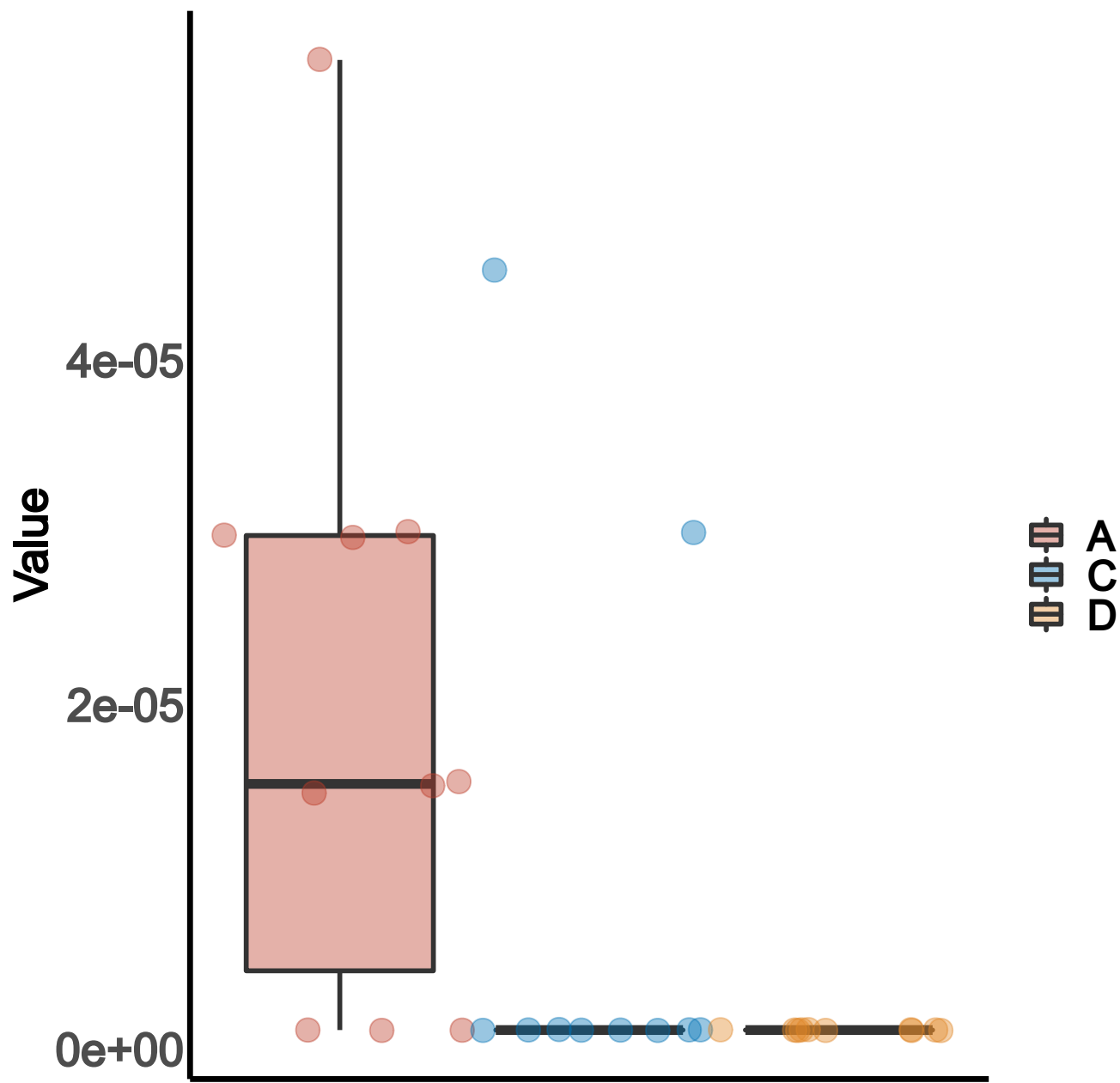

Supplement: Supplementary file 5 [file Data_Sheet_1.ZIP › boxplot/index63_boxplot_ANOVA.pdf]

p-value = 0.02; n = 30

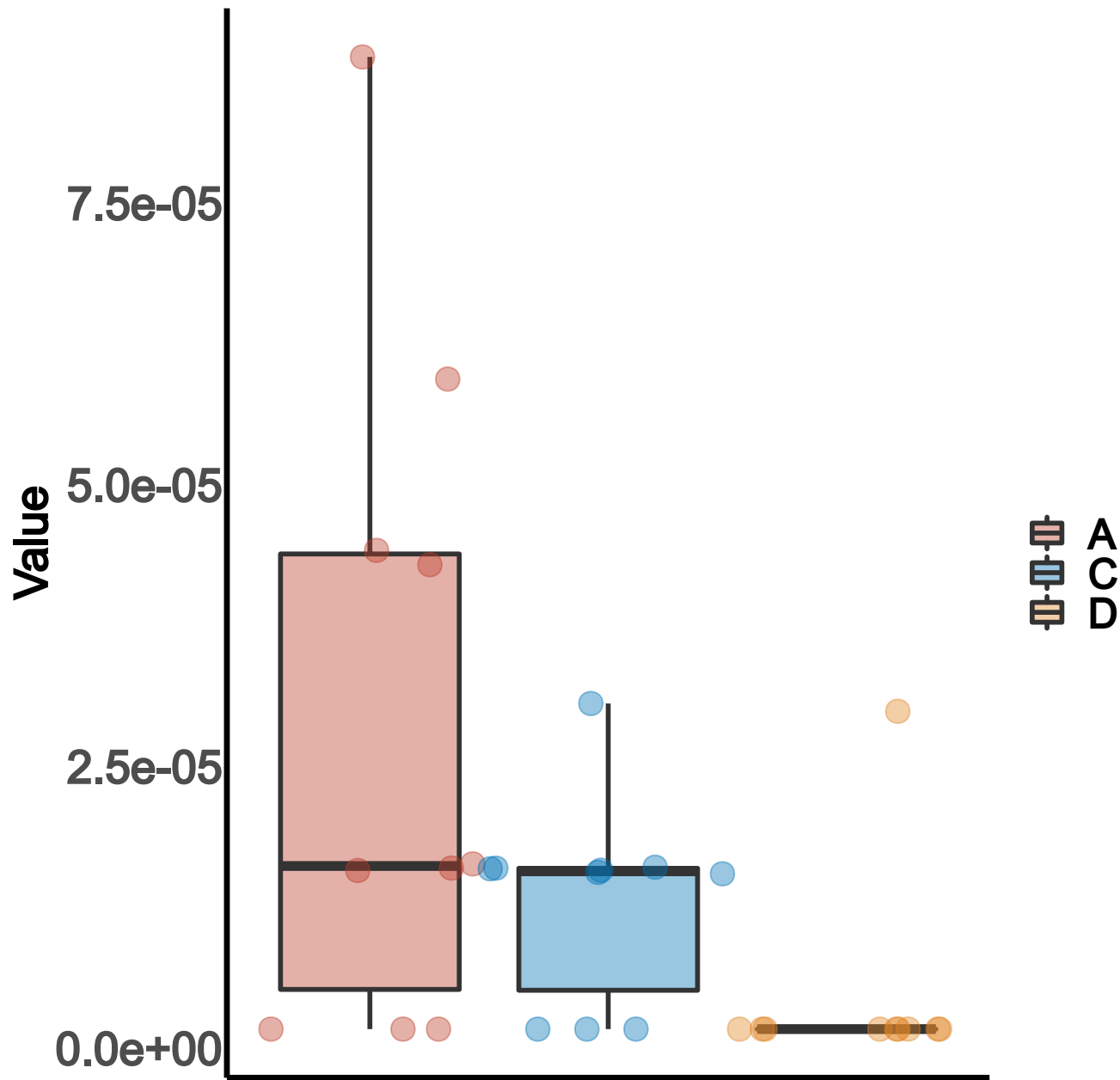

Supplement: Supplementary file 5 [file Data_Sheet_1.ZIP › boxplot/index64_boxplot_ANOVA.pdf]

p-value = 0.022; n = 30

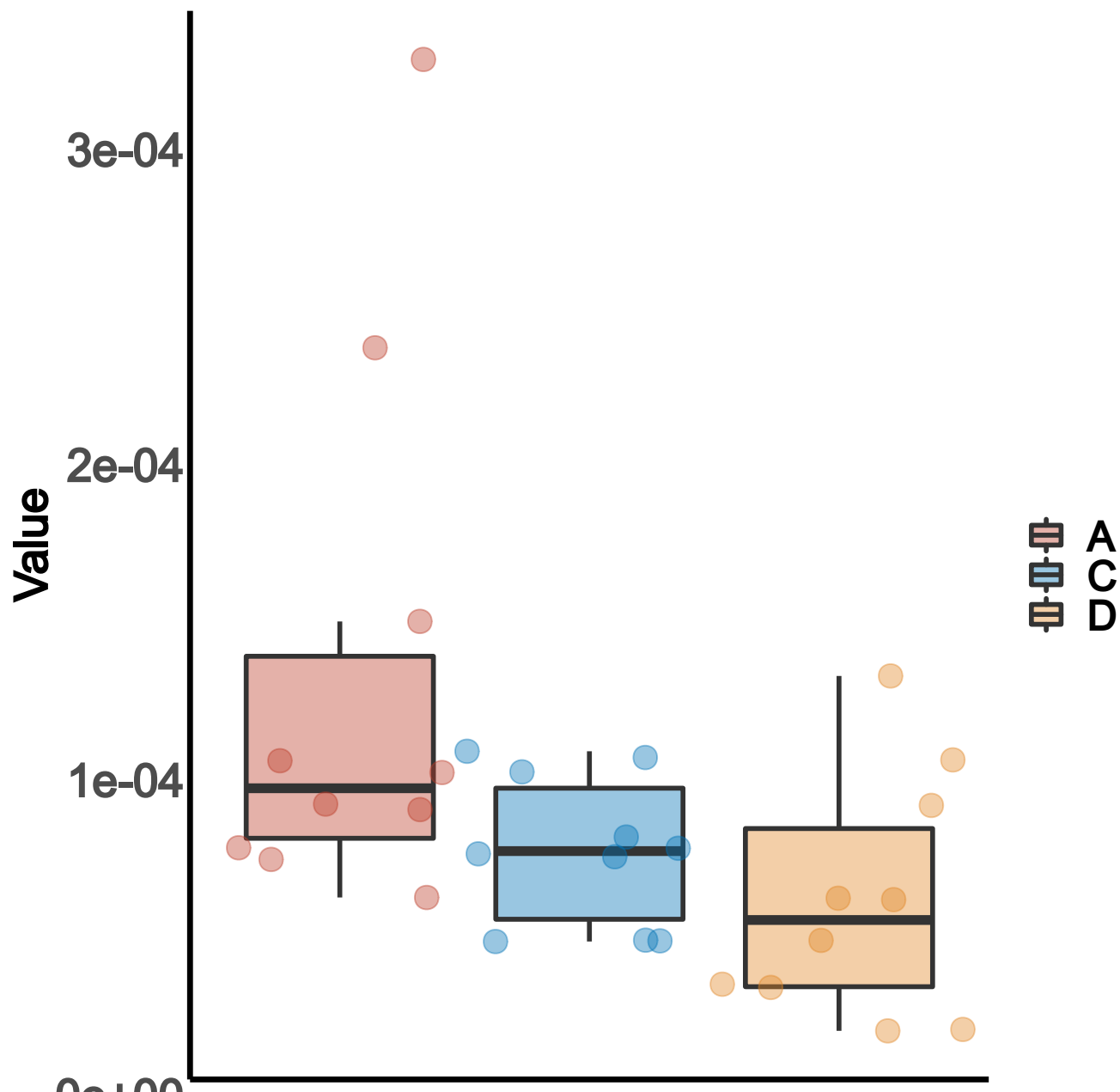

Supplement: Supplementary file 5 [file Data_Sheet_1.ZIP › boxplot/index65_boxplot_ANOVA.pdf]

**p-value = 0.022; n = 30**

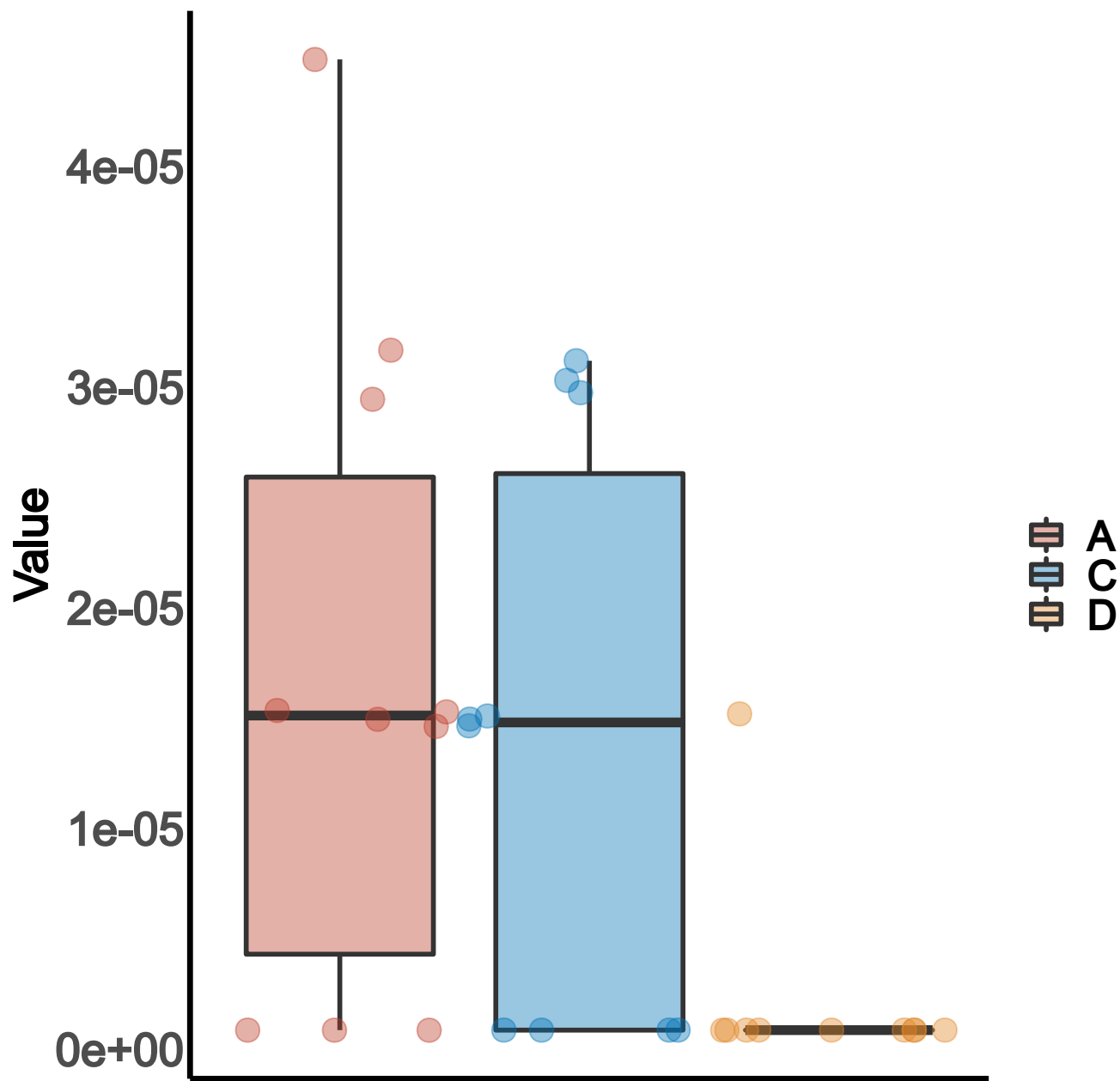

Supplement: Supplementary file 5 [file Data_Sheet_1.ZIP › boxplot/index66_boxplot_ANOVA.pdf]

p-value = 0.025; n = 30

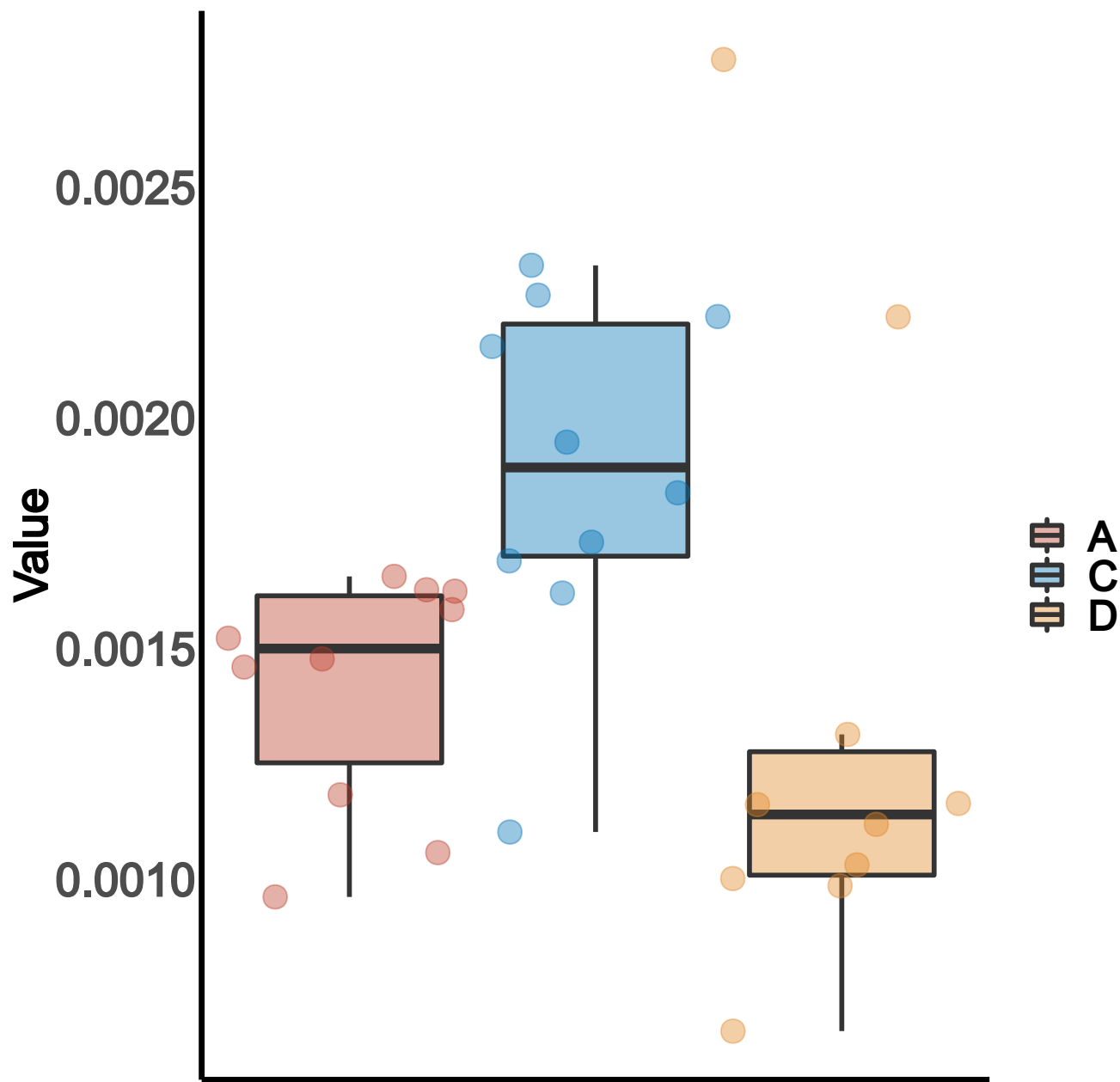

Supplement: Supplementary file 5 [file Data_Sheet_1.ZIP › boxplot/index67_boxplot_ANOVA.pdf]

p-value = 0.027; n = 30

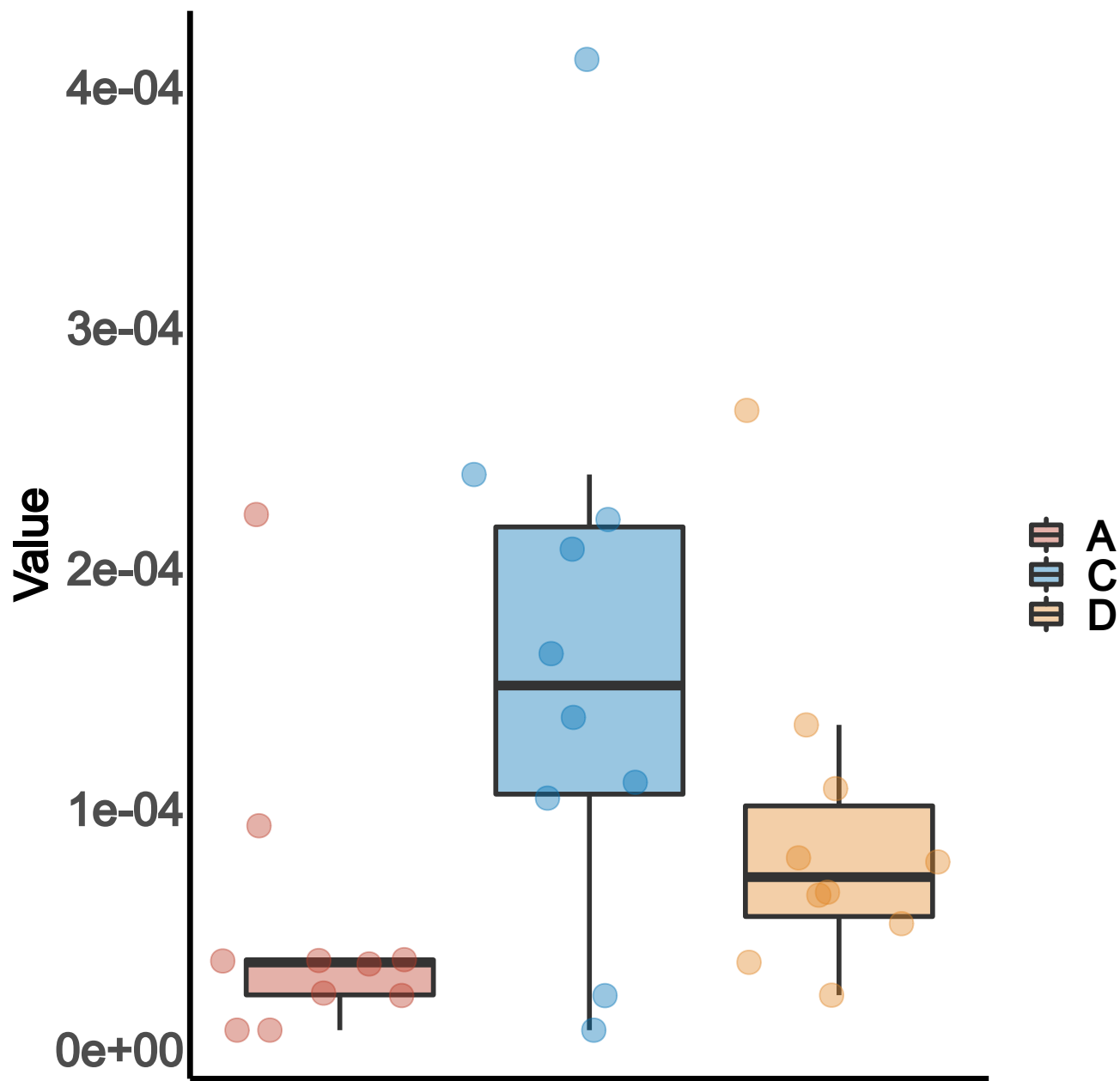

Supplement: Supplementary file 5 [file Data_Sheet_1.ZIP › boxplot/index68_boxplot_ANOVA.pdf]

p-value = 0.027; n = 30

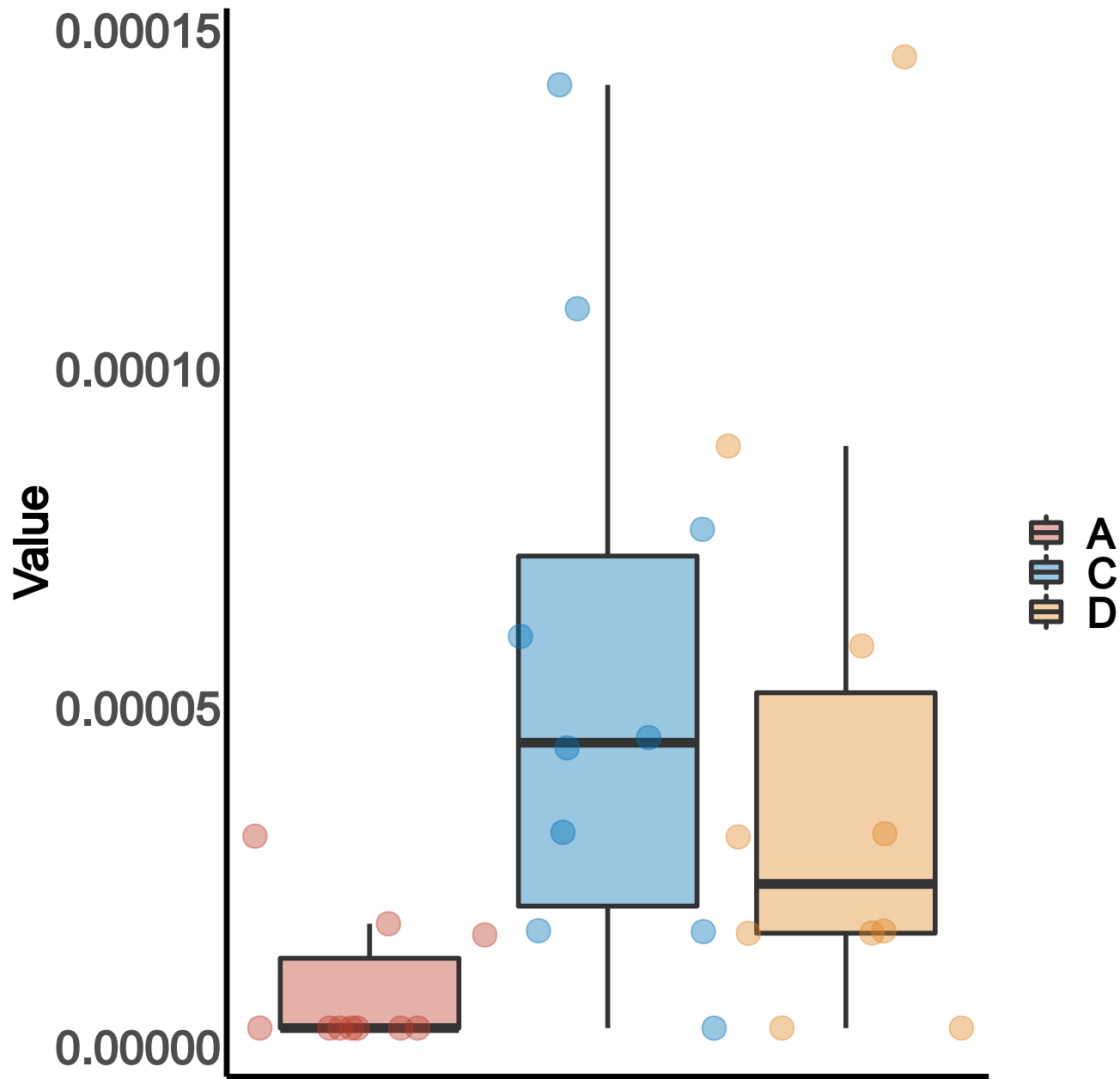

Supplement: Supplementary file 5 [file Data_Sheet_1.ZIP › boxplot/index69_boxplot_ANOVA.pdf]

p-value =  $1.5e-06$ ; n = 30

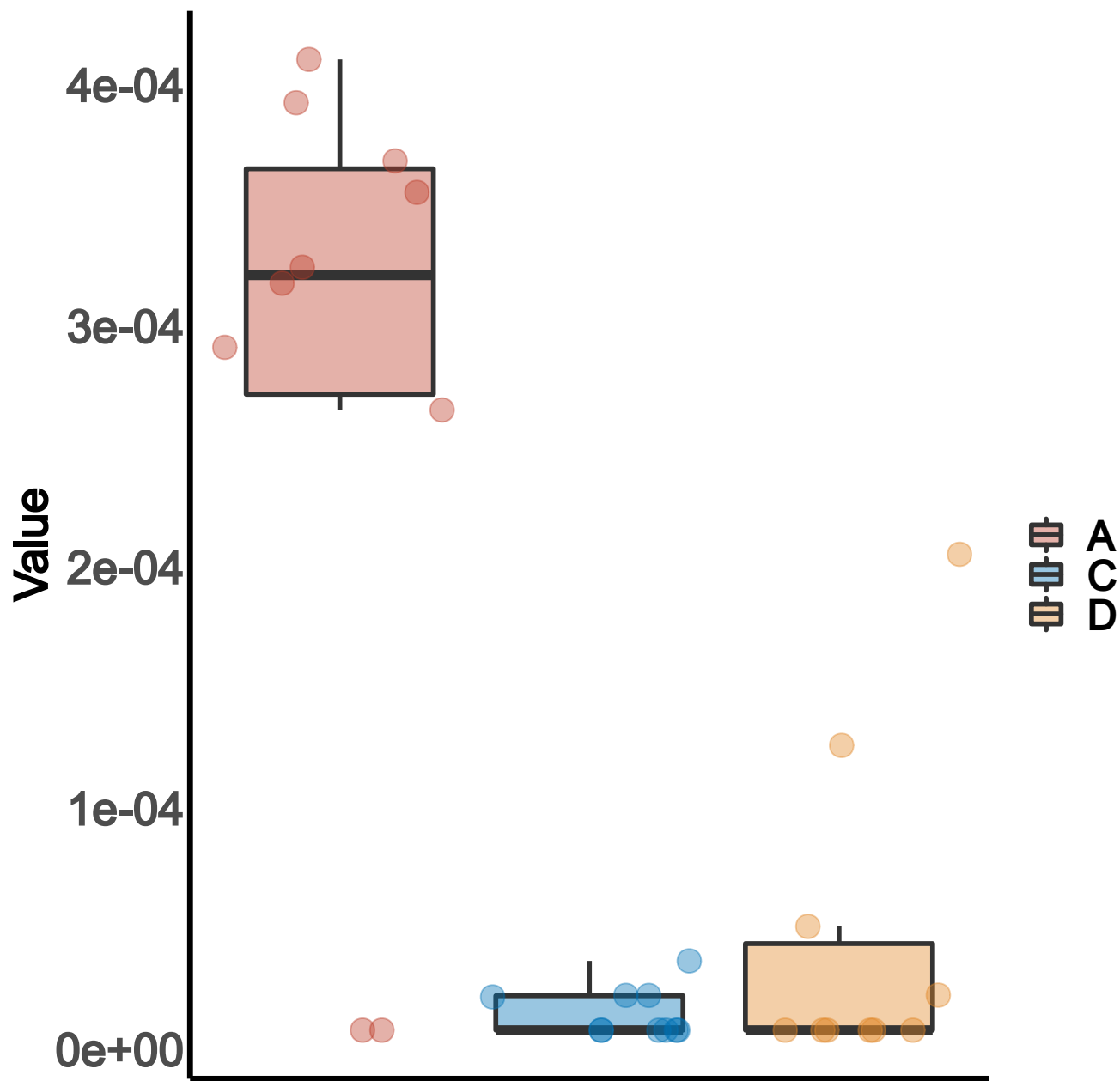

Supplement: Supplementary file 5 [file Data_Sheet_1.ZIP › boxplot/index6_boxplot_ANOVA.pdf]

**p-value = 0.029; n = 30**

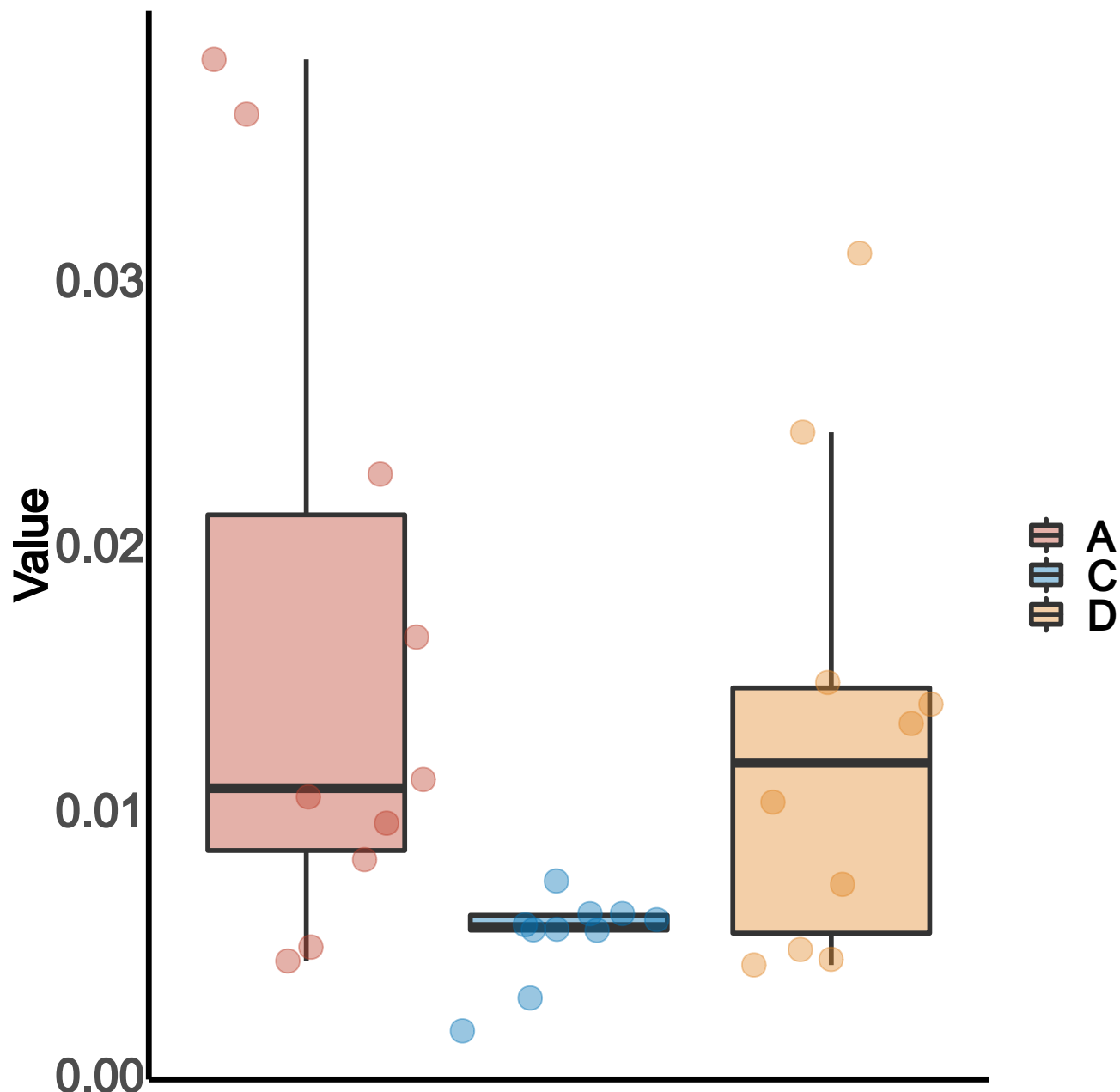

Supplement: Supplementary file 5 [file Data_Sheet_1.ZIP › boxplot/index70_boxplot_ANOVA.pdf]

p-value = 0.029; n = 30

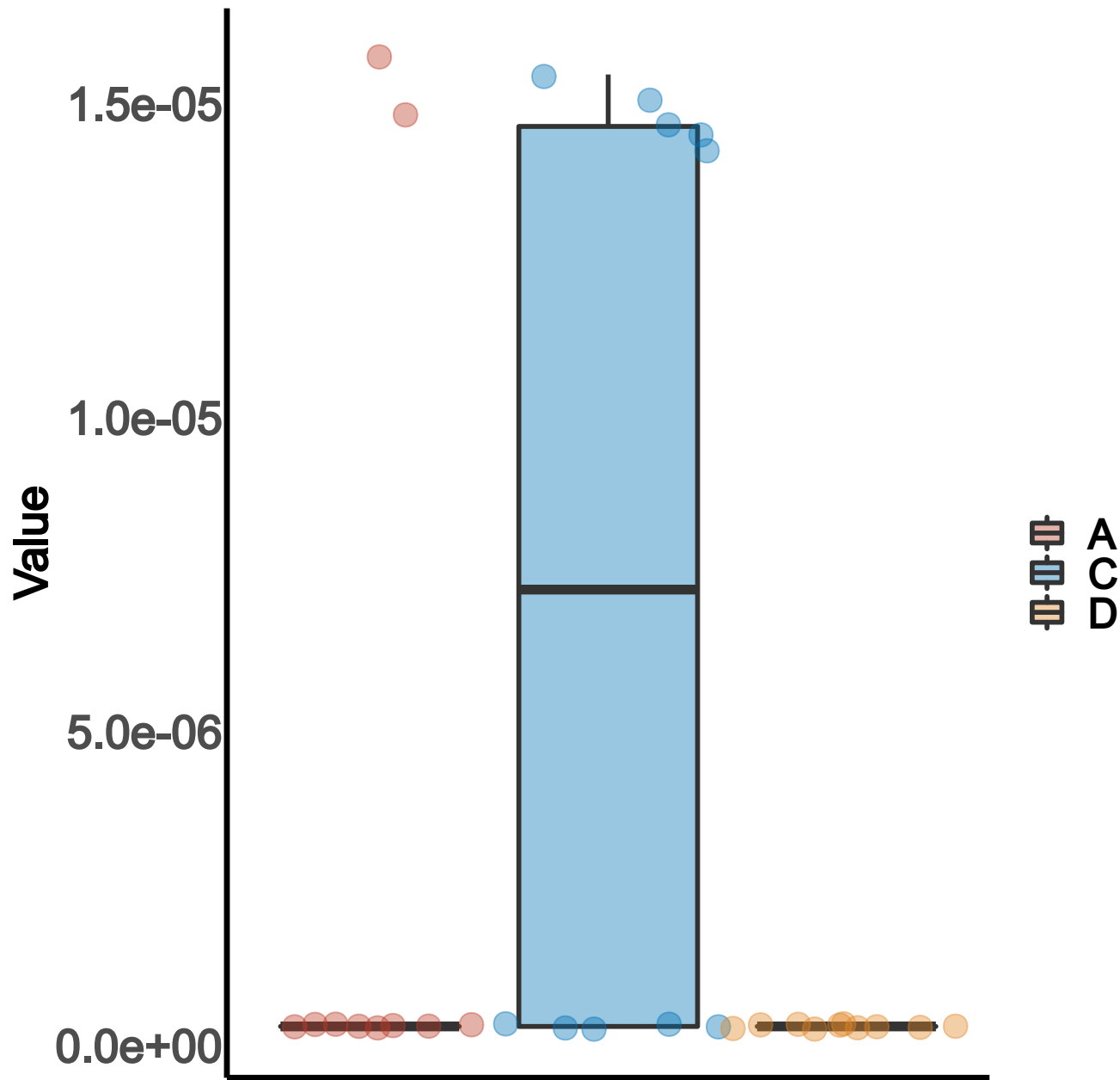

Supplement: Supplementary file 5 [file Data_Sheet_1.ZIP › boxplot/index71_boxplot_ANOVA.pdf]

p-value = 0.031; n = 30

Value

0e+00

1e-05

2e-05

3e-05

4e-05

A  
C  
D

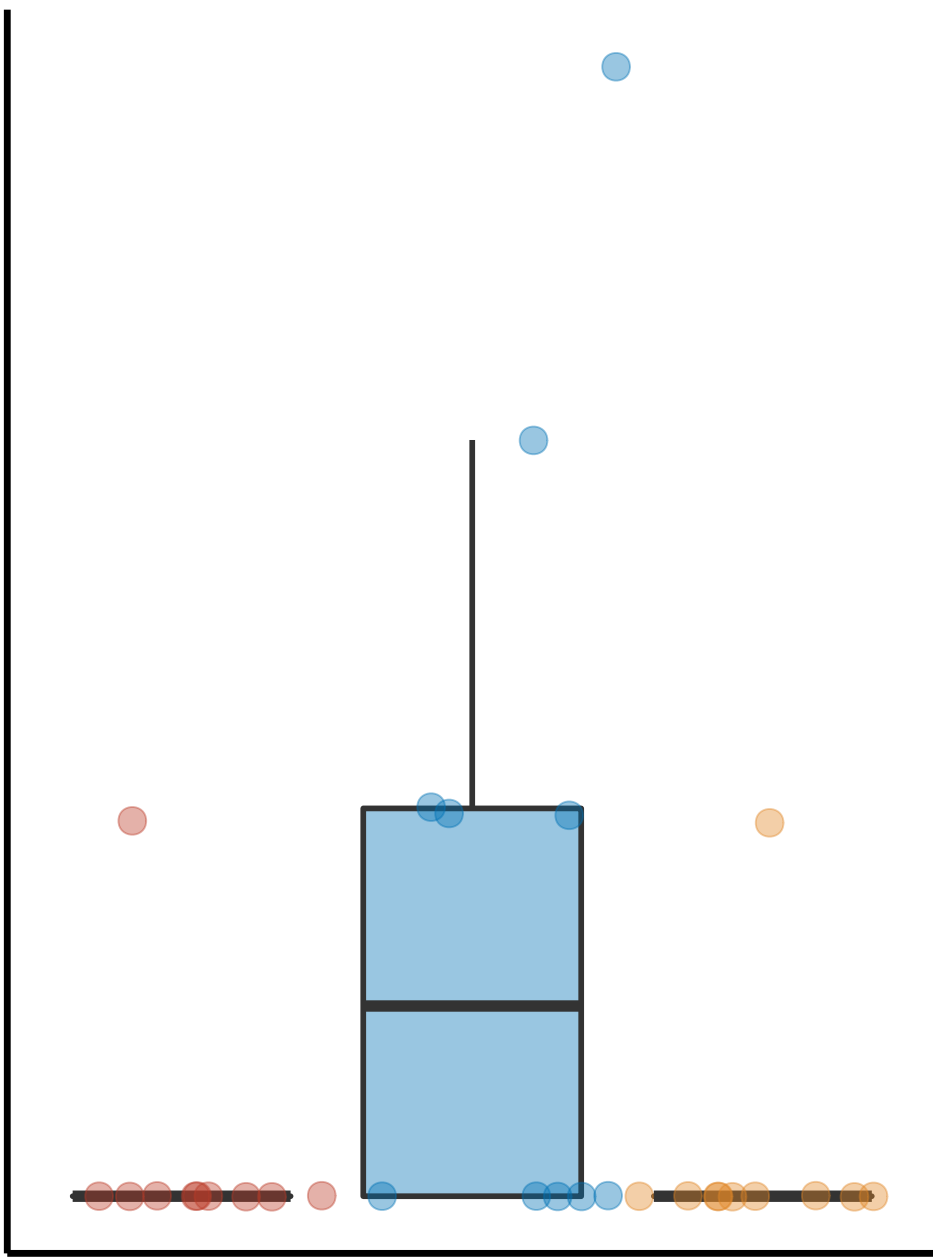

Supplement: Supplementary file 5 [file Data_Sheet_1.ZIP › boxplot/index72_boxplot_ANOVA.pdf]

p-value = 0.034; n = 30

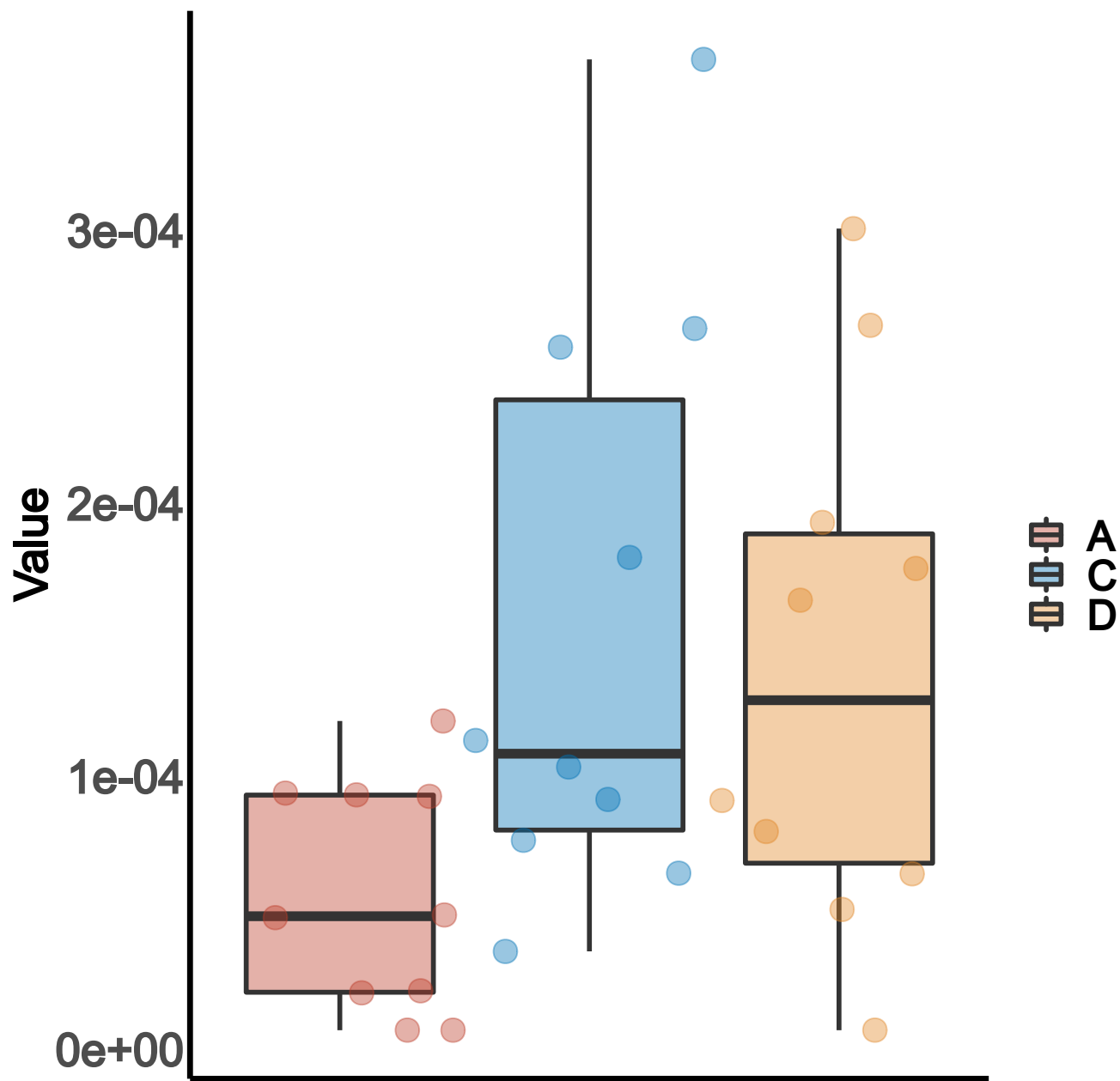

Supplement: Supplementary file 5 [file Data_Sheet_1.ZIP › boxplot/index74_boxplot_ANOVA.pdf]

**p-value = 0.034; n = 30**

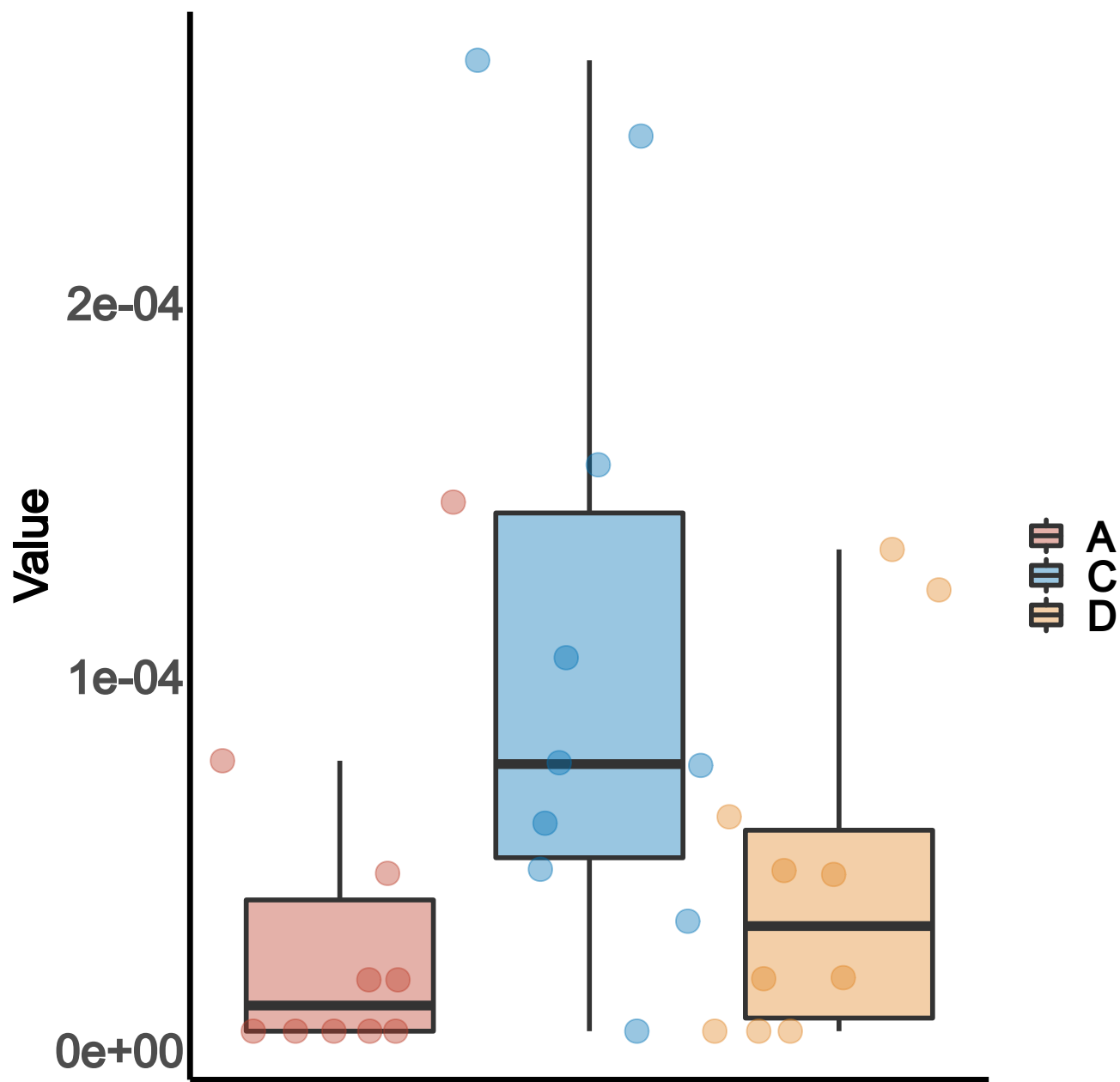

Supplement: Supplementary file 5 [file Data_Sheet_1.ZIP › boxplot/index75_boxplot_ANOVA.pdf]

**p-value = 0.036; n = 30**

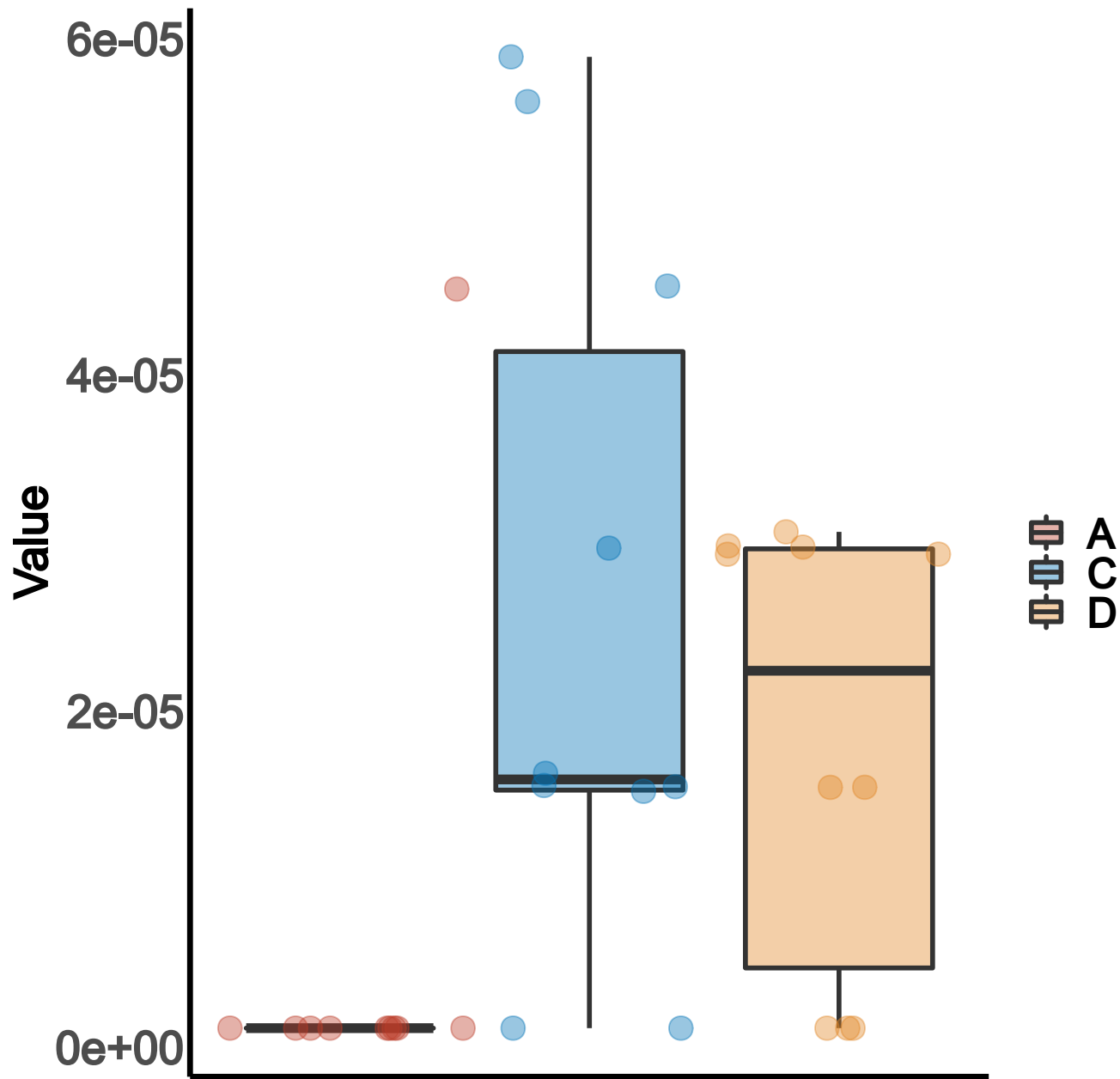

Supplement: Supplementary file 5 [file Data_Sheet_1.ZIP › boxplot/index76_boxplot_ANOVA.pdf]

**p-value = 0.039; n = 30**

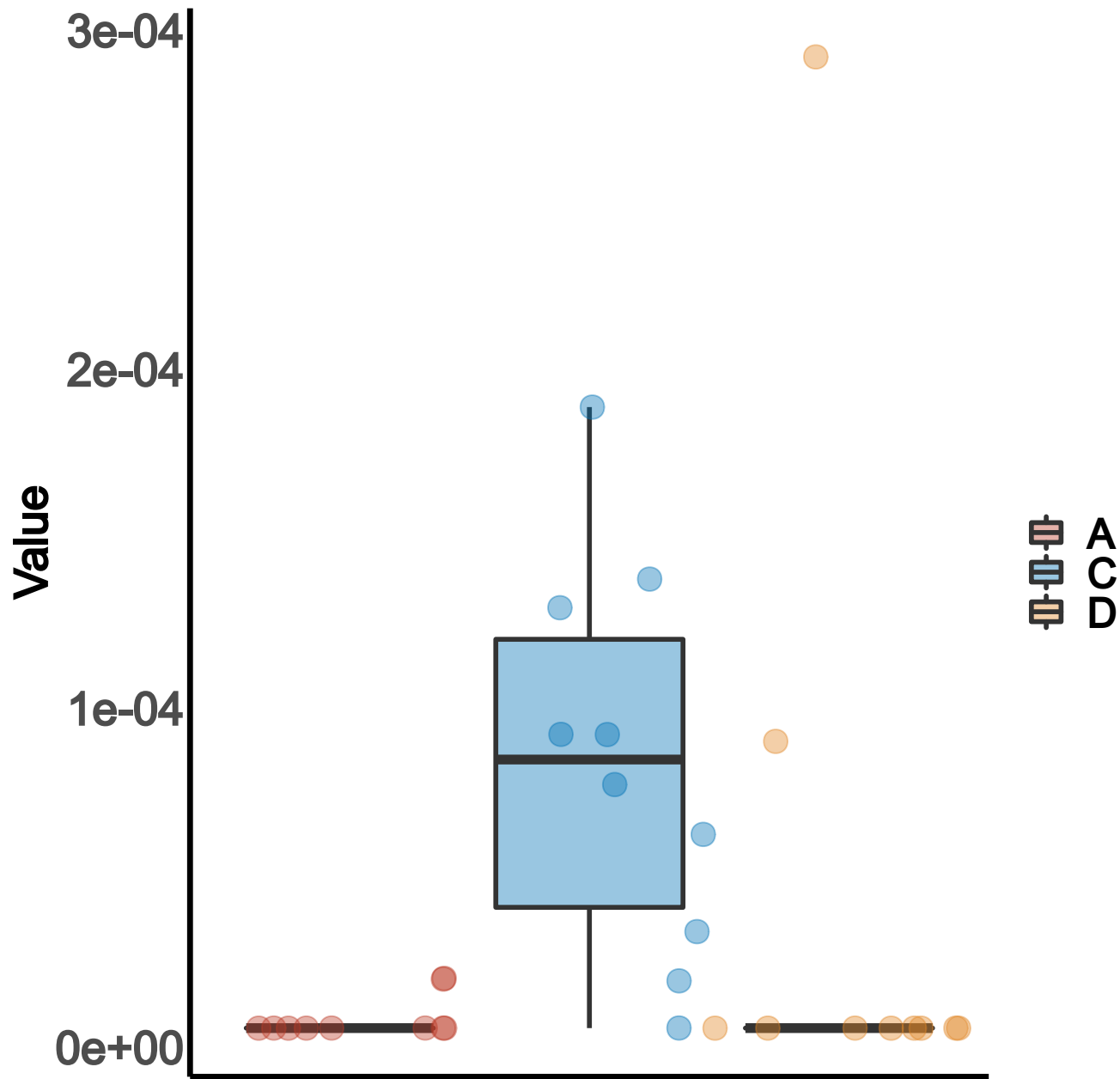

Supplement: Supplementary file 5 [file Data_Sheet_1.ZIP › boxplot/index77_boxplot_ANOVA.pdf]

p-value = 0.04; n = 30

Value

0.0015

0.0010

0.0005

0.0000

A  
C  
D

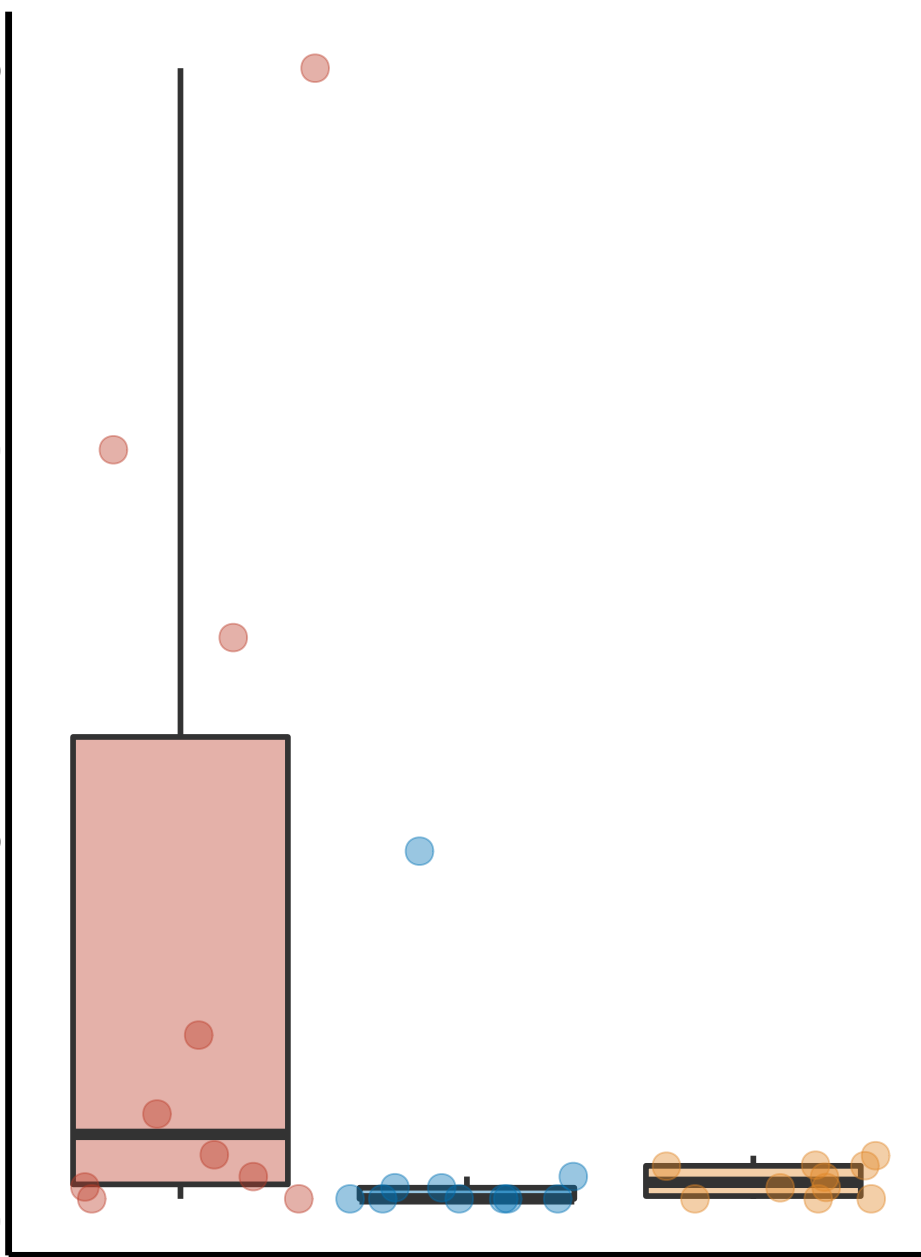

Supplement: Supplementary file 5 [file Data_Sheet_1.ZIP › boxplot/index78_boxplot_ANOVA.pdf]

**p-value = 0.041; n = 30**

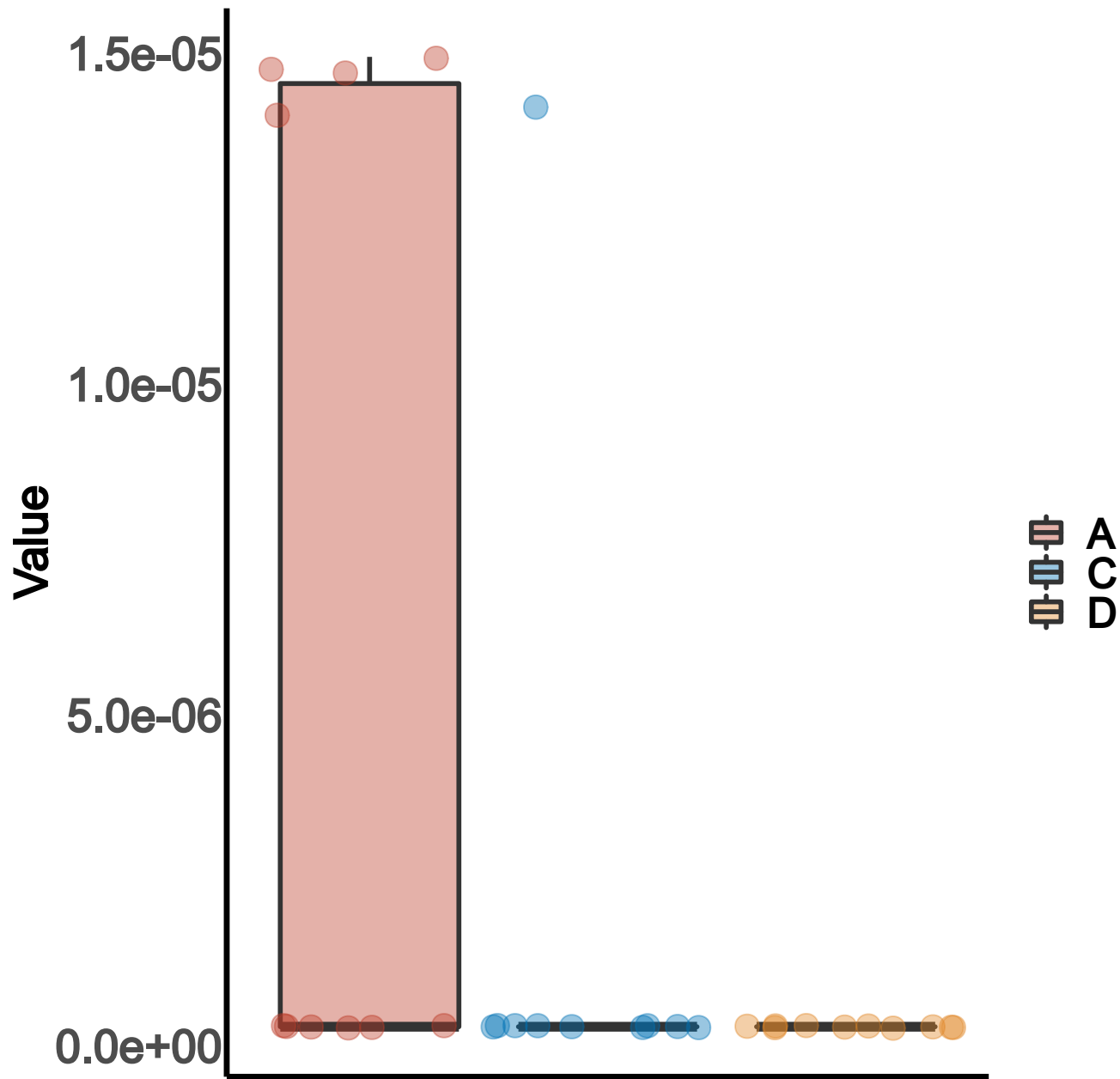

Supplement: Supplementary file 5 [file Data_Sheet_1.ZIP › boxplot/index79_boxplot_ANOVA.pdf]

p-value =  $1.8\text{e-}06$ ; n = 30

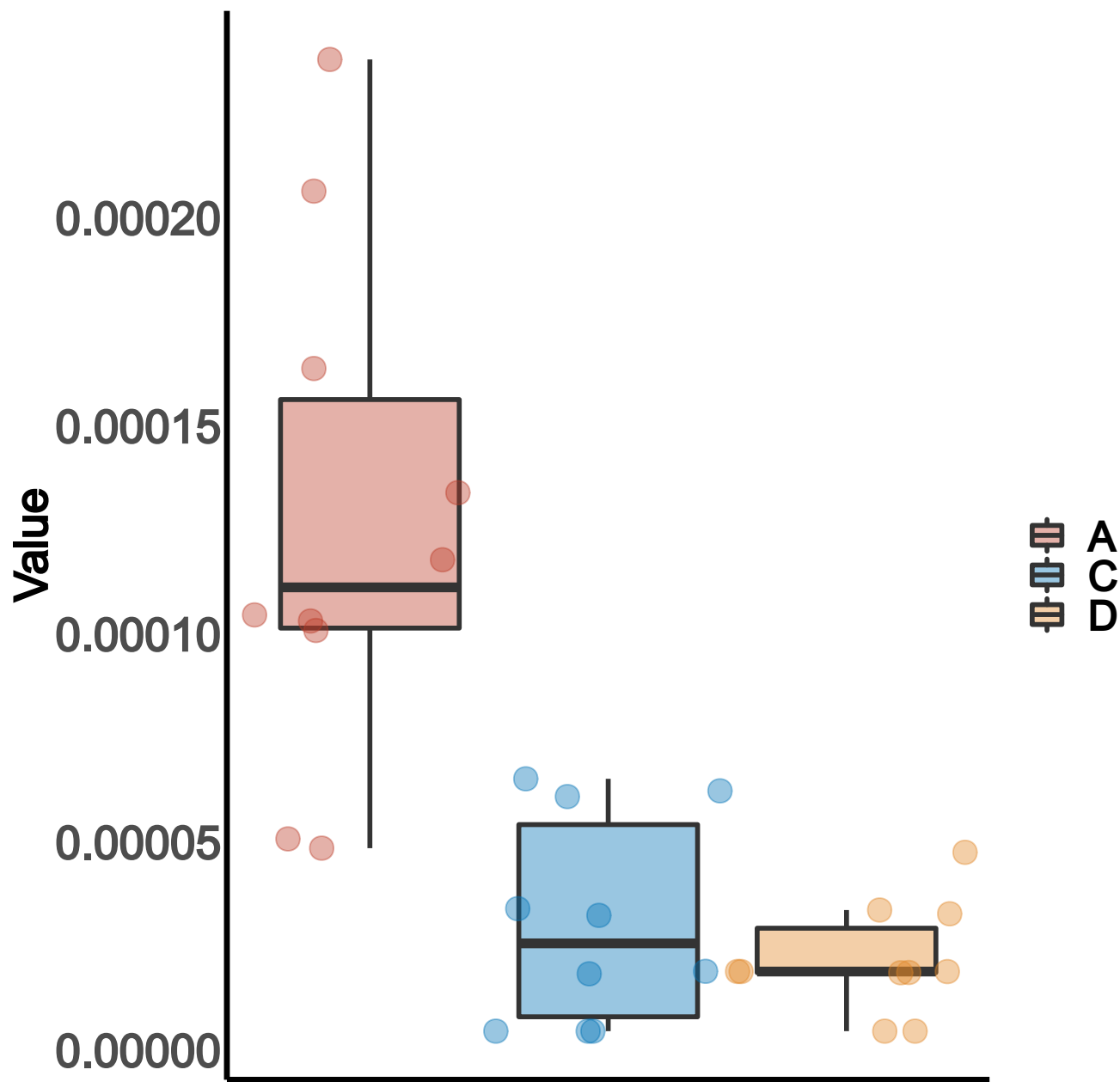

Supplement: Supplementary file 5 [file Data_Sheet_1.ZIP › boxplot/index7_boxplot_ANOVA.pdf]

p-value = 0.041; n = 30

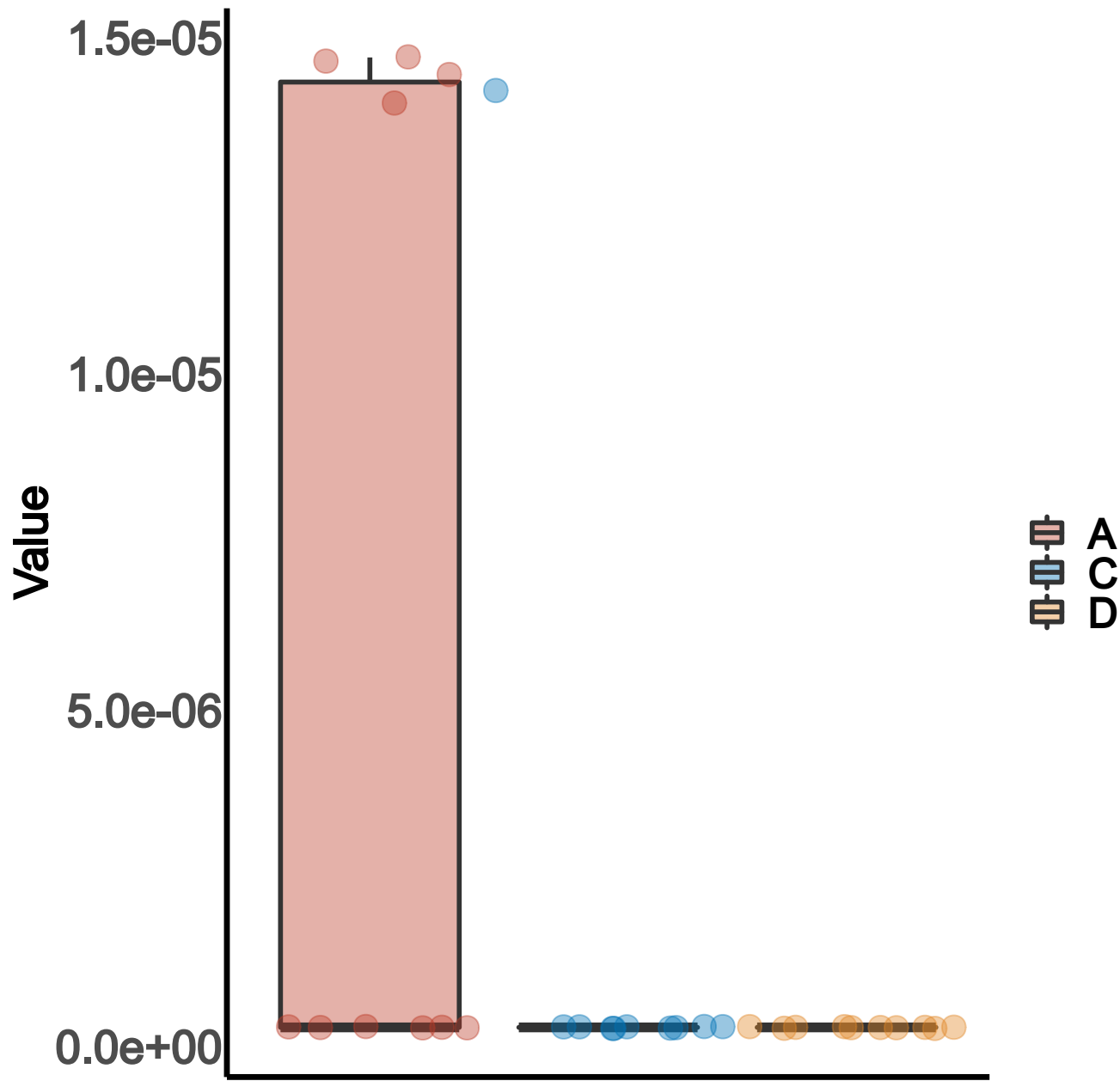

Supplement: Supplementary file 5 [file Data_Sheet_1.ZIP › boxplot/index80_boxplot_ANOVA.pdf]

**p-value = 0.042; n = 30**

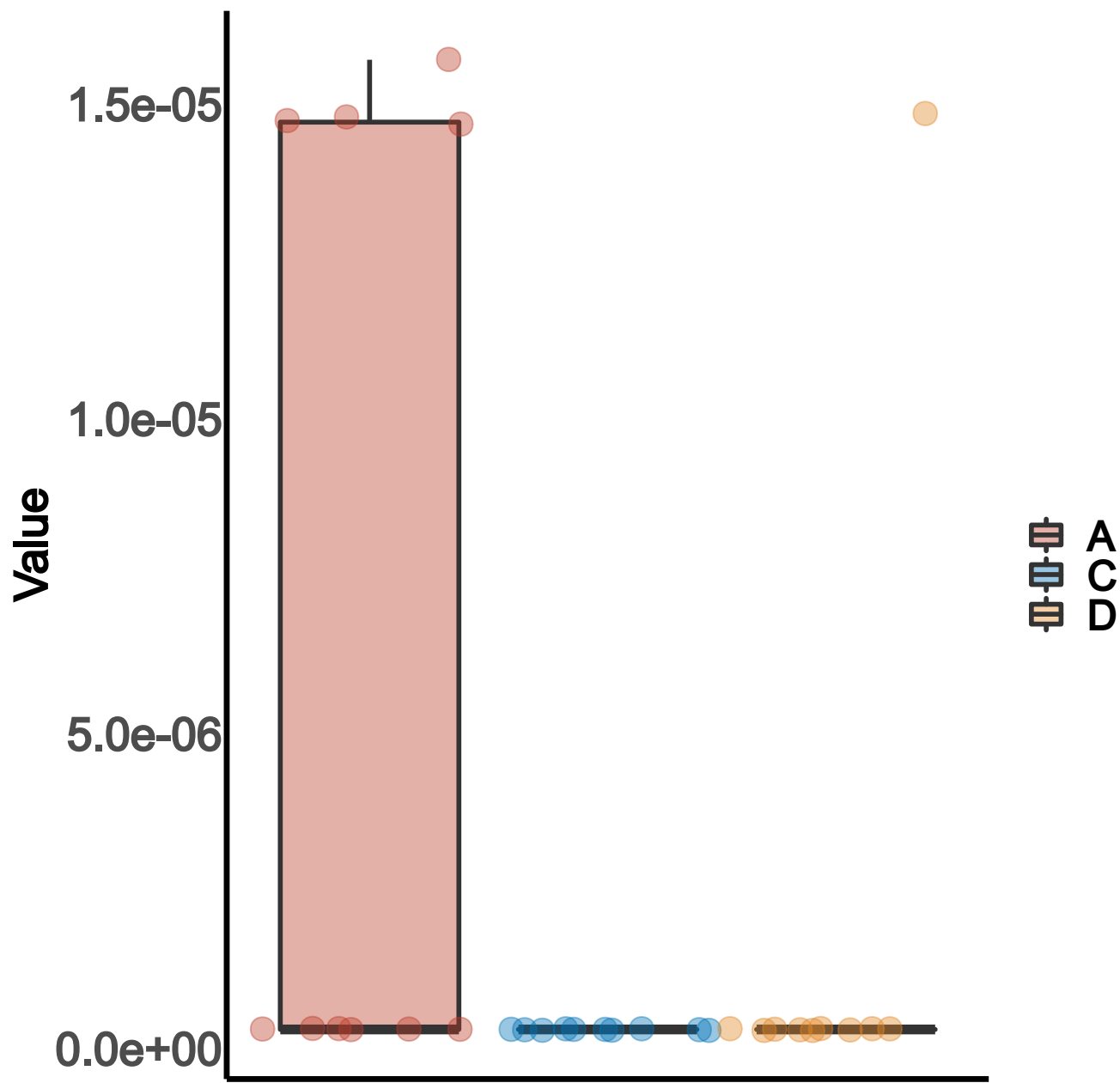

Supplement: Supplementary file 5 [file Data_Sheet_1.ZIP › boxplot/index82_boxplot_ANOVA.pdf]

p-value = 0.044; n = 30

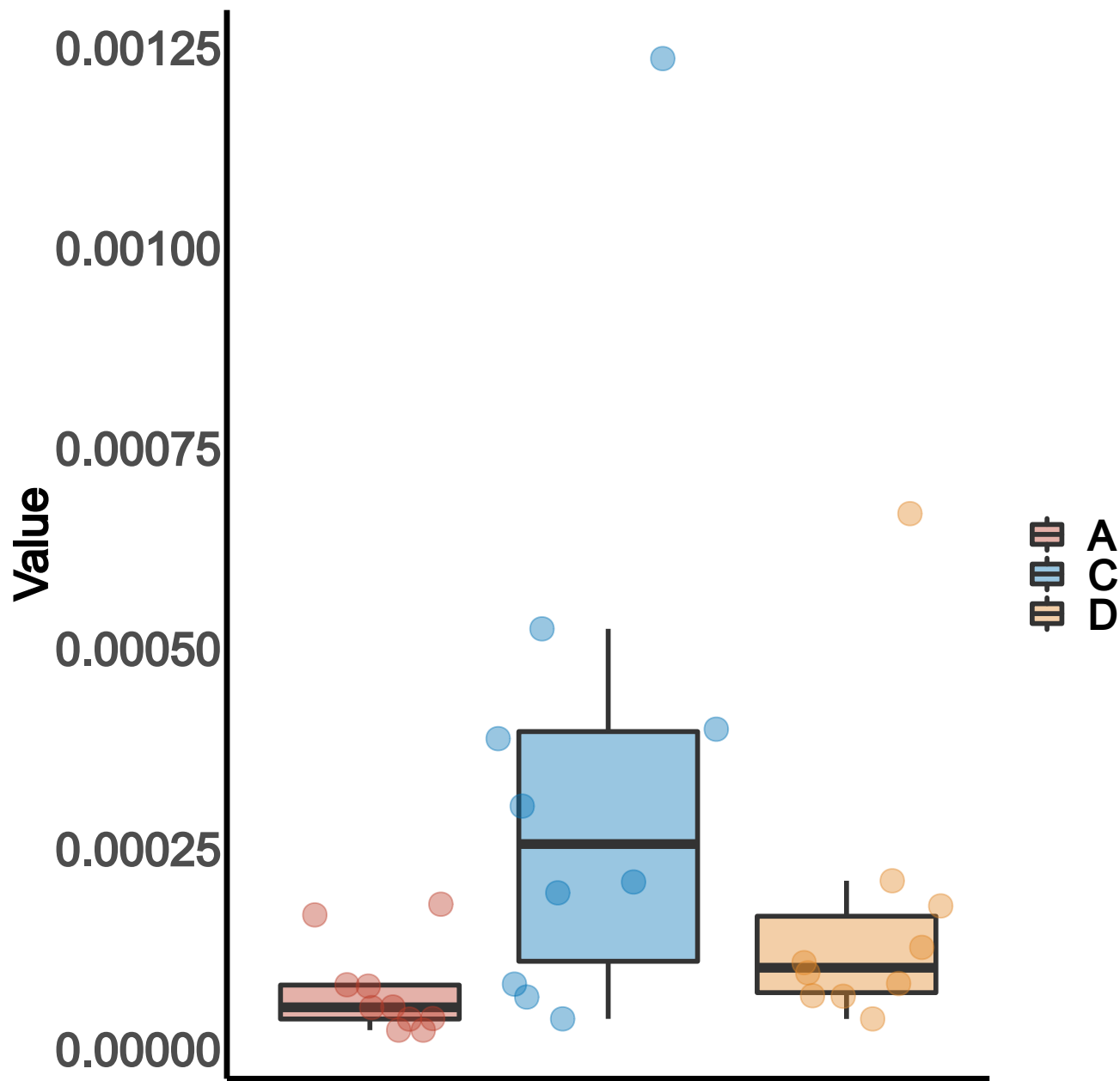

Supplement: Supplementary file 5 [file Data_Sheet_1.ZIP › boxplot/index83_boxplot_ANOVA.pdf]

p-value = 0.045; n = 30

Value

0.0075

0.0050

0.0025

0.0000

A  
C  
D

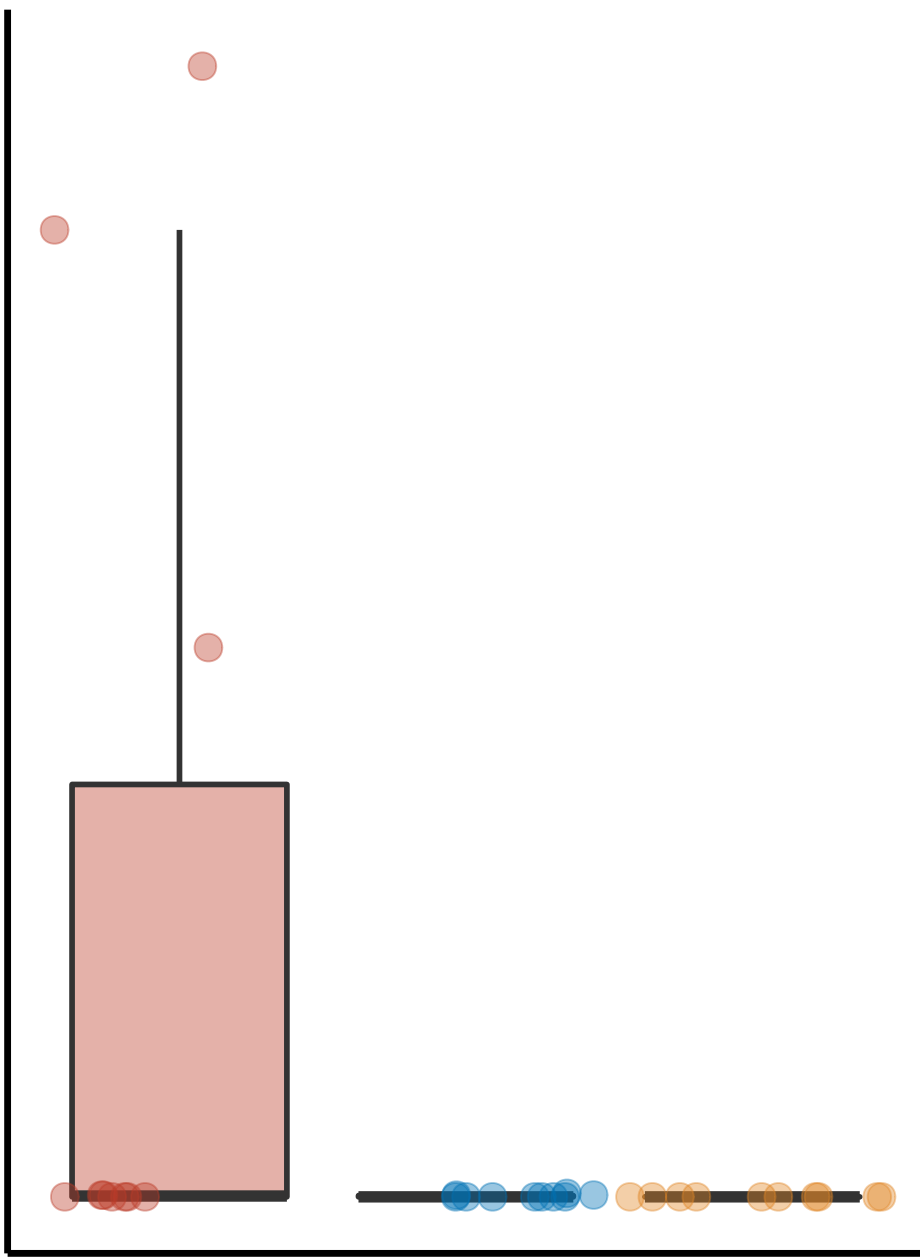

Supplement: Supplementary file 5 [file Data_Sheet_1.ZIP › boxplot/index84_boxplot_ANOVA.pdf]

p-value = 0.045; n = 30

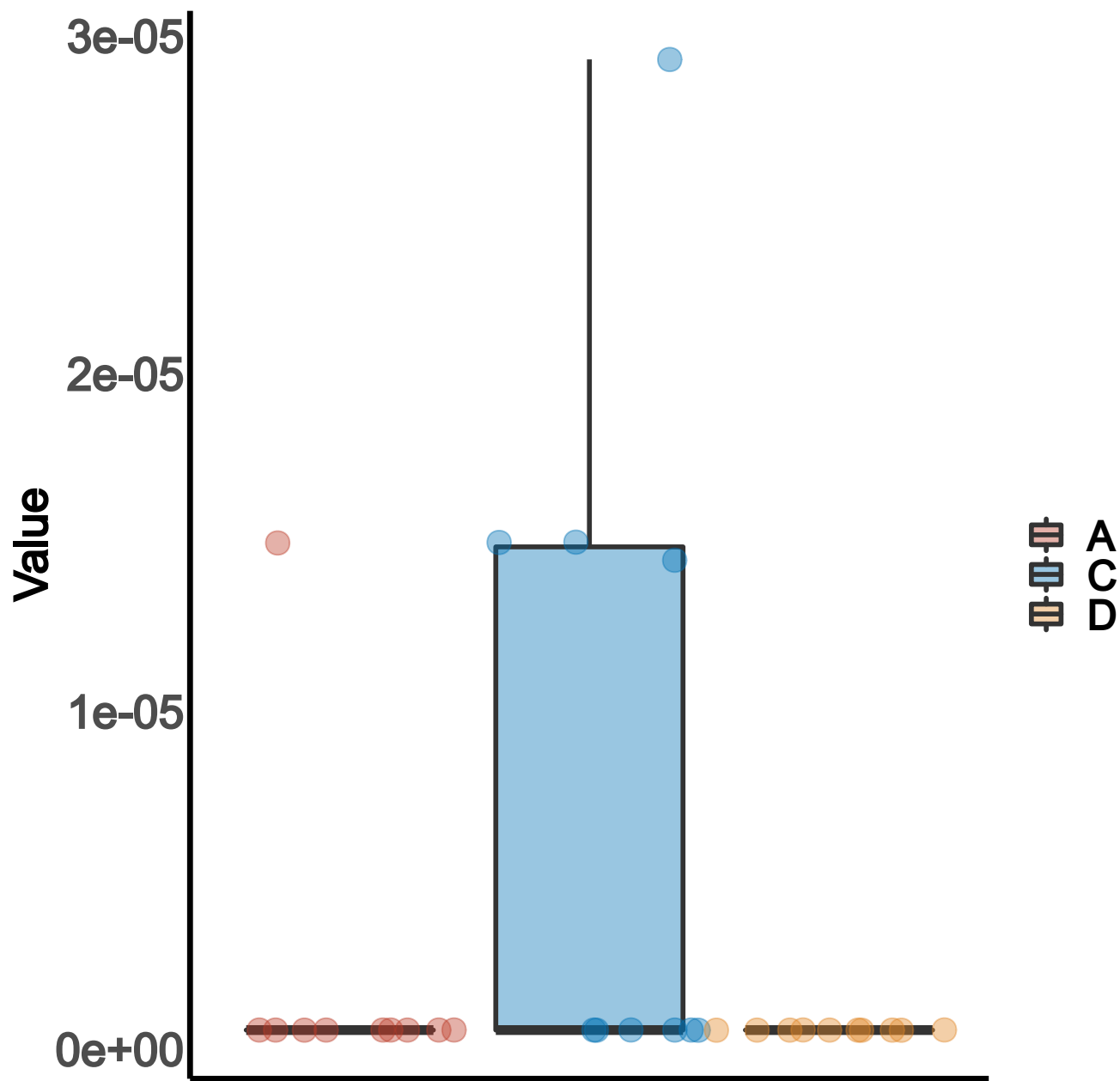

Supplement: Supplementary file 5 [file Data_Sheet_1.ZIP › boxplot/index85_boxplot_ANOVA.pdf]

**p-value = 0.047; n = 30**

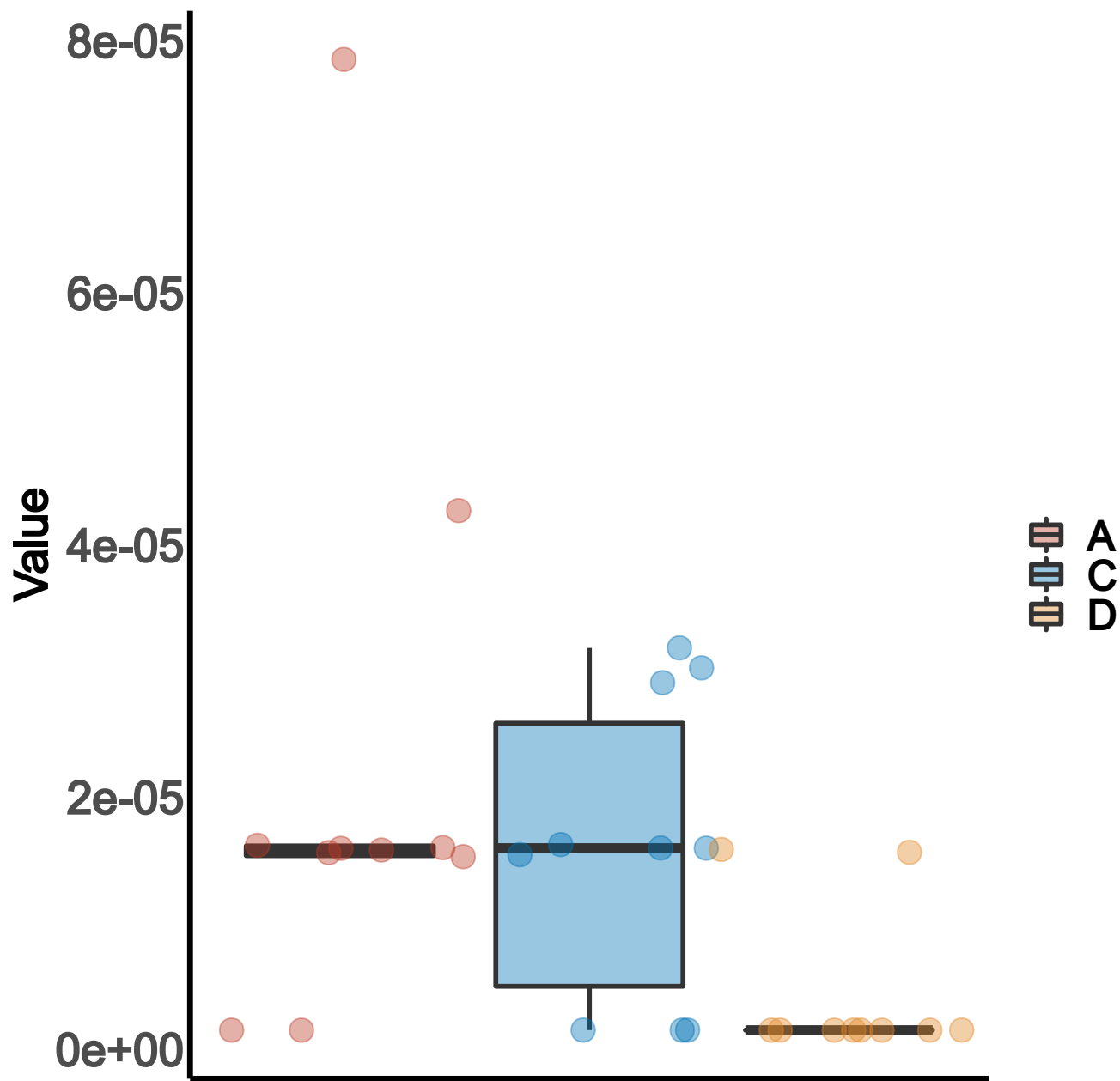

Supplement: Supplementary file 5 [file Data_Sheet_1.ZIP › boxplot/index86_boxplot_ANOVA.pdf]

p-value = 0.048; n = 30

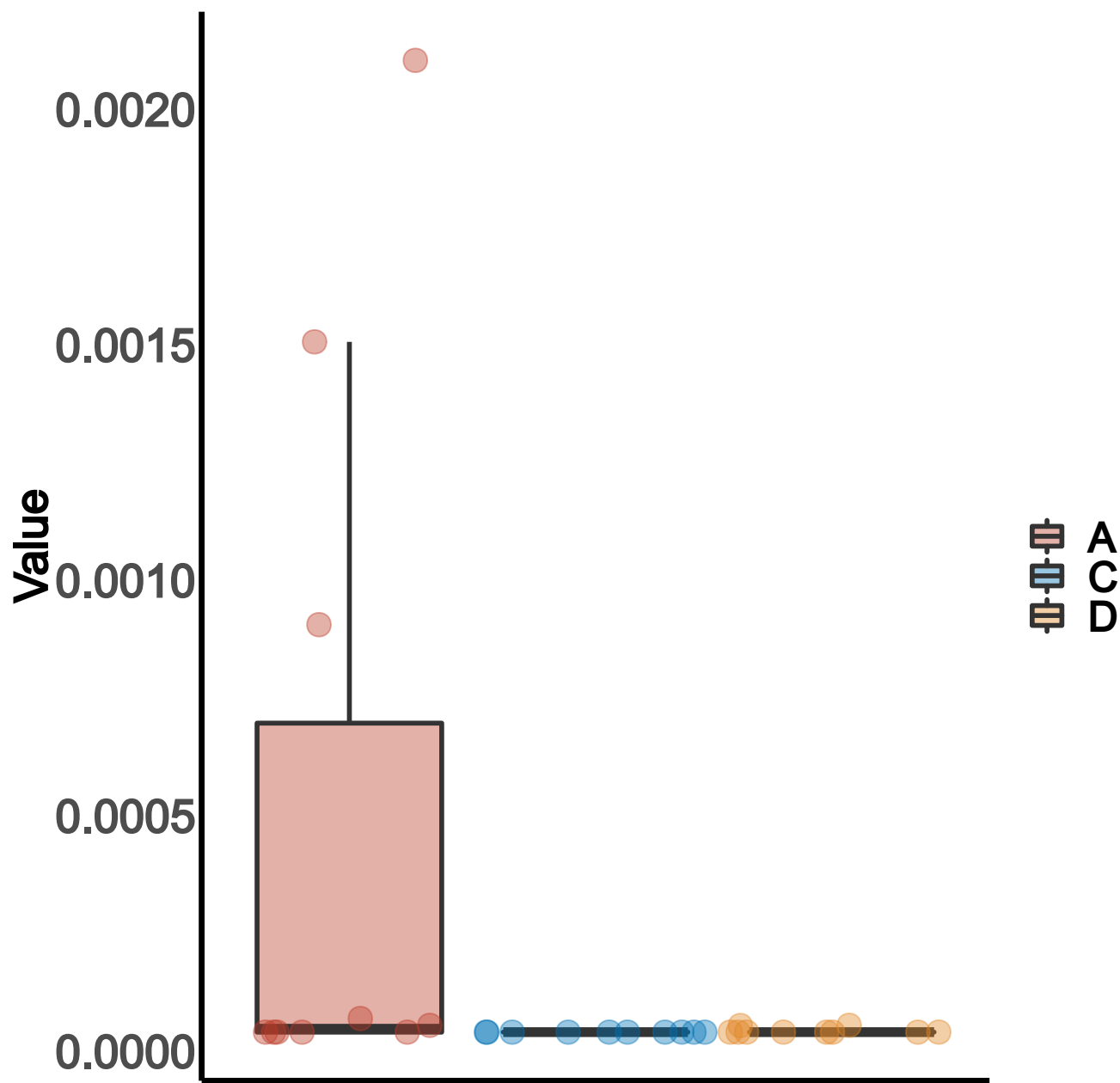

Supplement: Supplementary file 5 [file Data_Sheet_1.ZIP › boxplot/index87_boxplot_ANOVA.pdf]

p-value = 0.049; n = 30

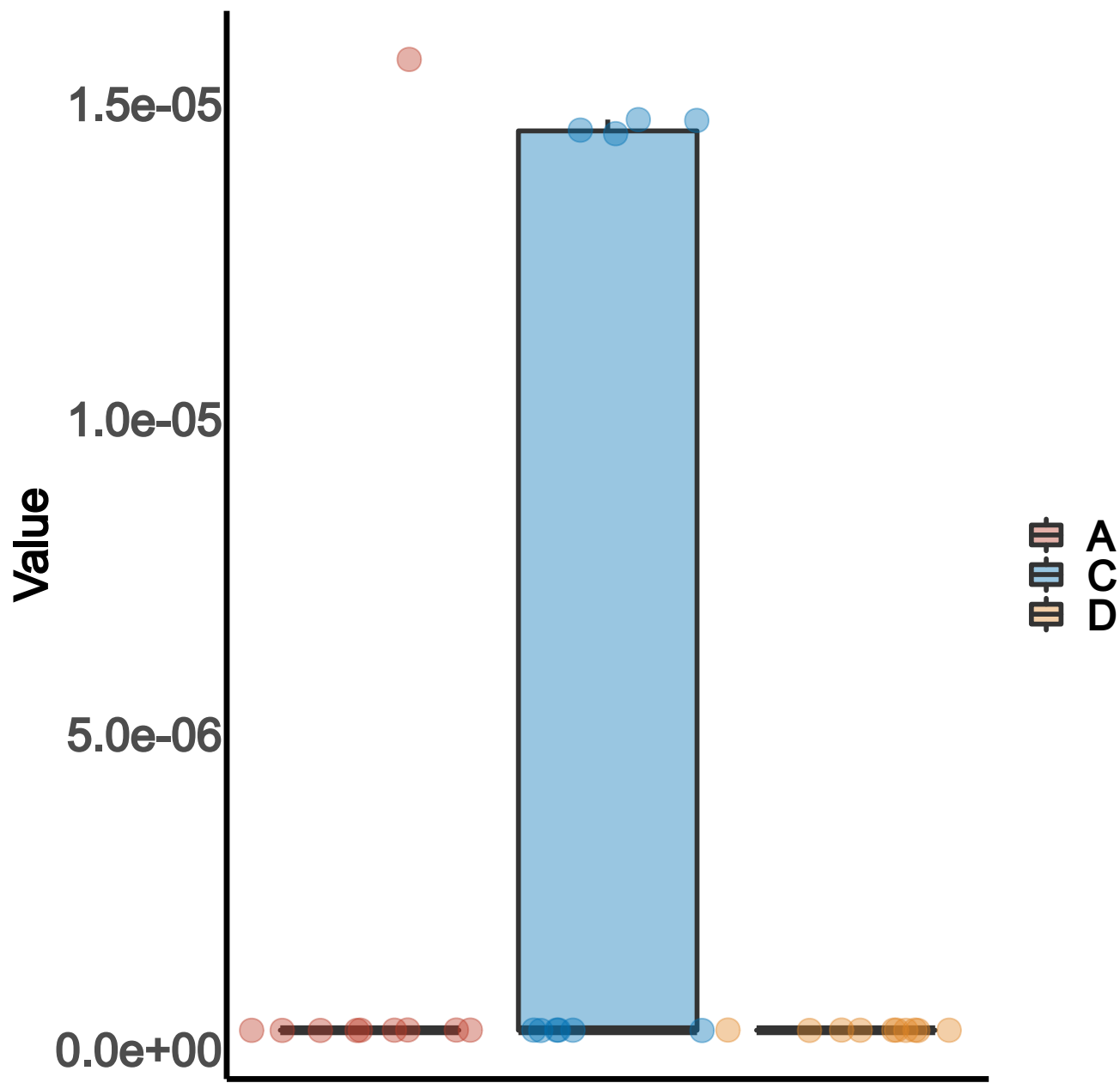

Supplement: Supplementary file 5 [file Data_Sheet_1.ZIP › boxplot/index88_boxplot_ANOVA.pdf]

p-value =  $2.3\text{e-}06$ ; n = 30

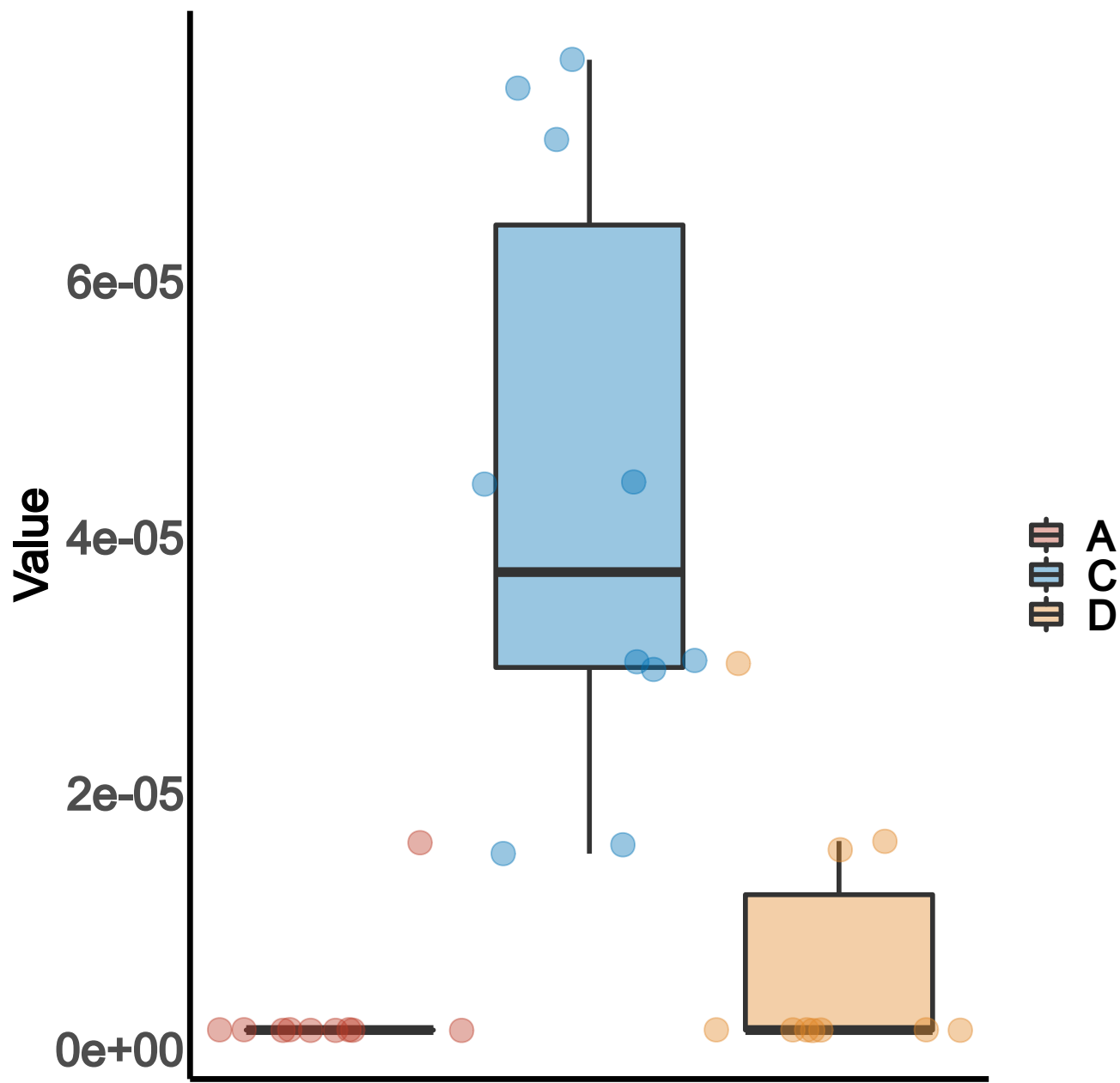

Supplement: Supplementary file 5 [file Data_Sheet_1.ZIP › boxplot/index8_boxplot_ANOVA.pdf]

p-value =  $9.6\text{e-}06$ ; n = 30

Value

0e+00

1e-04

2e-04

A  
C  
D

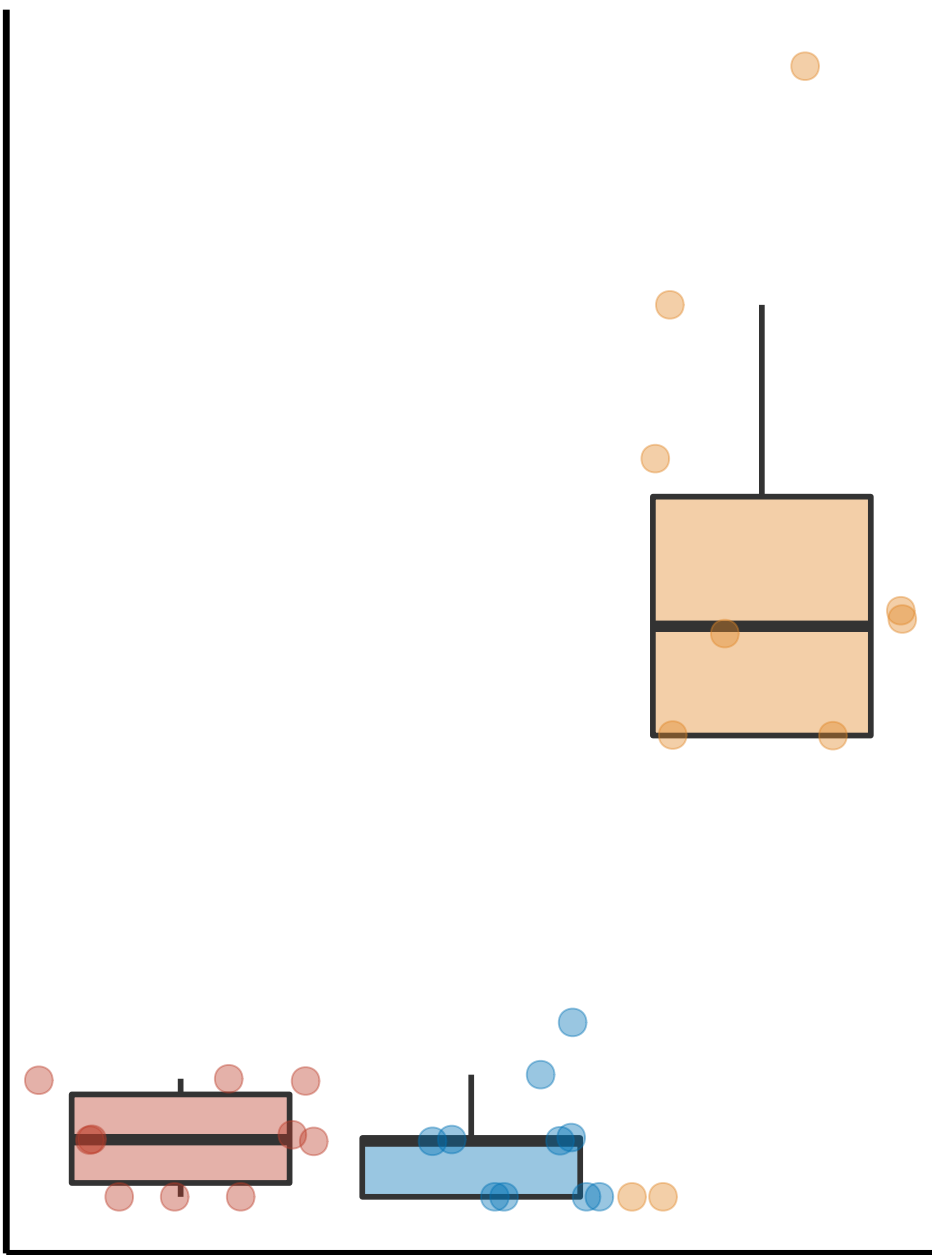

Supplement: Supplementary file 5 [file Data_Sheet_1.ZIP › boxplot/index9_boxplot_ANOVA.pdf]
